# Bisindole Alkaloids from a New Zealand Deep-Sea Marine Sponge *Lamellomorpha strongylata*

**DOI:** 10.3390/md17120683

**Published:** 2019-12-04

**Authors:** Kavita Ragini, Andrew M. Piggott, Peter Karuso

**Affiliations:** Department of Molecular Sciences, Macquarie University, Sydney, NSW 2109, Australia; kavita.ragini@mq.edu.au (K.R.); andrew.piggott@mq.edu.au (A.M.P.)

**Keywords:** *Lamellomorpha*, bisindole, hamacanthin, enantiodivergent, Methicillin-resistant *Staphylococcus aureus* (MRSA)

## Abstract

Chemical investigation of the secondary metabolites of a rare New Zealand deep-sea sponge, *Lamellomorpha strongylata*, resulted in the isolation of twenty-one indole alkaloids, including two new bisindoles—(*Z*)-coscinamide D (**1**), (*E*)-coscinamide D (**2**)—and four compounds isolated for the first time as natural products—lamellomorphamides A (**3**), B (**4**), C (**5**) and D (**6**). In addition, fifteen previously reported natural products were isolated, seven of which are seco analogs of hamacanthin alkaloids. The one sponge produces enantiomerically pure but opposite configurations of compounds that only differ in the number of bromines, suggesting enantiodivergent biosynthesis. In addition, four compounds were isolated as partial racemates, suggesting these compounds are biosynthesized via two independent routes.

## 1. Introduction

In our continuing exploration of marine sponges as sources of novel brominated alkaloids [1,2,3], we investigated a New Zealand deep-sea sponge collected from the western continental slope. *Lamellomorpha strongylata* belongs to the rare family Vulcanellidae within the order Tetractinellida [4]. There are only two reports of natural products from the genus *Lamellomorpha*, both from New Zealand from the same 1995 collection from the Chatham Rise, off the east coast of the South Island. Bioassay-guided separation of the extracts from that collection resulted in the isolation of three distinct classes of cytotoxic polyketides: calyculins (calyculins A, B, E, F, and calyculinamides A and B), swinholide H, and theonellapeptolide IIIe [5,6]. Surprisingly, our collection of the same species yielded no polyketides, rather indole alkaloids. In this paper, we report the isolation of four new bisindole alkaloids, as well as seventeen other indole alkaloids, from *L. strongylata*.

## 2. Results and Discussion

**Chart 1:**
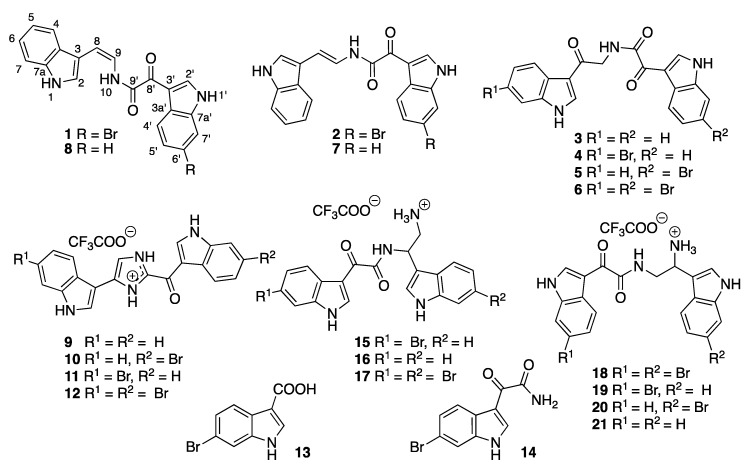
Structures of compounds **1**–**21**.

**Figure 1:**
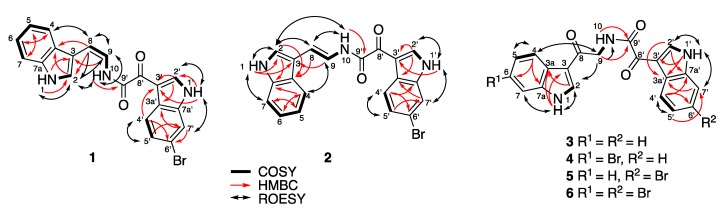
Key HMBC, COSY, and ROESY NMR correlations for **1**–**6**.

The freeze-dried sponge was sequentially extracted with hexane, ethyl acetate, and *n*-butanol, and twenty-one secondary metabolites (Chart 1) were isolated from the ethyl acetate and *n*-butanol extracts, including two new compounds, (*Z*)-coscinamide D (**1**) and (*E*)-coscinamide D (**2**). Lamellomorphamides A–D (**3**–**4**) were isolated for the first time as natural products, although they were previously reported as intermediates in the synthesis of 6′,6′′-didebromo-*cis*-3,4-dihydrohamacanthin B, 6′-debromo-*cis*-3,4-dihydrohamacanthin B, and hamacanthin analogues [7,8]. Eight previously reported compounds, (*E*)-coscinamide B (**7**) [9], (*Z*)-coscinamide B (**8**) [9], deoxytopsentin (**9**) [10], isobromodeoxytopsentin (**10**) [11], bromodeoxytopsentin (**11**) [11], dibromodeoxytopsentin (**12**) [11], 6-bromoindole-3-carboxylic acid (**13**) [12], and (6-bromo-1*H*-indol-3-yl) oxoacetamide (**14**) [13], and seven trifluoroacetic acid (TFA)-catalyzed artifacts of the hamacanthin A and B class of compounds, 3,4-seco-(8*R*)-6′′-debromohamacanthin A (**15**), 3,4-seco-(8*R*)-6′,6′′-didebromohamacanthin A (**16**), 3,4-seco-(8*S*)-hamacanthin A (**17**), 3,4-seco-(8*S*)-hamacanthin B (**18**), 3,4-seco-(8*S*)-6′′-debromohamacanthin B (**19**), 3,4-seco-(8*R*)-6′-debromohamacanthin B (**20**), and 3,4-seco-(8*R*)-6′,6′′-didebromohamacanthin B (**21**), were also isolated. The structures of **1**–**21** were determined by analysis of their spectroscopic data, including NMR in DMSO-*d*_6_, UV, IR, ECD, and MS in combination with high-level TDDFT calculations. The structures of the eight previously reported compounds **7**–**14** were also confirmed by comparison to literature data [9,10,11,12,13].

(*Z*)-coscinamide D (**1**) was isolated as a yellow amorphous solid. ESIMS analysis revealed a protonated molecular isotopic doublet at *m/z* 408/410 in a ratio of 1:1, characteristic of a monobrominated compound. HRESIMS analysis of **1** established a molecular formula of C_20_H_14_BrN_3_O_2_, based on the exact mass of the [M + Na]^+^ adduct ion (Δmmu −0.6). The presence of a 6-bromoindole moiety was apparent from a comparison of the ^1^H and ^13^C NMR (Table 1) and UV–vis spectroscopic data with those of the previously reported compound coscinamide B (**7**) [9]. Proton signals at δ_H_ 12.43 (1′-NH) and 8.92 (H-2′) and the ABX spin system comprised of signals at δ_H_ 7.41 (H-5′, dd, *J* = 8.4, 1.8 Hz), 7.76 (H-7′, d, *J* = 1.8 Hz), and 8.16 (H-4′, d, *J* = 8.4 Hz) indicated the presence of a 3,6-disubstituted indole moiety. The 1′-NH signal showed a COSY correlation with the H-2′ signal at δ_H_ 8.92, which in turn showed HMBC correlations to C-3′ (δ_C_ 111.8), C-3a′ (δ_C_ 125.3), and C-7a′ (δ_C_ 137.2) (Figure 1, Table S1). A COSY correlation was also observed between H-4′ and H-5′. Furthermore, 3-bond HMBC correlations from H-4′ to C-3′, and to the non-protonated carbons C-6′ (δ_C_ 116.1) and C-7a′ (δ_C_ 137.2), confirmed the attachment of bromine at C-6′ and established the 3,6-disubstituted bromoindole moiety. Another set of proton signals at δ_H_ 11.45 (1-NH), 7.64 (H-2), 7.43 (H-4), 7.16 (H-5), 7.07 (H-6), and 7.63 (H-7) was assigned to the 3-substituted indole residue. The 1-NH signal showed a COSY correlation with H-2, which in turn showed HMBC correlations to C-3 (δ_C_ 109.6), C-3a (δ_C_ 126.4), and C-7a (δ_C_ 135.8). COSY correlations defined the spin system from H-4 to H-7, confirming that **1** contained a 3-substituted indole. This interpretation was supported by 3-bond HMBC correlations from H-2 and H-4 to C-7a. IR absorption bands at 1670 and 1623 cm^−1^, along with non-protonated ^13^C NMR resonances at δ_C_ 159.7 and 180.0, suggest the presence of two carbonyl groups, one being an amide carbonyl. Another set of resonances was assigned to a vinylamide based on COSY correlations from H-9 to H-8 and 10-NH, and an HMBC correlation from 10-NH to the amide carbonyl carbon at C-9′ (δ_C_ 159.7). The observed ^3^*J*_H-8,H-9_ coupling constant (9.2 Hz) established the geometry of the olefinic bond as *Z*, which was supported by a strong ROESY correlation between H-8 and H-9. HMBC correlations from H-8 to C-2 (δ_C_ 123.8) and C-3a (δ_C_ 126.4), and from H-9 to C-3, indicated direct attachment of this fragment to the unsubstituted indole at C-3 (Figure 1). The remaining carbonyl substituent (δ_C_ 180.0) could be placed on C-3′ of the bromoindole moiety. Finally, the two indole-containing fragments were linked through the α-ketovinylamide to give the structure of **1**. While no HMBC correlations were observed to C-8′ (δ_C_ 180.0), the chemical shifts of **1** closely resembled those reported for coscinamides A and B (**7**) [9]. It should also be noted that there are no literature reports of HMBC correlations to C-8′ in analogous known compounds, even for experiments optimized for small range coupling constants [9].

(*E*)-Coscinamide D (**2**) was isolated as a yellow amorphous solid. HRESIMS analysis of **2** established the same molecular formula as **1** (C_20_H_14_BrN_3_O_2_) based on the deprotonated molecule [M − H]^−^ (Δmmu +0.2). The NMR data for **2** were very similar to those for **1** (Table 1), with the only significant differences being in the chemical shifts and coupling constants of the vinyl protons H-8 and H-9. The observed ^3^*J*_H-8,H-9_ coupling constant (14.7 Hz) suggested an *E* geometry, which was supported by the absence of a ROESY correlation between H-8 and H-9 (Figure 1, Table S2), confirming that **2** is the *E* isomer of **1**. All other spectroscopic data were consistent with this structure.

Lamellomorphamide A (**3**) was isolated as a yellow amorphous solid. HRESIMS analysis revealed a protonated molecule [M + H]^+^ consistent with a molecular formula C_20_H_15_N_3_O_3_ (Δmmu −0.3). The NMR data for **3** (Table 2) also showed the presence of two 3-substituted indole residues, which was reminiscent of those of coscinamide B (**7**). The main difference from the coscinamide derivatives was the presence of an extra carbonyl carbon. IR absorption bands at 1670 and 1623 cm^−1^ along with non-protonated ^13^C NMR resonances at δ_C_ 163.8, 181.9, and 189.2, suggest the presence of one amide and two conjugated ketones. HMBC correlations from H-2 (δ_H_ 8.50, d, *J* = 3.2 Hz) to C-3a (δ_C_ 125.4) and C-7a (δ_C_ 136.3), and from 1-NH (δ_H_ 12.07) to C-3 (δ_C_ 113.9) and C-3a (δ_C_ 125.4), indicated that the pyrrole 1-NH and H-2 were connected to an ABMX aryl spin system comprised of the signals at δ_H_ 7.20 (H-5), 7.23 (H-6), 7.49 (H-7), and 8.16 (H-4) (Figure 1, Table S3). HMBC correlations from H-2′ (δ_H_ 8.82, d, *J* = 3.2 Hz) and 1′-NH (δ_H_ 12.26) to C-3a′ (δ_C_ 126.2) and C-7a′ (δ_C_ 136.4) indicated that the other pyrrole protons 1′-NH and H-2′ were connected to another ABMX aryl spin system comprised of the signals at δ_H_ 7.27 (H-5′), 7.28 (H-6′), 7.54 (H-7′) and 8.26 (H-4′). This confirmed the presence of the two 3-substituted indole moieties. An HMBC correlation from H-2 to C-8 (δ_C_ 189.2) indicated attachment of C-8 at C-3. The amide proton showed an HMBC correlation to its carbonyl C-9′ (δ_C_ 163.8) and a COSY correlation to methylene H_2_-9 (δ_H_ 4.63, d, *J* = 5.9 Hz), which in turn was coupled by HMBC to the carbonyl C-8. ROESY correlations between H-2 and H_2_-9, and between H_2_-9 and 10-NH (δ_H_ 8.91) verified the attachment of the fragment RCO–NH–CH_2_–CO on C-3 of the indole moiety (Figure 1). The remaining carbonyl substituent C-8′ (δ_C_ 181.9) was placed on C-3′ of the additional indole moiety, and the two indole-containing fragments were linked through the ketone (δ_C_ 181.9) and the amide carbonyl (δ_C_ 163.8) to give the structure of **3**. Analogous to **1**, and the literature [11,14], no HMBC correlations were observed to C-8′.

Lamellomorphamide B (**4**) was also isolated as a yellow, amorphous solid. ESIMS analysis revealed a protonated molecule isotopic cluster [M + H]^+^ at *m/z* 424/426 in the ratio 1:1, suggesting the presence of one bromine atom. HRESIMS of **4** established a molecular formula of C_20_H_14_BrN_3_O_3_, based on the [M + Na]^+^ adduct ion (Δmmu −0.3). The ^1^H NMR data for **4** were similar to **3**, indicating the same basic scaffold. The NMR data for **4** (Table 2) also showed the presence of one 3-substituted indole and one 3,6-disubstituted bromoindole. The main difference from **3** was the presence of a bromine atom, and its attachment on the indole ring of the “left hand” of the compound was confirmed by HMBC and ROESY NMR experiments (Figure 1, Table S4). The 1-NH signal (δ_H_ 12.17), the methine H-2 (δ_H_ 8.53), and the AMX spin system comprised of the signals at δ_H_ 7.35 (H-5, dd, *J* = 8.5, 1.8 Hz), 7.69 (H-7, d, *J* =1.8 Hz), 8.09 (H-4, d, *J* = 8.5 Hz), indicated the presence of a 3,6-disubstituted indole moiety with the bromine atom located on C-6 (δ_C_ 115.0). HMBC correlations were observed from 1-NH, H-2, H-5, and H-7 to C-3a (δ_C_ 124.5) and from H-2 and H-4 to C-7a (δ_C_ 137.4). The connectivity between 3,6-disubstituted bromoindole to the carbonyl C-8 (δ_C_ 189.4) was established by a ROESY correlation between H-2 and H_2_-9 (δ_H_ 4.62). The proton signals at δ_H_ 12.25 (1′-NH), 8.80 (H-2′), 8.25 (H-4′), 7.27 (H-5′), 7.28 (H-6′), and 7.53 (H-7′) suggested the presence of a 3-substituted indole moiety. Attaching this second indole to the ketone C-8′ (δ_C_ 181.8) gave **4** as the only possible structure for lamellomorphamide B.

Lamellomorphamide C (**5**) was isolated as a yellow, amorphous solid. ESIMS analysis revealed a protonated molecule isotopic cluster [M + H]^+^ at *m/z* 424/426 in the ratio 1:1, indicating the presence of one bromine atom. HRESIMS of **5** established the same molecular formula as **4** (C_20_H_14_BrN_3_O_3_) based on the [M + Na]^+^ adduct ion (Δmmu −0.3). This immediately suggested that **5** is a regioisomer of **4**, with the bromine on the other (“right hand”) indole ring (Table 3). This interpretation was supported by ROESY correlations between 1′-NH (δ_H_ 12.32) and H-7′ (δ_H_ 7.75, d, *J* = 1.8 Hz) (Figure 1, Table S5). The presence of a small meta coupling between H-7′ and H-5′ (δ_H_ 7.42), and the absence of an ortho coupling revealed the attachment of the bromine atom at C-6′ (δ_C_ 116.0). The observed proton signals and coupling constants at δ_H_ 8.18 (H-4′, d, *J* = 8.4 Hz), 7.42 (H-5′, dd, *J* = 1.8, *J* = 8.4 Hz), and 7.75 (H-7′, d, *J* = 1.8 Hz), together with HMBC correlations from 1′-NH and H-2′ to C-3a′ (δ_C_ 125.3, is characteristic of a 3,6-disubstituted indole moiety with the bromine atom located on the indole ring of the “right hand” of **5**. The proton signals at δ_H_ 12.07 (1-NH), 8.50 (H-2), 8.16 (H-4), 7.20 (H-5), 7.23 (H-6), and 7.49 (H-7) suggest the presence of the 3-substituted indole moiety, which was confirmed by HMBC correlations from H-2 to C-3, C-3a, and C-7a, and from H-4 to C-3, C-6, and C-7a. The connection of 3-indole to C-8 was confirmed by a strong ROESY correlation between H-2 and H-9. The amide proton showed an HMBC correlation to its carbonyl C-9′ (δ_C_ 163.4) and a COSY correlation to methylene H_2_-9 (δ_H_ 4.63, *J* = 5.9 Hz), which in turn was coupled by HMBC to the carbonyl C-8. Comparison of the chemical shifts of the indole protons with those of **4**, together with HMBC correlations from H-2′ to C-3′, C-3a′, and C-7a′, and from H-4′ to C-3′, C-6′, and C-7a′, confirmed the connection of the 3′-bromoindolyl residue to C-8′, even though this carbon, like all others in this series, showed no correlations (Table S5). This left **5** as the only possible structure of lamellomorphamide C that matched the observed spectroscopic data.

Lamellomorphamide D (**6**) was isolated as a yellow, amorphous solid. ESIMS analysis revealed a protonated molecule isotopic cluster [M + H]^+^ at *m/z* 502/504/506 in the ratio 1:2:1, indicative of two bromine atoms. HRESIMS of **6** established a molecular formula of C_20_H_13_Br_2_N_3_O_3_, based on the [M + H]^+^ protonated molecule (Δmmu 0.0). The similarity in ^1^H NMR spectra between **6** and **3**–**5** suggested **6** had both indoles substituted with bromine. This was confirmed by the presence of two aromatic ABX systems; δ_H_ 7.35 (H-5, dd, *J* = 8.5, 1.8 Hz), 7.69 (H-7, d, *J* = 1.8 Hz), and 8.09 (H-4, d, *J* = 8.5 Hz); and δ_H_ 7.42 (H-5′, dd, *J* = 8.4, 1.8 Hz), 7.75 (H-7′, d, *J* = 1.8 Hz), and 8.18 (H-4′, d, *J* = 8.4 Hz). ROESY correlations between 1-NH (δ_H_ 12.17) and H-7 (δ_H_ 7.69, d, *J* = 1.8 Hz) and from 1′-NH (δ_H_ 12.32) and H-7′ (δ_H_ 7.75, d, *J* = 1.8 Hz) were observed (Figure 1, Table S6). The small meta coupling constants of H-7 and H-7′ to H-5 and H-5′, respectively, revealed the attachment of the bromine atoms at C-6 (δ_C_ 115.5) and C-6′ (δ_C_ 116.0), consistent with the previously identified structures. The remaining spectroscopic data (Table 3) were consistent with **6** being 6,6′-dibromolamellomorphamide A.

Compounds **3** and **4** have previously been reported as intermediates in the synthesis of *cis*-3,4-dihydrohamacanthin B [7], and compounds 5 and 6 have previously been reported as intermediates in the synthesis of hamacanthin B anologues [8] but to date have not been reported as natural products. The reported ^1^H and ^13^C NMR data for **4** matched our data exactly. Although no HMBC correlations to C-8′ were detected for any of the compounds **1**–**6**, the connectivity between the keto enamide and the indole moieties was determined by comparison of our NMR data with those of coscinamide B. The absence of any correlations to C-8′ is also consistent with the topsentins [11] and spongotines [14]. Coscinamides are the only known natural products that contain the unusual α-ketovinylamide functionality, and this is the first example of naturally occurring *E* isomers, although both coscinamide B and its *Z* isomer have been synthesized as intermediates in the synthesis of dihydrohamacanthins [15].

Deoxytopsentin (**9**) [10] was the major metabolite of *L. strongylata* and was obtained pure after Sephadex LH-20 gel permeation chromatography. As previously reported for topsentins [16], the doubling of ^1^H NMR signals was observed in DMSO-*d*_6_ due to slow tautomerization of the imidazole. The addition of a trace amount of TFA to the NMR sample resulted in one set of signals (Figure S47), but with broadening of the imidazolium methine. In methanol-*d*_4_ (Figure S48), all peaks were sharp and could be easily assigned.

Hamacanthins A (**24**) and B (**25**) [14,17] are part of a series of marine alkaloids that contain a six-membered nitrogen heterocycle (5,6-dihydro-2-(1*H*)-pyrazinone) as a linker unit between two variously substituted indole groups. Seven putative acid-catalyzed artifacts of the hamacanthin A and B class of compounds were isolated from the ethyl acetate and *n*-butanol extracts: 3,4-seco-6′′-debromohamacanthin A (**15**), 3,4-seco-6′,6′′-didebromohamacanthin A (**16**), 3,4-seco-hamacanthin A (**17**), 3,4-seco-hamacanthin B (**18**), 3,4-seco-6′′-debromohamacanthin B (**19**), 3,4-seco-6′-debromohamacanthin B (**20**), and seco-6′,6′′-didebromohamacanthin B (**21**). NMR spectroscopy of these HPLC purified compounds (containing traces of TFA) showed only one set of peaks corresponding to the acyclic form (Figures S56–S69, Tables S7–S9), while LC-MS analysis (with or without formic acid) showed two peaks (Figure S1). The first peak (*m/z* 425/427; λ_max_ 214, 262, 278, 329 nm) corresponded to the acyclic form and the second peak (*m/z* 407/409, Figure S75; λ_max_ 216, 280, 385 nm;) corresponded to the cyclic form (Figure S2). Compounds **15**–**21** were isolated by HPLC purification with 0.05% TFA in the mobile phase. An LC-MS analysis of crude *n*-butanol and ethyl acetate fractions did not reveal masses corresponding to the acyclic compounds (Figures S3 and S4), but rather the cyclic hamacanthin A and B class of compounds. We therefore propose that the TFA-catalyzed ring-opening occurred during HPLC purification of **15**–**21**. A similar mechanism has been proposed by Kuoko et al. [18] in the total synthesis of optically active hamacanthins A and B from the acyclic intermediates. Alternatively, the previously isolated hamacanthin A and B type alkaloids could be artifacts of isolation as previous reports all used silica gel chromatography, which we found efficiently cyclizes compounds **15**–**21**.

**Scheme 1:**
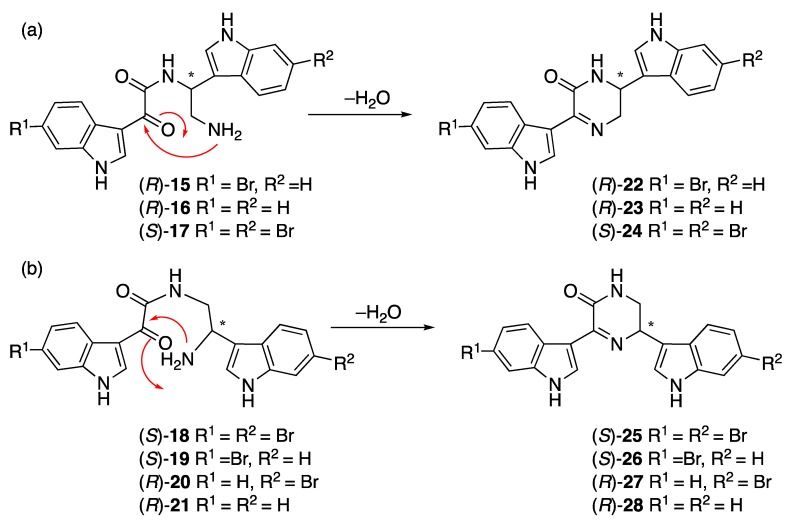
Proposed mechanism for ring closing of **15**–**21** to hamacanthins **a** and **b** derivatives.

Given there are no specific rotation data reported for any compounds related to **15**–**21**, our ORD data could not be compared to any literature values. Therefore, **15**–**21** were passed through a silica SPE column, eluting with dry 20% ethyl acetate in hexane, to facilitate dehydration and ring closure (Scheme 1). The specific rotations of the resulting cyclized compounds **22**–**28** were measured for comparison to the hamacantins (Table 4). The specific rotation of **22** indicated it was (*R*)-6′′-debromohamacanthin A and that **15** must therefore also have an *R* configuration at C-8. Compound **23** had a specific rotation suggesting it was (*R*)-didebromohamacanthin A, the enantiomer of natural (*S*)-bisdebromohamacanthin A. Compound **24** had a specific rotation similar to synthetic and natural (*S*)-hamacanthin A (Table 4) and opposite to synthetic (*R*)-hamacanthin A (−79 deg cm^2^ g^–1^ [19]). The specific rotations of natural and synthetic hamacanthin B (**25**) matched the sign but not the amplitude of the specific rotation for cyclized **18**. Similarly, **28** had a negative specific rotation but much smaller than expected from the literature. In contrast, the specific rotation of **26** was a reasonable match for the published specific rotation of (*S*)-6′′-debromohamacanthin B. Compound **27**, did not match the amplitude and was opposite in sign, suggesting **27** (and **20**) had the opposite configurations to the previously isolated compounds. Kuoko et al. [18] in the total synthesis (+)-hamacanthins A and B, reported the specific rotations of the Boc-protected acyclic amide intermediates as [α]_D_ −2.6, +9.8, and −5.0 deg.cm^2^.g^–1^, which are very small compared to the specific rotations of the cyclic (*S*)-hamacanthins A and B (+83.7 and +170.1 deg.cm^2^.g^–1^, respectively) and generally consistent with the fact that specific rotation is not a useful indicator of absolute configuration in this series of closely related compounds.

**Figure 2:**
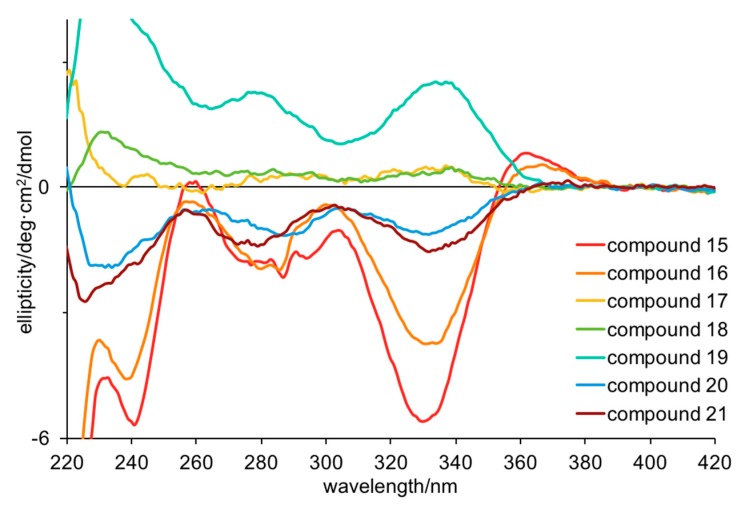
Electronic circular dichroism spectra for compounds **15**–**21** in methanol (50–70 μM).

Turning to electronic circular dichroism (ECD; Figure 2 and Figures S5–S11), it is clear that **15**, **16**, **20**, and **21** have the same configurations, while **17**–**19** have the opposite configurations. The weak ECD for **17**, **18** and **20**, **21** suggests that they are partial racemates, supporting the conclusions above (Table 4). The ECD spectra of **15**–**21** showed negative Cotton effects at 280 and 330 nm and positive Cotton effects at 360 nm for all the compounds except **17**–**19**, which were the opposite. This is consistent with the specific rotations found for **27** and **28** (derived from **20** and **21** respectively) supporting the assignment of **15** and **16** as *R*, and **17**–**19** as *S*. Based on the ECD data, **20** and **21** should have *R* configurations, consistent with a negative specific rotation, but not consistent with the magnitude of the published specific rotations. Taken together, these data suggest that **20** and **21** are partial racemates with the *R* form predominating and that **18** and **19** are partial racemates with the *S* form predominating. This result is intriguing for a number of reasons. Firstly, the one sponge produces enantiomerically pure but opposite configurations of compounds that only differ in the number of bromines, suggesting parallel rather than sequential biosynthesis. Secondly, four compounds are partial racemates, which (if the above is true) suggests these compounds are biosynthesized via two routes in non-equivalent amounts. This type of enantiodivergent biosynthesis has previously been observed in related bromotyrosine and bromopyrrole alkaloids [3,23,24,25]. While it is possible that this chiral center could undergo epimerization during handling, this is unlikely as partial racemization was seen in only four out of the seven compounds isolated from the same extract, and all compounds were handled identically. Finally, our results explain the range of optical rotations published for these compounds. For example, synthetic (*S*)-hamacanthin B (**25**) has been reported with a specific rotation (methanol) of +170–183 deg.cm^2^.g^–1^ [18,22] whereas natural **25** has been reported with +172 deg.cm^2^.g^–1^ [17] or +56 deg.cm^2^.g^–1^ [20]. The latter is close to the value we obtained (+46 deg.cm^2^.g^–1^) for the partial racemate, suggesting that the compound isolated by Bao et al. was also a partial racemate. Enantiodivergent biosynthesis of natural products may be more common than currently recognized.

**Figure 3:**
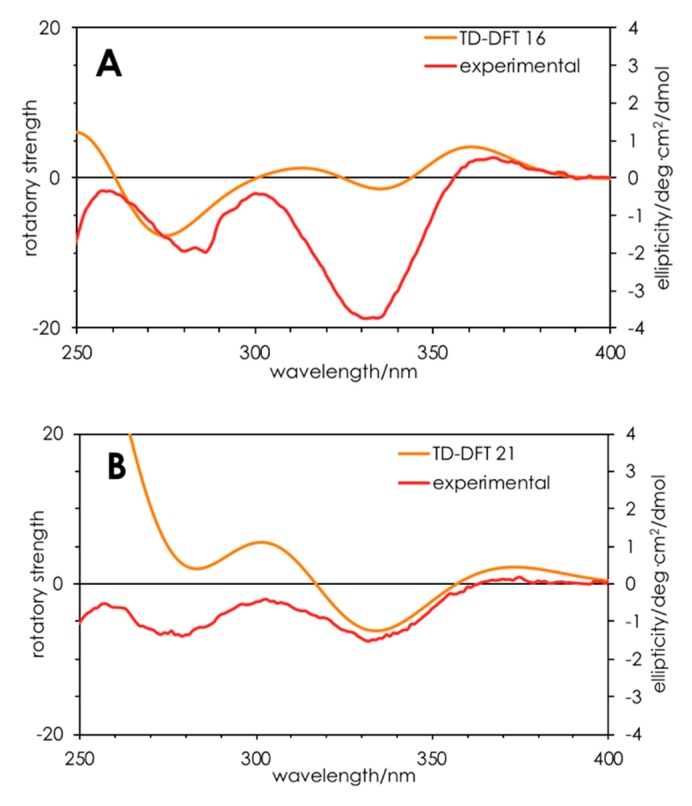
Calculated (orange) and experimental (red) ECD spectra for compounds (*R*)-**16** (**A**) and (*R*)-**21** (**B**). Calculations were performed on ground state-optimized (TDDFT-D3//pbe0/TZVPP) structures in a solvent continuum model (COSMO; methanol).

Finally, turning to time-dependent density functional theory (TDDFT) calculations, we were able to confirm the *R* configuration for **16** and **21**. TDDFT-D3//pbe0/TZVPP-COSMO(methanol) was able to qualitatively reproduce the UV (Figure S12) and ECD (Figure 3) spectra for **16** and **21**. Specifically, the positive Cotton effect at 360 nm and negative Cotton effect at 330 nm were reproduced, albeit with differing oscillator strengths. These results add weight to the interpretation that **17**–**19** have *S* configurations while the others have *R* configurations.

Bisindole alkaloids are a rapidly growing group of sponge metabolites that exhibit potent and diverse bioactivities, including cytotoxicity, antiviral, antifungal, and anti-inflammatory activities [20]. For example, the bisindole alkaloids vinblastine and vincristine have been developed as effective anticancer drugs [26]. Examples of bisindole alkaloids reported from marine sponges include the topsentins [16], which have a ketone and an imidazole moiety as a linker between two indole rings; the nortopsentins [27], which lack the central ketone; and dragmacidins [17] and the hamacanthins (**22**–**28**) [17,18], which have a piperazine–piperazinone linker. Compounds **1**–**9** and **13**–**21** were screened against Methicillin-resistant *Staphylococcus aureus* (MRSA, ATTC 43300), *Escherichia coli* (ATCC 25922), *Klebsiella pneumoniae* (MDR, ATCC 700603), *Acinetobacter baumannii* (ATCC 19606), *Pseudomonas aeruginosa* (ATCC 27853), *Candida albicans* (ATCC 90028), and *Cryptococcus neoformans* var. *grubii* (ATCC 208821). Compounds **2**, **3**, **6**, **13**, and **18** showed some activity against MRSA (Table S10).

## 3. Materials and Methods 

### 3.1. General Experimental Procedures

Specific optical rotations were measured on a Jasco P-1010 Polarimeter (Jasco, Tokyo, Japan). Infrared spectra were recorded on a Nicolet iS10 ATR FTIR Spectrometer (Thermo Scientific, Scorsby, Australia) as thin films. ^1^H NMR and ^13^C NMR spectra were acquired at 25 °C on a Bruker Avance AVII 600 MHz NMR spectrometer in DMSO-*d*_6_, processed using Bruker Topspin v3.2 software and referenced to residual monoprotonated solvent (DMSO-*d*_6_ δ_H_ 2.49; δ_C_ 39.52). LC-MS was performed on an Agilent 1260 Infinity UHPLC coupled to an Agilent 6130B single quadrupole mass spectrometer by electrospray ionization in positive and negative polarity modes. UV–vis spectra were acquired on an Agilent 1260 Infinity diode array detector. High-resolution mass spectra were acquired on a Q Exactive Plus hybrid quadrupole-orbitrap mass spectrometer (Thermo Fisher Scientific, Bremen, Germany) by direct infusion in CH_3_CN. HPLC separations were achieved on a Gilson 506C HPLC system. Analytical and preparative HPLC were performed on Synergi Max-RP C_12_ HPLC columns (Phenomenex, Sydney, Australia): Synergi 10 μm Max-RP 250 × 4.6 mm (1.0 mL/min), Synergi 10 μm Max-RP 250 × 10.0 mm (3.0 mL/min), and Synergi 10 μm Max-RP 250 × 21.2 mm (14.0 mL/min). LC-MS was performed on a C_18_ analytical column (Phenomenex Gemini 3 μm C_18_, 150 × 2.0 mm) with a gradient from 5%–95% CH_3_CN in H_2_O with 0.025% formic acid, flow rate 0.2 mL/min, and UV detection at 254 nm.

### 3.2. Biological Material

The sponge was collected from the Western Continental Slope (Station J954), Northland, New Zealand, (34.6333° S, 172.2250° E) at a depth of ~200 m, by gear rock dredge in June 1981. The sample was frozen upon collection, freeze-dried and stored at room temperature. It was identified as *Lamellomorpha strongylata* Bergquist, 1968 (class Demospongiae, order Tetractinellida, suborder Astrophorina, family Vulcanellidae), and deposited at the New Zealand National Institute of Water and Atmospheric Research (NIWA) under the accession number NIWA 92907.

### 3.3. Extraction and Isolation

The freeze-dried sponge (150 g) was blended to a powder and extracted sequentially with hexane (3 × 500 mL), ethyl acetate (3 × 500 mL), and *n*-butanol (2 × 200 mL) to yield 1.2, 1.2, and 0.6 g of crude extracts, respectively. The ethyl acetate extract was subjected to gel permeation chromatography (Sephadex LH-20; 22 × 400 mm; 1:1 CHCl_3_:MeOH), to afford seven subfractions (E1–E7), which were subjected to LC-MS analysis. Fractions E3–E5 and E7 were individually passed through an Alltech high capacity C_18_ solid-phase extraction cartridge, eluting with 90% CH_3_CN in H_2_O, and the eluates were purified separately by preparative HPLC columns (Synergi Max-RP C_12_, 10 μm, 250 × 22 mm) using a gradient from 20%–95% CH_3_CN in H_2_O with 0.01% TFA. Fraction E3 (120.8 mg) afforded **1** (*t*_R_ 33.2 min; 0.5 mg), **3** (*t*_R_ 22.5 min; 0.5 mg), **4** (*t*_R_ 25.5 min; 0.9 mg), **5** (*t*_R_ 26.0 min; 2.2 mg), **6** (*t*_R_ 31.0 min; 1.7 mg), **7** (*t*_R_ 28.5 min; 3.2 mg), **15** (*t*_R_ 13.5 min; 10.5 mg), and **16** (*t*_R_ 10.2 min; 5.5 mg). Fraction E4 (78.9 mg) afforded **8** (*t*_R_ 30.0 min; 8.5 mg), **13** (*t*_R_ 21.0 min; 1.9 mg), **14** (*t*_R_ 19.8 min; 1.9 mg), **18** (*t*_R_ 23.5 min; 2.0 mg), **19** (*t*_R_ 15.5 min; 1.9 mg), and **20** (*t*_R_ 18.0 min; 5.2 mg). Fraction E5 (111.5 mg) afforded **2** (*t*_R_ 22.4 min; 0.5 mg). Fraction E6 (282.7 mg) did not require further purification and was identified as **9** by NMR spectroscopy. Fraction E7 (128.6 mg) afforded **10** (*t*_R_ 19.6 min; 6.2 mg), **11** (*t*_R_ 17.4 min; 2.4 mg), and **12** (*t*_R_ 21.2 min; 5.8 mg). The *n*-butanol extract was also subjected to gel permeation chromatography (Sephadex LH-20; MeOH) to afford six subfractions (B1–B6), which were subjected to LC-MS analysis. Fractions B5 and B6 were individually passed through a C_18_ Sep-Pak and eluted with 90% CH_3_CN in H_2_O. The eluates were purified by HPLC (Synergi Max-RP C_12_, 10 μm, 250 × 22 mm) using gradients from 20% CH_3_CN to 95% CH_3_CN in H_2_O with 0.01% TFA. Fraction B5 (45.6 mg) afforded **15** (*t*_R_ 14.0 min; 19.1 mg) and **16** (*t*_R_ 10.2 min; 14.9 mg) and fraction B6 (38.2 mg) afforded **17** (*t*_R_ 18.0 min; 1.7 mg) and **21** (*t*_R_ 10.5 min; 1.7 mg). Please refer to Figures S13–S75 for NMR spectra of all isolated compounds.

**(*Z*)-Coscinamide D (1):** yellow amorphous powder (0.5 mg); UV (MeOH) λ_max_ 214, 262, 278, 340 nm; IR (neat film) ν_max_ 3437, 3195, 2987, 2939, 1647, 1594, 1541, 1489, 1236, 1130, 924, 738 cm^−1^; ^1^H and ^13^C NMR data, see Table 1, Table S1; ESIMS *m/z* 408/410 [M + H]^+^ isotopic cluster in the ratio 1:1; HRESIMS *m/z* 430.0156 [M + Na]^+^ (calcd for C_20_H_14_^79^BrN_3_O_2_Na^+^, 430.0162).

**(*E*)-Coscinamide D (2):** yellow amorphous powder (0.5 mg); UV (MeOH) λ_max_ 214, 260, 276, 342 nm; IR (neat film) ν_max_ 3304, 2976, 1674, 1616, 1482, 1438, 1204, 1130, 801, 744 cm^−1^; ^1^H and ^13^C NMR data, see Table 1, Table S2; ESIMS *m/z* 408/410 [M + H]^+^ isotopic cluster in the ratio 1:1; HRESIMS *m*/*z* 406.0199 [M − H]^−^ (calcd for C_20_H_13_^79^BrN_3_O_2_^−^, 406.0197).

**Lamellomorphamide A (3):** yellow amorphous powder (1.1 mg); UV (MeOH) λ_max_ 208, 258, 302 nm; IR (neat film) ν_max_ 3198, 2978, 1670, 1616, 1490, 1424, 1238, 1131, 742 cm^−1^; ^1^H and ^13^C NMR data, see Table 2, Table S3; ESIMS *m/z* 346 [M + H]^+^; HRESIMS *m*/z 346.1183 [M + H]^+^ (calcd for C_20_H_16_N_3_O_3_^+^, 346.1186).

**Lamellomorphamide B (4):** yellow amorphous powder (0.9 mg); UV (MeOH) λ_max_ 208, 246, 268, 310 nm; IR (neat film) ν_max_ 3269, 2977, 1675, 1635, 1616, 1489, 1436, 1202, 1131, 801, 746 cm^−1^; ^1^H and ^13^C NMR data, see Table 2, Table S4; ESIMS *m/z* 424/426 [M + H]^+^ isotopic cluster in the ratio 1:1; HRESIMS *m*/*z* 446.0108 [M + Na]^+^ (calcd for C_20_H_14_^79^BrN_3_O_3_Na^+^, 446.0111).

**Lamellomorphamide C (5):** yellow amorphous powder (2.2 mg); UV (MeOH) λ_max_ 212, 260, 302 nm; IR (neat film) ν_max_ 3349, 2926, 1676, 1634, 1496, 1435, 1202, 1131, 801, 722 cm^−1^; ^1^H and ^13^C NMR data, see Table 3, Table S5; ESIMS *m/z* 424/426 [M + H]^+^ isotopic cluster in the ratio 1:1; HRESIMS *m*/*z* 446.0108 [M + Na]^+^ (calcd for C_20_H_14_^79^BrN_3_O_3_Na^+^, 446.0111).

**Lamellomorphamide D (6):** yellow amorphous powder (1.7 mg); UV (MeOH) λ_max_ 214, 273, 310 nm; IR (neat film) ν_max_ 3291, 2923, 1682, 1615, 1496, 1416, 1202, 1131, 802 cm^−1^; ^1^H and ^13^C NMR data, see Table 3, Table S6; ESIMS *m/z* 502/504/506 [M + H]^+^ isotopic cluster in the ratio 1:2:1; HRESIMS *m*/*z* 501.9396 [M + H]^+^ (calcd for C_20_H_14_^79^Br_2_N_3_O_3_^+^, 501.9396).

### 3.4. Biological Assays

Primary antimicrobial screening was carried out by whole-cell growth inhibition assays, using the samples at 10 and 20 μM for *S. aureus* only in DMSO in duplicate (Table S10). The inhibition of growth was measured against methicillin-resistant *S. aureus* (ATTC 43300), *E. coli* (ATCC, 25922), *K. pneumoniae* (MDR, ATCC 700603), *A. baumannii* (ATCC 19606), *P. aeruginosa* (ATCC 27853), *C. albicans* (ATCC 90028), and *C. neoformans* var. *grubii* (ATCC 208821). Bacteria were cultured in cation-adjusted Mueller Hinton broth (CAMHB) at 37 °C overnight, and the culture diluted 40-fold with fresh broth and incubated at 37 °C for 1.5–3 h. The resultant mid-log phase cultures were diluted (CFU/mL measured by OD_600_), then added to each well of the compound containing plates, giving a cell density of 5 × 10^5^ CFU/mL and a total volume of 50 μL. All the plates were covered and incubated at 37 °C for 18 h without shaking. Inhibition of bacterial growth was determined by measuring optical density at 600 nm (OD_600_), and the percentage of growth inhibition was calculated for each well, using the negative control (media only) and positive control (bacteria without inhibitors) on the same plate as references. Fungi strains were cultured for 3 days on yeast extract–peptone dextrose (YPD) agar at 30 °C. A yeast suspension of 1 × 10^6^ to 5 × 10^6^ CFU/mL (as determined by OD_530_) was prepared from five colonies. The suspension was subsequently diluted and added to each well of the compound containing plates giving a final cell density of fungi suspension of 2.5 × 10^3^ CFU/mL and a total volume of 50 μL. All plates were covered and incubated at 35 °C for 24 h without shaking. Growth inhibition of *C. albicans* was determined by measuring absorbance at 530 nm (OD_530_), while the growth inhibition of *C. neoformans* was determined by measuring the difference in optical density between 600 and 570 nm (OD_600–570_), after the addition of resazurin (0.001% final concentration) and incubated at 35 °C for additional 2 h. The absorbance was measured using a Biotek Synergy HTX plate reader. The percentage of growth inhibition was calculated for each well, using the negative control (media only) and positive control (fungi without inhibitors) on the same plate. The significance of the inhibition values was determined by modified Z-scores. Samples with inhibition value above 80% and Z-scores above 2.5 for either replicate were classed as actives. Samples with inhibition values between 50%–80% and Z-scores above 2.5 for either replicate were classed as partial actives. All screening was performed in duplicate using the same microbial cultures, but with replicates on different plates. In addition, two values were used as quality control for individual plates and standard antibiotic controls at different concentrations (>MIC and <MIC). Colistin and vancomycin were used as positive bacterial inhibitor standards for Gram-negative and Gram-positive bacteria, respectively. Fluconazole was used as a positive fungal inhibitor standard for *C. albicans* and *C. neoformans*. The plate passes the quality control if the Z′-factor > 0.4 and the standards are active and inactive at highest and lowest concentrations, respectively.

## 4. Conclusions

In summary, two new bisindole alkaloids, (*Z*)-coscinamide D and (*E*)-coscinamide D, together with lamellomorphamides A–D were isolated for the first time from a natural source. While bisindole alkaloids have been reported from *Rhaphisia lacazei*, *Spongosorites*, *Hamacantha* and *Coscinoderma* spp, it is of interest to note that this is the first time this class of compound has been isolated from the genus *Lamellomorpha*, for which there are only two previous reports of unrelated natural products. In addition, we have isolated seven hamacanthin-type alkaloids, three of which appear to be optically pure while the other four are partial racemates, providing another fascinating example of enantiodivergent biosynthesis in marine alkaloids.

## Figures and Tables

**Table 1:** NMR Data (DMSO-*d*_6_; ^1^H 600 MHz, ^13^C 150 MHz) for (*Z*)-coscinamide D (**1**) and (*E*)–coscinamide D (**2**).

	(Z)-Coscinamide D (1)		(E)-Coscinamide D (2)	
Position ^a^	δ_C_, type	δ_H_ (*J* in Hz)	δ_C_, type	δ_H_ (*J* in Hz)
**1-NH**		11.45, s		11.21, s
**2**	123.8, CH	7.64, m	124.4, CH	7.49, d (2.4)
**3**	109.6, C		111.6, C	
**3a**	126.4, C		124.8, C	
**4**	111.7, CH	7.43, d (8.1)	119.0, CH	7.68, d (7.8)
**5**	122.0, CH	7.16, td (7.4, 1.0)	119.5, CH	7.09, t (7.4)
**6**	119.4, CH	7.07, td (7.4, 1.0)	121.6, CH	7.14, t (7.4)
**7**	118.4, CH	7.63, m	111.9, CH	7.39, m
**7a**	135.8, C		136.9, C	
**8**	106.1, CH	6.24, d (9.2)	110.2, CH	6.85, d (14.7)
**9**	117.2, CH	6.80, dd (11.0, 9.2)	118.5, CH	7.40, m
**10-NH**		9.66, d (11.0)		10.85, d (9.9)
**1′-NH**		12.43, br s		12.36, br s
**2′**	139.9, CH	8.92, d (3.2)	139.4, CH	8.85, d (3.2)
**3′**	111.8, C		112.2, C	
**3a′**	125.3, C		125.3, C	
**4′**	123.0, CH	8.16, d (8.4)	122.9, CH	8.19, d (8.5)
**5′**	125.6, CH	7.41, dd (8.4, 1.8)	125.5, CH	7.43, m
**6′**	116.1, C		116.0, C	7.75, d (1.8)
**7′**	115.4, CH	7.76, d (1.8)	115.4, CH	
**7a′**	137.2, C		137.3, C	
**8′**	180.0, C		181.3, C	
**9′**	159.7, C		160.0, C	

^a^ see Figure 1 for locant numbering.

**Table 2:** NMR Data (DMSO-*d*_6_; ^1^H 600 MHz, ^13^C 150 MHz) for lamellomorphamides A (**3**) and B (**4**).

	Lamellomorphamide A (3)		Lamellomorphamide B (4)	
Position ^a^	δ_C_, type	δ_H_ (*J* in Hz)	δ_C_, type	δ_H_ (*J* in Hz)
**1-NH**		12.07, br s		12.17, br s
**2**	133.8, CH	8.50, d (3.2)	134.7, CH	8.53, d (3.0)
**3**	113.9, C		113.9, C	
**3a**	125.4, C		124.5, C	
**4**	121.1, CH	8.16, m	122.7, CH	8.09, d (8.5)
**5**	121.9, CH	7.20, td (7.1, 1.1)	124.9, CH	7.35, dd (8.5, 1.8)
**6**	122.6, CH	7.23, td (7.1, 1.1)	115.0, C	7.69, d (1.8)
**7**	112.6, CH	7.49, m	115.6, CH	
**7a**	136.3, C		137.4, C	
**8**	189.2 C		189.4, C	
**9**	45.7, CH_2_	4.63, d (5.9)	45.7, CH_2_	4.62, d (6.0)
**10-NH**		8.91, t (5.9)		8.93, t (5.3)
**1′-NH**		12.26, br s		12.25, br s
**2′**	138.7, CH	8.82, d (3.2)	138.6, CH	8.80, d (3.3)
**3′**	112.3, C		112.3, C	
**3a′**	126.2, C		126.2, C	
**4′**	121.3, CH	8.26, m	121.3, CH	8.25, m
**5′**	122.9, CH	7.27, m	122.9, CH	7.27, m
**6′**	123.5, CH	7.28, m	123.6, CH	7.28, m
**7′**	112.2, CH	7.54, m	112.7, CH	7.53, m
**7a′**	136.4, C		136.3, C	
**8′**	181.9, C		181.8, C	
**9′**	163.8, C		163.8, C	

^a^ see Figure 1 for locant numbering.

**Table 3:** NMR Data (DMSO-*d*_6_; ^1^H 600 MHz, ^13^C 150 MHz) for lamellomorphamides C (**5**) and D (**6**).

	Lamellomorphamide C (5)		Lamellomorphamide D (6)	
Position ^a^	δ_C_, type	δ_H_ (*J* in Hz)	δ_C_, type	δ_H_ (*J* in Hz)
**1-NH**		12.07, br s		12.17, br s
**2**	133.8, CH	8.50, d (3.2)	134.6, CH	8.53, d (3.1)
**3**	113.9, C		113.9, C	
**3a**	125.2, C		124.4, C	
**4**	121.1, CH	8.16, m	122.8, CH	8.09, d (8.5)
**5**	121.9, CH	7.20, td (7.1, 1.1)	124.9, CH	7.35, dd (8.5, 1.8)
**6**	122.9, CH	7.23, td (7.1, 1.1)	115.5, C	
**7**	112.2, CH	7.49, m	114.9, CH	7.69, d (1.8)
**7a**	136.4, C		137.2, C	
**8**	189.1 C		189.1, C	
**9**	45.7, CH_2_	4.63, d (5.9)	45.7, CH_2_	4.62, d (5.9)
**10-NH**		8.94, t (5.9)		8.93, t (5.9)
**1′-NH**		12.32, br s		12.25, br s
**2′**	139.4, CH	8.83, d (3.2)	139.4, CH	8.81, d (3.2)
**3′**	112.2, C		112.2, C	
**3a′**	125.3, C		125.2, C	
**4′**	123.0, CH	8.18, d (8.4)	123.0, CH	8.18, d (8.4)
**5′**	125.5, CH	7.42, dd (8.4, 1.8)	125.5, CH	7.42, dd (8.4, 1.8)
**6′**	116.0, C		116.0, C	
**7′**	115.3, CH	7.75, d (1.8)	115.4, CH	7.75, d (1.8)
**7a′**	137.2, C		137.3, C	
**8′**	181.9, C		181.9, C	
**9′**	163.4, C		163.5, C	
				

^a^ see Figure 1 for locant numbering.

**Table 4:** Specific rotation comparison of cyclized compounds **22**–**28**.

Compound	Measured Specific Rotation	Literature Values
	(deg.cm^2^.g^–1^)	(deg.cm^2^.g^–1^)
**22**	−82	−76 [20]
**23**	−34	+59 [14]
**24**	64	+82, [21] +84 [19]
**25**	46	+172, [17] +183 [22]
**26**	36	+43 [20]
**27**	38	−194 [14]
**28**	−36	−288 [14]

## Supplementary Materials

The following are available online at https://www.mdpi.com/1660-3397/17/12/683/s1. Supplementary Materials including NMR spectra, tabulated 2D NMR data, HRMS, 1D and 2D NMR spectra, CD spectra.
