# Supplementary material for: Bisindole Alkaloids from a New Zealand Deep-Sea Marine Sponge Lamellomorpha strongylata
Source: Mar Drugs. 2019 Dec 4;17(12):683. doi: 10.3390/md17120683 (PMC6950519; doi:10.3390/md17120683)
Supplement: Supplementary file 1 [file marinedrugs-17-00683-s001.pdf]

SUPPORTING INFORMATION

Bisindole Alkaloids from a New Zealand Deep Sea Marine Sponge *Lamellomorpha strongylata*

*Kavita Ragini, Andrew M. Piggott and Peter Karuso**

Department of Chemistry and Biomolecular Sciences, Macquarie University, Sydney, NSW 2109, Australia.

Table of Contents

Characterization of Compounds ... 3
Table S1. NMR data (600 MHz) for (*Z*)-coscinamide D (**1**) in DMSO-d_6 ... 6
Table S2. NMR data (600 MHz) for (*E*)-coscinamide D (**2**) in DMSO-d_6 ... 7
Table S3. NMR data (600 MHz) for Lamellomorphamide A (**3**) in DMSO-d_6 ... 8
Table S4. NMR data (600 MHz) for Lamellomorphamide B (**4**) in DMSO-d_6 ... 9
Table S5. NMR data (600 MHz) for Lamellomorphamide C (**5**) in DMSO-d_6 ... 10
Table S6. NMR data (600 MHz) for Lamellomorphamide D (**6**) in DMSO-d_6 ... 11
Table S7. NMR data (600 MHz) for **15**-**17** in DMSO-d_6 ... 12
Table S8. NMR data (600 MHz) for **18** and **20** in DMSO-d_6 ... 13
Table S9. NMR data (600 MHz) for **19** and **21** in DMSO-d_6 ... 14
Table S10. Bioassay results of compounds **1-9**, **13-21** ... 15

Figure S1. HPLC chromatogram of compound **15** ... 16
Figure S2. UV-Vis and Mass spectra of peaks eluting in Figure S1. ... 17
Figure S3. HPLC chromatogram of *n*-butanol fraction with UV detection at 254 nm ... 18
Figure S4. TIC and EIC of positive ion scan of *n*-butanol fraction ... 18
Figure S5. ECD and UV spectra of **15** (*c* 0.06 mM, MeOH) ... 19
Figure S6. ECD and UV spectra of **16** (*c* 0.07 mM, MeOH) ... 19
Figure S7. ECD and UV spectra of **17** (*c* 0.05 mM, MeOH) ... 20
Figure S8. ECD and UV spectra of **18** (*c* 0.05 mM, MeOH) ... 20
Figure S9. ECD and UV spectra of **19** (*c* 0.12 mM, MeOH) ... 21
Figure S10. ECD and UV spectra of **20** (*c* 0.06 mM, MeOH) ... 21
Figure S11. ECD and UV spectra of **21** (*c* 0.07 mM, MeOH) ... 22
Figure S12. UV spectrum of **16** (*c* 0.07 mM, MeOH) compared to the calculated spectrum. ... 22
Figure S13. ^{1}H NMR spectrum (600 MHz) of (*Z*)-coscinamide D (**1**) in DMSO-d_6 ... 23
Figure S14. ^{13}C NMR spectrum (150 MHz) of (*Z*)-coscinamide D (**1**) in DMSO-d_6 ... 24
Figure S15. ^{1}H-^{13}C HSQC spectrum (600 MHz) of (*Z*)-coscinamide D (**1**) in DMSO-d_6 ... 25
Figure S16. ^{1}H-^{13}C HMBC spectrum (600 MHz) of (*Z*)-coscinamide D (**1**) in DMSO-d_6 ... 25
Figure S17. ^{1}H-^{1}H COSY spectrum (600 MHz) of (*Z*)-coscinamide D (**1**) in DMSO-d_6 ... 26
Figure S18. ^{1}H-^{1}H ROESY spectrum (600 MHz) of (*Z*)-coscinamide D (**1**) in DMSO-d_6 ... 26
Figure S19. ^{1}H NMR spectrum (600 MHz) of coscinamide D (**2**) in DMSO-d_6 ... 27
Figure S20. ^{13}C NMR spectrum (150 MHz) of coscinamide D (**2**) in DMSO-d_6 ... 28
Figure S21. ^{1}H-^{13}C HSQC spectrum (600 MHz) of coscinamide D (**2**) in DMSO-d_6 ... 29
Figure S22. ^{1}H-^{13}C HMBC spectrum (600 MHz) of coscinamide D (**2**) in DMSO-d_6 ... 29
Figure S23. ^{1}H-^{1}H COSY spectrum (600 MHz) of coscinamide D (**2**) in DMSO-d_6 ... 30
Figure S24. ^{1}H-^{1}H ROESY spectrum (600 MHz) of coscinamide D (**2**) in DMSO-*d* ... 30
Figure S25. ^{1}H NMR spectrum (600 MHz) of lamellomorphamide A (**3**) in DMSO-d_6 ... 31
Figure S26. ^{13}C NMR spectrum (150 MHz) of lamellomorphamide A (**3**) in DMSO-d_6 ... 32
Figure S27. ^{1}H- ^{13}C HSQC spectrum (600 MHz) of lamellomorphamide A (**3**) in DMSO-d_6 ... 33
Figure S28. ^{1}H- ^{13}C HMBC spectrum (600 MHz) of lamellomorphamide A (**3**) in DMSO-d_6 ... 33
Figure S29. ^{1}H- ^{1}H COSY spectrum (600 MHz) of lamellomorphamide A (**3**) in DMSO-d_6 ... 34

Figure S30. ^{1}H- ^{1}H ROESY spectrum (600 MHz) of lamellomorphamide A (**3**) in DMSO- 34
Figure S31. ^{1}H NMR spectrum (600 MHz) of lamellomorphamide B (**4**) in DMSO-*d*$_6$ 35
Figure S32. ^{13}C NMR spectrum (150 MHz) of lamellomorphamide B (**4**) in DMSO-*d*$_6$ 36
Figure S33. ^{1}H- ^{13}C HSQC spectrum (600 MHz) of lamellomorphamide B (**4**) in DMSO-*d*$_6$ 37
Figure S34. ^{1}H- ^{13}C HMBC spectrum (600 MHz) of lamellomorphamide B (**4**) in DMSO-*d*$_6$ 37
Figure S35. ^{1}H- ^{1}H COSY spectrum (600 MHz) of lamellomorphamide B (**4**) in DMSO-*d*$_6$ 38
Figure S36. ^{1}H- ^{1}H ROESY spectrum (600 MHz) of lamellomorphamide B (**4**) in DMSO-*d*$_6$ 38
Figure S37. ^{1}H NMR spectrum (600 MHz) of lamellomorphamide C (**5**) in DMSO-*d*$_6$ 39
Figure S38. ^{13}C NMR spectrum (150 MHz) of lamellomorphamide C (**5**) in DMSO-*d*$_6$ 40
Figure S39. ^{1}H- ^{13}C HSQC spectrum (600 MHz) of lamellomorphamide C (**5**) in DMSO-*d*$_6$ 41
Figure S40. ^{1}H- ^{13}C HMBC spectrum (600 MHz) of lamellomorphamide C (**5**) in DMSO-*d*$_6$ 41
Figure S41. ^{1}H- ^{1}H COSY spectrum (600 MHz) of lamellomorphamide C (**5**) in DMSO-*d*$_6$ 42
Figure S42. ^{1}H- ^{1}H ROESY spectrum (600 MHz) of lamellomorphamide C (**5**) in DMSO-*d* 42
Figure S43. ^{1}H NMR spectrum (600 MHz) of lamellomorphamide D (**6**) in DMSO-*d*$_6$ 43
Figure S44. ^{13}C NMR spectrum (150 MHz) of lamellomorphamide D (**6**) in DMSO-*d*$_6$ 44
Figure S45. ^{1}H- ^{13}C HSQC spectrum (600 MHz) of lamellomorphamide D (**6**) in DMSO-*d*$_6$ 45
Figure S46. ^{1}H- ^{13}C HMBC spectrum (600 MHz) of lamellomorphamide D (**6**) in DMSO-*d*$_6$ 45
Figure S47. ^{1}H- ^{1}H COSY spectrum (600 MHz) of lamellomorphamide D (**6**) in DMSO-*d*$_6$ 46
Figure S48. ^{1}H- ^{1}H ROESY spectrum (600 MHz) of lamellomorphamide D (**6**) in DMSO-*d*$_6$ 46
Figure S49. ^{1}H NMR spectrum (600 MHz) of coscinamide B (**7**) in DMSO-*d*$_6$ 47
Figure S50. ^{1}H NMR spectrum (600 MHz) of coscinamide B (**7**) in DMSO-*d*$_6$ 48
Figure S51. ^{1}H NMR spectrum (600 MHz) of (*Z*)-coscinamide B (**8**) in DMSO-*d*$_6$ 49
Figure S52. ^{13}C NMR spectrum (150 MHz) of (*Z*)-coscinamide B (**8**) in DMSO-*d*$_6$ 50
Figure S53. ^{1}H NMR spectrum (600 MHz) of deoxytopsentin (**9**) in DMSO-*d*$_6$ 51
Figure S54. ^{1}H NMR spectrum (600 MHz) of deoxytopsentin (**9**) in methanol-*d*$_4$ 52
Figure S55. ^{1}H NMR spectrum (600 MHz) of isobromodeoxytopsentin (**10**) in DMSO-*d*$_6$ 53
Figure S56. ^{1}H NMR spectrum (600 MHz) of bromodeoxytopsentin (**11**) in DMSO-*d*$_6$ 54
Figure S57. ^{1}H NMR spectrum (600 MHz) of dibromodeoxytopsentin (**12**) in DMSO-*d*$_6$ 55
Figure S58. ^{1}H NMR spectrum (600 MHz) of 6-bromoindole-3-carboxylic acid (**13**) in DMSO-*d*$_6$ 56
Figure S59. ^{13}C NMR spectrum (150 MHz) of 6-bromoindole-3-carboxylic acid (**13**) in DMSO-*d*$_6$ 57
Figure S60. ^{1}H NMR spectrum (600 MHz) of (6-bromo-1*H*-indol-3-yl) oxoacetamide (**14**) in DMSO-*d*$_6$ 58
Figure S61. ^{13}C NMR spectrum (150 MHz) of (6-bromo-1*H*-indol-3-yl) oxoacetamide (**14**) in DMSO-*d*$_6$ 59
Figure S62. ^{1}H NMR spectrum (600 MHz) of 3,4-seco-6″-debromohamacanthin A (**15**) in DMSO-*d*$_6$ 60
Figure S63. ^{13}C NMR spectrum (150 MHz) of 3,4-seco-6″-debromohamacanthin A (**15**) in DMSO-*d*$_6$ 61
Figure S64. ^{1}H NMR spectrum (600 MHz) of 3,4-seco-6′,6″-didebromohamacanthin A (**16**) in DMSO-*d*$_6$ 62
Figure S65. ^{13}C NMR spectrum (150 MHz) of 3,4-seco-6′,6″-didebromohamacanthin A (**16**) in DMSO-*d*$_6$ 63
Figure S66. ^{1}H NMR spectrum (600 MHz) of 3,4-seco-hamacanthin A (**17**) in DMSO-*d*$_6$ 64
Figure S67. ^{13}C NMR spectrum (150 MHz) of 3,4-seco-hamacanthin A (**17**) in DMSO-*d*$_6$ 65
Figure S68. ^{1}H NMR spectrum (600 MHz) of 3,4-seco-hamacanthin B (**18**) in DMSO-*d*$_6$ 66
Figure S69. ^{13}C NMR spectrum (150 MHz) of 3,4-seco-hamacanthin B (**18**) in DMSO-*d*$_6$ 67
Figure S70. ^{1}H NMR spectrum (600 MHz) of 3,4-seco-6″-debromohamacanthin B (**19**) in DMSO-*d*$_6$ 68
Figure S71. ^{13}C NMR spectrum (150 MHz) of 3,4-seco-6″-debromohamacanthin B (**19**) in DMSO-*d*$_6$ 69
Figure S72. ^{1}H NMR spectrum (600 MHz) of 3,4-seco-6′-debromohamacanthin B (**20**) in DMSO-*d*$_6$ 70
Figure S73. ^{13}C NMR spectrum (150 MHz) of 3,4-seco-6′-debromohamacanthin B (**20**) in DMSO-*d*$_6$ 71
Figure S74. ^{1}H NMR spectrum (600 MHz) of 3,4-seco-6′,6″-didebromohamacanthin B (**21**) in DMSO-*d*$_6$ 72
Figure S75. ^{13}C NMR spectrum (150 MHz) of 3,4-seco-6′,6″-didebromohamacanthin B (**21**) in DMSO-*d*$_6$ 73
References 74

Characterization of Compounds

Coscinamide B (**7**):[1] orange amorphous powder (3.2 mg); UV (MeOH) λ_{max} 208, 268, 346 nm; IR (neat film) ν_{max} 3437, 3195, 2987, 2939, 1647, 1594, 1541, 1489, 1236, 1130, 924, 738 cm^{-1}; ^{1}H NMR (DMSO-d_6, 600MHz) δ 12.30 (d, *J* = 2.4 Hz, NH), 11.20 (br s, *J* = 2.0 Hz, NH), 10.83 (d, *J* = 10.0 Hz, H-10), 8.83 (d, *J* = 3.3 Hz, H-2′), 8.28 (m, H-4′), 7.68 (d, *J* = 7.9 Hz, H-4), 7.55 (m, H-7′), 7.49 (d, *J* = 2.4 Hz, H-2), 7.42 (m, *J* = 10 Hz, H-9), 7.39 (m, *J* = 7.3 Hz, H-7), 7.28 (m, H-6′), 7.27 (m, H-5′), 7.14 (dt, *J* = 7.3 Hz, H-6), 7.10 (dt, *J* = 7.8 Hz, H-5), 6.84 ((d, *J* = 14.8 Hz, H-8). ^{13}C-NMR (DMSO-d_6, 150MHz) δ 181.2 (C-8′), 160.4 (C-9′), 138.7 (C-2′), 136.9 (C-7a), 136.3 (C-7a′), 126.2 (C-3a′), 124.8 (C-3a), 124.4 (C-2), 123.6 (C-6′), 122.7 (C-5′), 121.6 (C-4′), 121.3 (C-6), 119.5 (C-5), 119.1 (C-4), 118.6 (C-9), 112.6 (C-7′), 112.3(C-3′) 112.0 (C-3), 111.6 (C-7) 110.0 (C-8); Mass spectrum (ESI+) *m/z*: 330 $[M + H]^+$ for $C_{20}H_{15}N_3O_2$.

(*Z*)-Coscinamide B (**8**):[2] yellow amorphous powder (8.5 mg); UV (MeOH) λ_{max} 208, 260, 265, 348 nm; IR (neat film) ν_{max} 3265, 2976, 1675, 1622, 1438, 1417, 1203, 1132, 995, 801, 746 cm^{-1}; ^{1}H NMR (DMSO-d_6, 600MHz) δ 12.37 (br s, NH), 11.45 (br s, NH), 9.68 (d, *J* = 11.3 Hz, H-10), 8.92 (d, *J* = 3.3 Hz, H-2′), 8.23 (m, *J* = 6.5, 1.9 Hz H-4′), 7.64 (m, H-2), 7.63 (m, H-7), 7.55 ((m, H-7′), 7.44 (td, *J* = 8.1 Hz, H-4), 7.28 (m, H-6′), 7.27 (m, H-5′), 7.16 (m, H-5), 7.07 (m, H-6), 6.81 (dd, *J* = 9.2, 11.3 Hz, H-9), 6.23 (d, *J* = 9.2 Hz, H-8); ^{13}C-NMR (DMSO-d_6, 150MHz) δ 179.8 (C-8′), 159.8 (C-9′), 139.2 (C-2′), 136.3 (C-7a′), 135.8 (C-7a), 126.4 (C-3a), 126.2 (C-3a′), 123.7 (C-2), 123.7 (C-6′), 122.8 (C-5′), 122.0 (C-5), 121.4 (C-4′), 119.4 (C-6′), 118.4 (C-7), 117.3 (C-9), 112.7 (C-7′), 111.9(C-3′) 111.7 (C-4), 109.7 (C-3), 105.9 (C-8); Mass spectrum (ESI+) *m/z*: 330 $[M + H]^+$ for $C_{20}H_{15}N_3O_2$.

Deoxytopsentin (**9**):[3] yellow amorphous powder (282.7 mg); UV (MeOH) λ_{max} 208, 252, 274, 375 nm; IR (neat film) ν_{max} 3363, 3263, 1682, 1627, 1522, 1415, 1239, 1104, 855, 738 cm^{-1}; ^{1}H NMR (MeOD, 600MHz) δ 9.01 (s, H-2′), 8.40 (m, H-4′), 7.94 (d, *J* = 7.5 Hz, H-4), 7.77 (s, H-2), 7.57 (s, H-4″), 7.52 (m, H-7′), 7.28 (m, H-6′), 7.26 (m, H-5′), 7.46 (d, *J* = 7.5 Hz, H-7), 7.21 (dt, *J* = 6.9, 1.2 Hz, H-6), 7.17 (dt, *J* = 6.9, 1.2 Hz, H-5); Mass spectrum (ESI+) *m/z*: 327 $[M + H]^+$; HRESIMS *m*/z 327.1237 $[M + H]^+$ (calcd. for $C_{20}H_{14}N_4O$, 327.1168)

Isobromodeoxytopsentin (**10**):[4] yellow amorphous powder (6.2 mg); UV (MeOH) λ_{max} 213, 254, 280, 372 nm; ^{1}H NMR (DMSO-d_6, 600MHz) ***10a*** δ 13.25 (br s, H-1″), 12.17 (s, H-1′), 11.45 (br s, H-1), 9.38 (br s, H-2′), 8.33 (s, H-4′), 8.16 (d, *J* = 8.0 Hz, H-4), 8.11 (d, *J* = 2.4 Hz, H-2), 7.75 (d, *J* = 1.6 Hz, H-7′), 7.70 (br s, H-4″), 7.45 (d, 8.0 Hz, H-7), 7.39 (m, H-5′), 7.18 (m, H-6), 7.13 (m, H-5); ***10b*** δ 13.17 (br s, H-1″), 12.11 (s, H-1′), 11.23 (br s, H-1), 9.18 (br s, H-2′), 8.31 (s, H-4′), 7.91 (d, *J* = 8.0 Hz, H-4), 7.84 (d, *J* = 2.4 Hz, H-2), 7.74 (d, *J* = 1.6 Hz, H-7′), 7.63 (br s, H-4″), 7.43 (m, H-7), 7.38 (m, H-5′), 7.16 (d, *J* = 8.0 Hz, H-6), 7.13 (t, *J* = 7.2 Hz, H-5); Mass spectrum (ESI+) *m/z*: isotopic cluster 405:407 (in ratio 1:1) $[M + H]^+$ for $C_{20}H_{13}BrN_4O$

Bromodeoxytopsentin (**11**):[4] yellow amorphous powder (2.4 mg); UV (MeOH) λ_{max} 208, 250, 274, 370 nm; ^{1}H NMR (DMSO-d_6, 600MHz) δ 12.08 (s, H-1′), 11.47 (br s, H-1), 9.25 (br s, H-2′), 8.39 (m, H-4′), 8.02 (br, H-4), 7.98 (br, H-2), 7.69 (br, H-4″), 7.63 (m, H-7), 7.54 (m, H-7′), 7.23 (m, H-5), 7.23 (m, H-5′), 7.23 (m, H-6′); Mass spectrum (ESI+) *m/z*: isotopic cluster 405:407 (in ratio 1:1) $[M + H]^+$ for $C_{20}H_{13}BrN_4O$

Dibromodeoxytopsentin (**12**):[4] yellow amorphous powder (5.8 mg); UV (MeOH) λ_{max} 212, 254, 280, 370 nm; ^{1}H NMR (DMSO-d_6, 600MHz) δ 13.24 (br s, H-1″), 12.13 (s, H-1′), 11.46 (br s, H-1), 9.27 (br, H-2′), 8.42 (m, H-4′), 8.08 (br, H-4), 7.92 (br, H-2), 7.74 (d, *J* = 1.8 Hz, H-7′), 7.69 (br, H-4″), 7.63 (s, H-7), 7.39 (dd, 1.8, 8.5 Hz, H-5′), 7.23 (d, 7.7 Hz, H-5); Mass spectrum (ESI+) *m/z*: isotopic cluster 483:485:487 (in ratio 1:2:1) $[M + H]^+$ for $C_{20}H_{12}N_4OBr_2$

6-bromoindole-3-carboxylic acid (**13**):[5] light brown amorphous powder (1.9 mg); UV

(MeOH) λ_{max} 212, 250, 275, 330 nm; IR (neat film) ν_{max} 3323, 3126, 2973, 1645, 1525, 1446, 1227, 1184, 1131, 1021, 805 cm^{-1}; ^1H NMR (DMSO-d_6, 600MHz) δ 12.08 (br s, OH), 11.91 (br s, NH), 8.01 (d, J = 2.9 Hz H-2), 7.92 (d, J = 8.5 Hz H-4), 7.64 (d, J = 1.7 Hz H-7), 7.28 (dd, J = 1.8, 8.5 Hz H-5); ^{13}C-NMR (DMSO-d_6, 150MHz) δ 165.6 (C-8), 137.3 (C-3a), 133.2 (C-2), 125.0 (C-7a), 124.0 (C-5), 122.3 (C-4), 114.9 (C-7), 114.9 (C-3), 107.6 (C-6); Mass spectrum (ESI+) *m/z*: isotopic cluster 240:242 (in ratio 1:1) $[M + H]^+$; HRESIMS *m*/z 237.95070 $[M – H]^-$ (calcd. for $C_9H_5BrNO_2$, 237.95037).

(6-bromo-1*H*-indol-3-yl) oxoacetamide (**14**):[6] yellow amorphous powder (1.9 mg); UV (MeOH) λ_{max} 210, 325 nm; IR (neat film) ν_{max} 3384, 3184, 1662, 1616, 1591, 1513, 1404, 1135, 993, 925 cm^{-1}; ^1H NMR (DMSO-d_6, 600MHz) δ 12.25 (br s, NH), 8.69 (d, J = 3.17 Hz, H-2), 8.13 (d, J = 8.4 Hz, H-4), 8.09 (br s, H-10a), 7.74 (br s, H-10b), 7.72 (m, J = 1.8 Hz, H-7), 7.38 (dd, J = 1.8, 8.4 Hz, H-5); ^{13}C-NMR (DMSO-d_6, 150MHz) δ 183.0 (C-8), 165.7 (C-9), 139.1 (C-2), 137.3 (C-7a), 125.4 (C-5), 125.2 (C-3a), 122.9 (C-4), 115.9 (C-6), 115.3 (C-7), 112.0 (C-3); Mass spectrum (ESI+) *m/z*: isotopic cluster 267:269 (in ratio 1:1) $[M + H]^+$ for $C_{10}H_7N_2O_2$.

3,4-Seco-(*R*)-6″-debromohamacanthin A (**15**): yellow amorphous solid (43.2 mg); $[\alpha]^{20}_D$ –31 (*c* 0.50, MeOH); UV (MeOH) λ_{max} (log ε) 326 (3.92), 304 (3.82), 280 (4.15), 246 (3.96) nm; ECD (*c* 0.06 mM, MeOH) λ_{max} (Δε) 362 (+0.4), 330 (–2.74), 287 (–1.05) 241 (–2.75) nm; IR (neat film) ν_{max} 3232, 1672, 1627, 1488, 1439, 1201, 1180, 1130, 997, 744 cm^{-1}; Mass spectrum (ESI+) *m/z*: isotopic cluster 425:427 (in ratio 1:1) $[M + H]^+$ for $C_{20}H_{18}BrN_4O_2^+$.

(*R*)-6″-debromohamacanthin A (**22**): $[\alpha]^{23}_D$ –82 (*c* 0.05, MeOH) Lit.[7] –76 (*c* 0.05, MeOH).

3,4-Seco-(*R*)-6′,6″-didebromohamacanthin A (**16**): yellow amorphous solid (14.9 mg); $[\alpha]^{20}_D$ –26 (*c* 0.40, MeOH); UV (MeOH) λ_{max} (log ε) 328 (3.70), 298 (3.52), 269 (3.89), 243 (3.70) nm; ECD (*c* 0.07 mM, MeOH) λ_{max} (Δε) 363 (+0.2), 331 (–1.57), 280 (–0.83), 239 (–2.85) nm; IR (neat film) ν_{max} 3246, 2980, 1672, 1620, 1490, 1430, 1240, 1130, 997, 745 cm^{-1}; Mass spectrum (ESI+) *m/z*: 347 $[M + H]^+$ for $C_{20}H_{19}N_4O_2^+$.

(*R*)-6′,6″-didebromohamacanthin A (**23**): $[\alpha]^{23}_D$ –34 (*c* 0.05, MeOH) Lit.[8] +59 (*c* 0.72, MeOH)

3,4-Seco-(*S*)-Hamacanthin A (**17**): yellow amorphous solid (2.9 mg); $[\alpha]^{20}_D$ –10 (*c* 0.10, MeOH); UV (MeOH) λ_{max} (log ε) 322 (4.20), 300 (4.08), 277 (4.37), 259 (4.33) nm; ECD (*c* 0.05 mM, MeOH) λ_{max} (Δε) 360 (–0.05), 331 (+0.28), 289 (+0.20) 260 (–0.03) nm; IR (neat film) ν_{max} 3157, 2975, 2901, 1670, 1615, 1489, 1440, 1131, 896, 799 cm^{-1}; Mass spectrum (ESI+) *m/z*: isotopic cluster 503:505:507 (in ratio 1:2:1) $[M + H]^+$ for $C_{20}H_{17}Br_2N_4O_2^+$.

(*S*)-Hamacanthin A (**24**) $[\alpha]^{23}_D$ +64 (*c* 0.05, MeOH) Lit.[8-9] +83.7 (*c* 0.47, MeOH), +58 (*c* 0.05, MeOH)

3,4-Seco-(*S*)-Hamacanthin B (**18**): light brown amorphous solid (2.0 mg); $[\alpha]^{20}_D$ +7 (*c* 0.50, MeOH); UV (MeOH) λ_{max} (log ε) 330 (3.38), 311 (3.38), 280 (3.73), 245 (3.81) nm; ECD (*c* 0.05 mM, MeOH) λ_{max} (Δε) 370 (–0.01), 339 (+0.26), 284 (+0.24) 230 (+0.71) nm; IR (neat film) ν_{max} 3232, 1672, 1627, 1488, 1439, 1201, 1180, 1130, 997, 744 cm^{-1}; Mass spectrum (ESI+) *m/z*: isotopic cluster 503:505:507 (in ratio 1:2:1) $[M + H]^+$ $C_{20}H_{17}Br_2N_4O_2^+$.

(*S*)-Hamacanthin B (**25**): $[\alpha]^{23}_D$ +46 (*c* 0.05, MeOH) Lit.[8, 10] +56 (*c* 0.2, MeOH), +172 (*c* 0.1, MeOH)

3,4-Seco-(*S*)-6″-debromohamacanthin B (**19**): orange amorphous solid (5.2 mg); $[\alpha]^{20}_D$ –27 (*c* 0.25, MeOH); UV (MeOH) λ_{max} (log ε) 326 (3.46), 306 (3.43), 268 (3.67), 242 (3.69) nm; ECD (*c* 0.12 mM, MeOH) λ_{max} (Δε) 337 (+0.61), 278 (+0.55) 230 (+1.32) nm; IR

(neat film) v_{max} 3141, 1670, 1616, 1498, 1418, 1199, 1128, 797, 743 cm^{-1}; Mass spectrum (ESI+) *m/z*: isotopic cluster 425:427 (in ratio 1:1) $[M + H]^+$ for $C_{20}H_{18}BrN_4O_2^+$.

(*S*)-6″-debromohamacanthin B (**26**): $[\alpha]^{23}_D$ +36 (*c* 0.05, MeOH) Lit.[7] +43 (*c* 0.3, MeOH)

3,4-Seco-(*R*)-6′-debromohamacanthin B (**20**): yellow amorphous solid (43.2 mg); $[\alpha]^{20}_D$ +19 (*c* 0.95, MeOH); UV (MeOH) λ_{max} (log ε) 329 (3.49), 305 (3.46), 280 (3.75), 245 (3.73) nm; ECD (*c* 0.06 mM, MeOH) λ_{max} (Δε) 365 (+0.05), 332 (–0.55), 287 (–0.57) 234 (–1.90) nm; IR (neat film) v_{max} 3216, 2935, 1671, 1617, 1492, 1429, 1201, 1180, 1131, 997, 800, 750 cm^{-1}; Mass spectrum (ESI+) *m/z*: isotopic cluster 425:427 (in ratio 1:1) $[M + H]^+$ for $C_{20}H_{18}BrN_4O_2^+$.

6′-debromohamacanthin B (**27**): $[\alpha]^{23}_D$ +38 (*c* 0.05, MeOH) Lit.[8] –194 (*c* 0.25, MeOH);

3,4-Seco-(*R*)-6′,6″-didebromohamacanthin B (**21**): yellow amorphous solid (14.9 mg); $[\alpha]^{20}_D$ –16 (*c* 0.50, MeOH); UV (MeOH) λ_{max} (log ε) 329 (3.59), 300 (3.50), 267 (3.81), 242 (3.73) nm; ECD (*c* 0.07 mM, MeOH) λ_{max} (Δε) 370 (+0.05), 332 (–0.65), 277 (–0.59) 230 (–1.51) nm; IR (neat film) v_{max} 3243, 2976, 1670, 1617, 1490, 1429, 1200, 1128, 1000, 744 cm^{-1}; Mass spectrum (ESI+) *m/z*: 347 $[M + H]^+$ for $C_{20}H_{19}N_4O_2^+$.

(*R*)-6′,6″-didebromohamacanthin B (**28**): $[\alpha]^{20}_D$ –36 (*c* 0.04, MeOH) Lit.[8] –288 (*c* 0.4, MeOH)

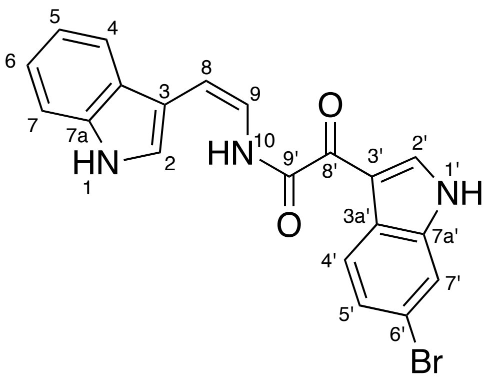

Table S1. NMR data (600 MHz) for (*Z*)-coscinamide D (**1**) in DMSO-d_6

Position	δ_C, type	δ_H (J in Hz)	COSY	HMBC	ROESY
1-NH		11.45, s	2	2,3,3a,7a	2,7,9
2	123.8, CH	7.64, m	1	3,3a,7a,8	1,8,9
3	109.6, C				
3a	126.4, C				
4	111.7, CH	7.43, d (8.1)	5	3a,6	5,8
5	122.0, CH	7.16, td (7.4, 1.0)	4,6	7,7a	4
6	119.4, CH	7.07, td (7.4, 1.0)	5,7	3a,4,7a	7
7	118.4, CH	7.63, m	6	5,7a	1,6
7a	135.8, C				
8	106.1, CH	6.24, d (9.2)	9	2,3a,9	2,4,9
9	117.2, CH	6.80, dd (11.0, 9.2)	8,10	3,8,9′	1,8,10
10-NH		9.66, d (11.0)	9	8,9′	2,9
1′-NH		12.43, br	2′	2′,3′,3a′	7′,2′
2′	139.9, CH	8.92, d (3.3)	1′	3′,3a′,7a′	1′
3′	111.8, C				
3a′	125.3, C				
4′	123.0, CH	8.16, d (8.4)	5′	3′,6′,7a′	5′
5′	125.6, CH	7.41, dd (8.4, 1.8)	4′	3a′,7′	4′
6′	116.1, C				
7′	115.4, CH	7.76, d (1.8)		3a′,6′,7a′	1′
7a′	137.2, C				
8′	180.0, C				
9′	159.7, C				

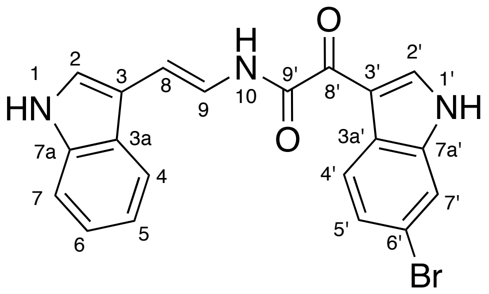

Table S2. NMR data (600 MHz) for (*E*)-coscinamide D (**2**) in DMSO-d_6

Position	δ_C, type	δ_H (J in Hz)	COSY	HMBC	ROESY
1-NH		11.21, s	2	3,3a,7a	2,7
2	124.4, CH	7.49, d (2.4)	1	3,3a,7a	1,8
3	111.6, C				
3a	124.8, C				
4	119.0, CH	7.68, d (7.8)	5	3,3a,6,7a	5,9
5	119.5, CH	7.09, t (7.4)	4,6	3a,7	4
6	121.6, CH	7.14, t (7.4)	5,7	4,7a	7
7	111.9, CH	7.39, m	6	3a,5	1,6
7a	136.9, C				
8	110.2, CH	6.85, d (14.7)	9	2,3a,9	2,10
9	118.5, CH	7.40, m	8,10	3,8	4,10
10-NH		10.85, d (9.9)	9	8, 9′	8,9
1′-NH		12.36, br	2′	3′,3a′	7′,2′
2′	139.4, CH	8.85, d (3.2)	1′	3′,3a′,7a′	1′
3′	112.2, C				
3a′	125.3, C				
4′	122.9, CH	8.19, d (8.5)	5′	6′,7a′	5′
5′	125.5, CH	7.43, m	4′	3a′,7′	4′
6′	116.0, C				
7′	115.4, CH	7.75, d (1.8)		3a′,6′	1′
7a′	137.3, C				
8′	181.3, C				
9′	160.0, C				

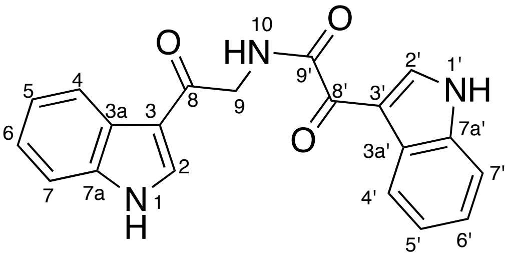

Table S3. NMR data (600 MHz) for Lamellomorphamide A (**3**) in DMSO-d_6

Position	δ_C, type	δ_H (J in Hz)	COSY	HMBC	ROESY
1-NH		12.07, br	2	3,3a	2,7
2	133.8, CH	8.50, d (3.2)	1	3,3a,7a	1,9
3	113.9, C				
3a	125.4, C				
4	121.1, CH	8.16, m	5	6,7a	5,9
5	121.9, CH	7.20, td (7.1, 1.1)	4	3a,7	4
6	122.6, CH	7.23, td (7.1, 1.1)	7	4,7a	7
7	112.6, CH	7.49, m	6	3a,5	1,6
7a	136.3, C				
8	189.2 C				
9	45.7, CH_2	4.63, d (5.9)	10	8,9′	2,10
10-NH		8.91, t (5.9)	9	8,9′	9
1′-NH		12.26, br	2′	3′,3a′,7a′	2′,7′
2′	138.7, CH	8.82, d (3.2)	1′	3′,3a′,7a′	1′
3′	112.3, C				
3a′	126.2, C				
4′	121.3, CH	8.26, m	5′	6′,7a′	5′,6′
5′	122.9, CH	7.27, m	4′	3a′,7′	4′,^{4}7′
6′	123.5, CH	7.28, m	7′	4′	4′
7′	112.2, CH	7.54, m	6′	3a′,5′	1′,5′
7a′	136.4, C				
8′	181.9, C				
9′	163.8, C				

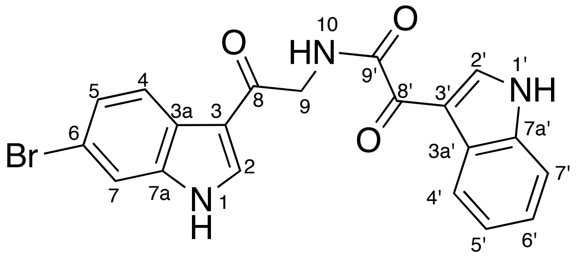

Table S4. NMR data (600 MHz) for Lamellomorphamide B (**4**) in DMSO-d_6

Position	δ_C, type	δ_H (J in Hz)	COSY	HMBC	ROESY
1-NH		12.17, br	2	2,3,3a,7a	2,7
2	134.7, CH	8.53, d (3.0)	1	3,3a,7a	1,9
3	113.9, C				
3a	124.5, C				
4	122.7, CH	8.09, d (8.5)	5	6,7a	5,9
5	124.9, CH	7.35, dd (8.5,1.8)	4	3a,7	4
6	115.0, C				
7	115.6, CH	7.69, d (1.8)		3a,5,6,7a	1
7a	137.4, C				
8	189.4, C				
9	45.7, CH_2	4.62, d (6.0)	10	8,9′	2,4,10
10-NH		8.93, t (5.3)	9	9,9′	9
1′-NH		12.25, br	2′	2′,3′,3a′,7a′	2′,7′
2′	138.6, CH	8.80, d (3.3)	1′	3′,3a′,7a′	1′
3′	112.3, C				
3a′	126.2, C				
4′	121.3, CH	8.25, m	5′	6′,7a′	5′,6′
5′	122.9, CH	7.27, m	4′	3a′,7′	4′,7′
6′	123.6, CH	7.28, m	7′	4′,5′,7a′	4′
7′	112.7, CH	7.53, m	6′	3a′,5′	1′,5′
7a′	136.3, C				
8′	181.8, C				
9′	163.8, C				

[a] Obscured by H_2O signal

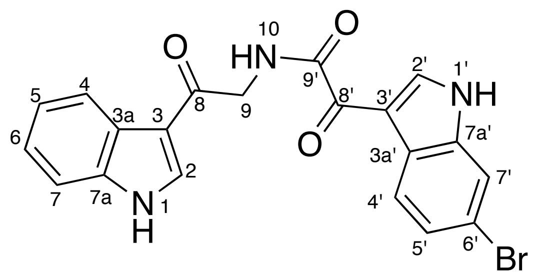

Table S5. NMR data (600 MHz) for Lamellomorphamide C (**5**) in DMSO-d_6

Position	δ_C, type	δ_H (J in Hz)	COSY	HMBC	ROESY
1-NH		12.06, br	2	2,3,3a,7a	2,7
2	133.8, CH	8.50, d (3.2)	1	3,3a,7a	1,9
3	113.9, C				
3a	125.2, C				
4	121.1, CH	8.16, m (7.6)	5	6,7a	5,9
5	121.9, CH	7.20, td (7.1, 1.1)	4,6	3a,7	4
6	122.9, CH	7.23, td (7.1, 1.1)	5,7	4,7a	7
7	112.2, CH	7.49, m	6	3a,5	1,6
7a	136.4, C				
8	189.1, C				
9	45.7, CH_2	4.63, d (5.8)	10	8,9′	2,4,10
10-NH		8.94, t (5.8)	9	8,9,9′	9
1′-NH		12.32, br	2′	2′,3′,3a′	2′,7′
2′	139.4, CH	8.83, d (3.2)	1′	3′,3a′,7a′	1′
3′	112.2, C				
3a′	125.3, C				
4′	123.0, CH	8.18, d (8.4)	5′	3′,3a′,6′,7a′	5′
5′	125.5, CH	7.42, dd (8.4, 1.8)	4′	3a′,7′	4′
6′	116.0, C				
7′	115.3, CH	7.75, dd (1.8)		3a′,6′,7a′	1′
7a′	137.2, C				
8′	181.9, C				
9′	163.4, C				

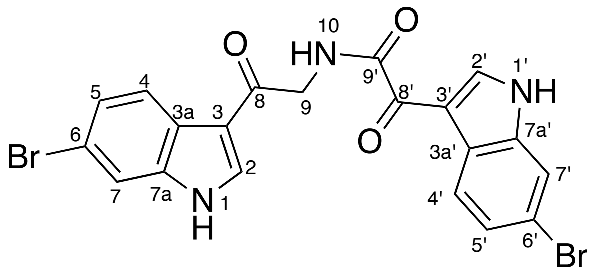

Table S6. NMR data (600 MHz) for Lamellomorphamide D (**6**) in DMSO-d_6

Position	δ_C, type	δ_H (*J* in Hz)	COSY	HMBC	ROESY
1-NH		12.17, br	2	3,3a	2,7
2	134.6, CH	8.53, d (3.1)	1	3,3a,7a	1,9
3	113.9, C				
3a	124.4, C				
4	122.8, CH	8.09, d (8.5)	5	6,7a	5,9
5	124.9, CH	7.35, dd (8.5,1.8)	4	3a,7	4
6	115.5, C				
7	114.9, CH	7.69, d (1.8)		3a,6,7a	1
7a	137.2, C				
8	189.1, C				
9	45.7, CH_2	4.62, d (5.9)	10	8,9′	2,4,10
10-NH		8.96, t (5.9)	9	8,9,9′	9
1′-NH		12.32, br	2′	3′,3a′	2′,7′
2′	139.4, CH	8.81, d (3.2)	1′	3′,3a′,7a′	1′
3′	112.2, C				
3a′	125.2, C				
4′	123.0, CH	8.18, d (8.4)	5′	6′,7a′	5′
5′	125.5, CH	7.42, dd (8.4,1.8)	4′	3a′,7′	4′
6′	116.0, C				
7′	115.4, CH	7.75, d (1.8)		3a′,6′,7a′	1′
7a′	137.3, C				
8′	181.9, C				
9′	163.5, C				

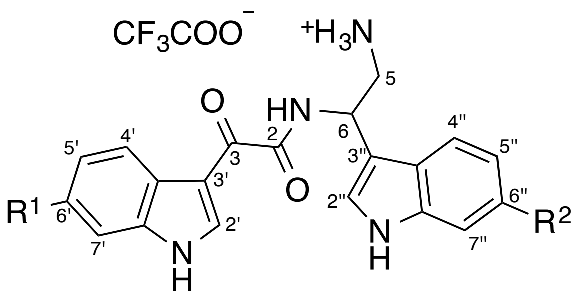

15 R[1] = Br, R[2] = H
16 R[1] = R[2] = H
17 R[1] = R[2] = Br

Table S7. NMR data (600 MHz) for **15-17** in DMSO-d_6

	**15**		**16**		**17**	
Position	δ_C, Type	δ_H, (J in Hz)	δ_C, Type	δ_H, (J in Hz)	δ_C, Type	δ_H, (J in Hz)
1		9.13, d (9.2)		9.11, d (9.3)		9.18, d (9.2)
2	162.8, C		163.1, C		162.9, C	
3	181.1, C		181.2, C		181.3, C	
4		8.05, br, s		7.97, br		7.94, br
5	42.12, CH_2	3.51, 3.37[a]	42.2, CH_2	3.49, 3.36[a]	42.1, CH_2	3.48, 3.35[a]
6	43.8, CH	5.59, td (9.2, 4.2)	43.8, CH	5.58, m	43.6, CH	5.54, m
1′		12.44, d (2.8)		12.27, br		12.32, s, d
2′	139.4, CH	8.84, d (3.1)	138.7, C	8.82, d (3.2)	139.4, CH	8.82, d (3.2)
3′	112.1, C		111.8, C		112.1, C	
3a′	125.6, C		126.4, C		125.4, C	
4′	122.9, CH	8.14, d (8.4)	121.3, CH	8.22, m	122.9, CH	8.13, d (8.4)
5′	125.5, CH	7.39 (m)	123.5, CH	7.26, m	125.6, CH	7.39, dd (1.6, 8.4)
6′	116.0, C		122.7, C	7.24, m	116.1, C	
7′	115.4, CH	7.75, d (1.6)	112.7, CH	7.54, m	115.4, CH	7.75, d (1.6)
7a′	137.2, C		136.2, C		137.2, C	
1″		11.17, d (2.0)		11.15, br		11.28, s
2″	123.3, CH	7.40, m	123.4, CH	7.40, m	124.6, CH	7.42, d (2.6)
3″	112.1, C		112.2, C		112.6, C	
3a″	125.4, C	7.39	125.6, C		124.7, C	
4″	111.8, CH	7.10, t (7.4)	111.8, CH	7.39, m	120.3, CH	7.62, d (8.5)
5″	121.5, CH	7.02, t (7.4)	121.5, CH	7.10, m	121.9, CH	7.18, dd (1.7, 8.5)
6″	119.0, CH	7.67, d (8.0)	119.0, CH	7.02, m	114.3, C	
7″	118.4, CH		118.5, CH	7.67, d (8.0)	114.4, CH	7.58, d (1.6)
7a″	136.1, C		136.2, C		137.1, C	

[a] Obscured by H_2O signal

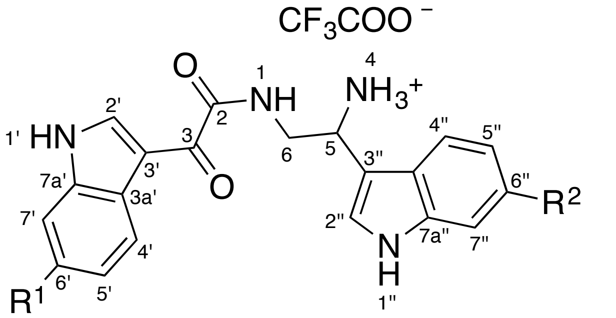

18 $R^1 = R^2 = Br$
20 $R^1 = H$, $R^2 = Br$

Table S8. NMR data (600 MHz) for **18** and **20** in DMSO-d_6

	**18**		**20**	
Position	δ_C, Type	δ_H, (J in Hz)	δ_C, Type	δ_H, (J in Hz)
1		8.98, t (5.8)		8.95, t (5.9)
2	163.2, C		163.6, C	
3	181.0, C		180.8, C	
4		8.30, br, s		8.34, br, d (3.8)
5	46.9, CH	4.81, br, s	46.8, CH	4.83, m
6	42.1, CH_2	3.81, m; 3.67, m	42.1, CH_2	3.82, m; 3.69, m
1′		12.31, d (2.9)		12.27, br, d (2.3)
2′	139.4, CH	8.78, d (2.9)	138.6, CH	8.76, d (3.3)
3′	112.0, C		112.0, C	
3a′	125.3, C		126.2, C	
4′	122.9, CH	8.13, d (8.4)	121.2, CH	8.21, m
5′	125.3, CH	7.40, dd (8.4, 1.8)	122.6, CH	7.25, m
6′	116.0, C		123.5, CH	7.26, m
7′	115.4, CH	7.75, d (1.8)	112.6, CH	7.54, m
7a′	137.1, C		136.7, C	
1″				11.46, s
2″	125.2, CH	11.44, d (2.6)	125.2, CH	7.57, d (2.4)
3″	109.6, C	7.56, d (2.6)	109.5, C	
3a″	124.7, C		124.7, C	
4″	120.3, CH	7.69, d (8.5)	120.3, CH	7.70, d (8.4)
5″	122.1, CH	7.22, dd (8.5, 1.7)	122.1, CH	7.22, dd (8.4, 1.7)
6″	114.4, C		114.5, C	
7″	114.6, CH	7.62, d (1.7)	114.4, CH	7.63, d (1.7)
7a″	136.8, C		136.8, C	

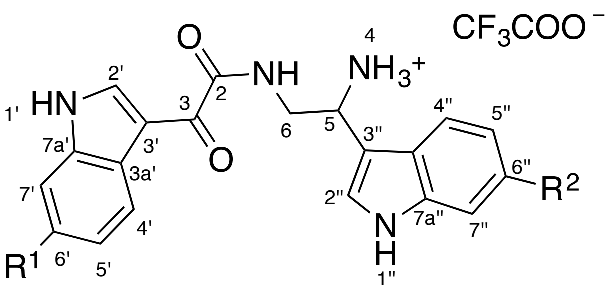

19 R¹ = Br, R² = H
21 R¹ = R² = H

Table S9. NMR data (600 MHz) for **19** and **21** in DMSO-d_6

	**19**		**21**	
Position	δ_C, Type	δ_H, (J in Hz)	δ_C, Type	δ_H, (J in Hz)
1		8.99, t (6.0)		8.97, t (6.0)
2	163.3, C		163.7, C	
3	180.9, C		180.9, C	
4		8.30, br, s		8.30, br, s
5	47.0, CH	4.83, m	47.1, CH	4.83, m
6	42.2, CH_2	3.84, m; 3.70, m	42.2, CH_2	3.84, m; 3.71, m
1′		12.34, s		12.26, br
2′	139.4, CH	8.79, d (3.4)	138.7, CH	8.78, d (3.2)
3′	111.9, C		112.1, C	
3a′	125.5, C		126.3, C	
4′	122.9, CH	8.14, d (8.4)	121.3, CH	8.22, m
5′	125.5, CH	7.40, dd (1.8, 8.4)	122.7, CH	7.25, m
6′	116.0, C		123.6, CH	7.26, m
7′	115.3, CH	7.75, d (1.8)	112.7, CH	7.54, m
7a′	137.1, C		136.1, C	
1″		11.32, s		11.32, br
2″	124.0, CH	7.54, d (2.6)	125.6, CH	7.54, m
3″	109.1, C		109.2, C	
3a″	125.4, C		124.1, C	
4″	118.4, CH	7.72, d (8.0)	118.4, CH	7.73, d (8.0)
5″	119.2, CH	7.08, td (1.0, 7.5)	119.2, CH	7.08, t (7.5)
6″	121.8, C	7.15, td (1.0, 7.5)	121.9, C	7.15, t (7.5)
7″	111.8, CH	7.42, td (8.0)	111.9, CH	7.42, d (8.0)
7a″	135.9, C		136.0, C	

Table S10. Bioassay results of compounds **1-9**, **13-21**

	S. aureus (MRSA, ATTC 43300)	
**Compound**	Percentage inhibition (%) 10 μM (stdev)	Percentage inhibition (%) 20 μM (stdev)
**1**	-8.3 (7.8)	14.3 (10.1)
**2**	6.8 (4.1)	18.2 (6.1)
**3**	11.0 (3.6)	16.6 (10.0)
**4**	-2.8 (8.1)	14.0 (12.8)
**5**	5.5 (2.1)	5.8 (8.1)
**6**	9.3 (3.2)	14.9 (10.6)
**7**	-0.1 (9.3)	9.2 (1.5)
**8**	7.3 (3.9)	3.8 (7.6)
**9**	8.6 (4.3)	3.3 (11.4)
**13**	3.1 (3.2)	18.6 (13.2)
**14**	8.4 (1.8)	4.7 (7.4)
**15**	4.1 (1.7)	7.1 (6.2)
**16**	7.3 (0.4)	3.8 (7.6)
**17**	8.7 (2.9)	4.2 (6.7)
**18**	10.7 (3.6)	18.9 (7.1)
**19**	-10.0 (8.4)	4.1 (13.9)
**20**	15.3 (7.9)	-4.5 (16.7)
**21**	-5.4 (4.6)	18.9 (11.7)

Antibacterial assay

Percentage growth inhibition of an individual sample was calculated based on negative controls (media only) and positive controls (bacterial media without inhibitors). Negative inhibition value meant that the growth rate (or OD_{600}) was higher compared to the negative control (Bacteria only, set to 0% inhibition).

A

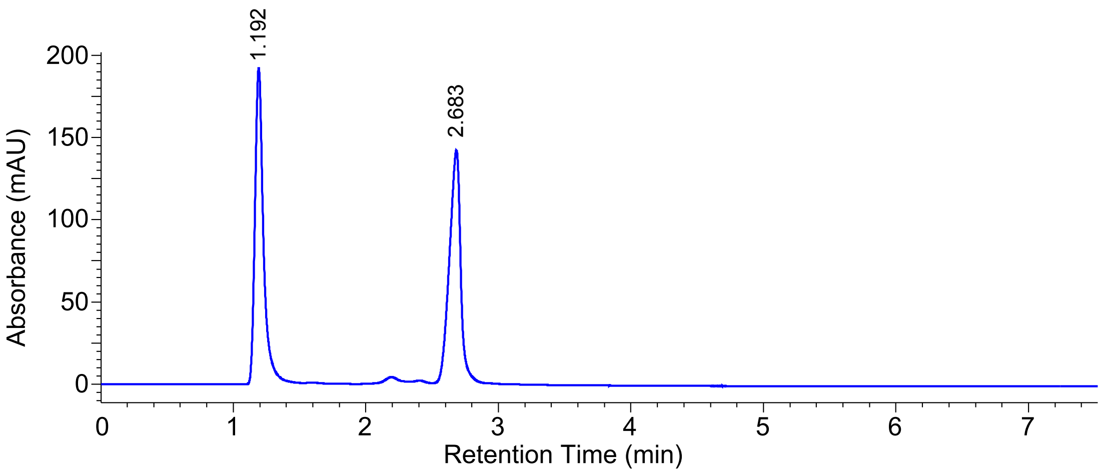

B

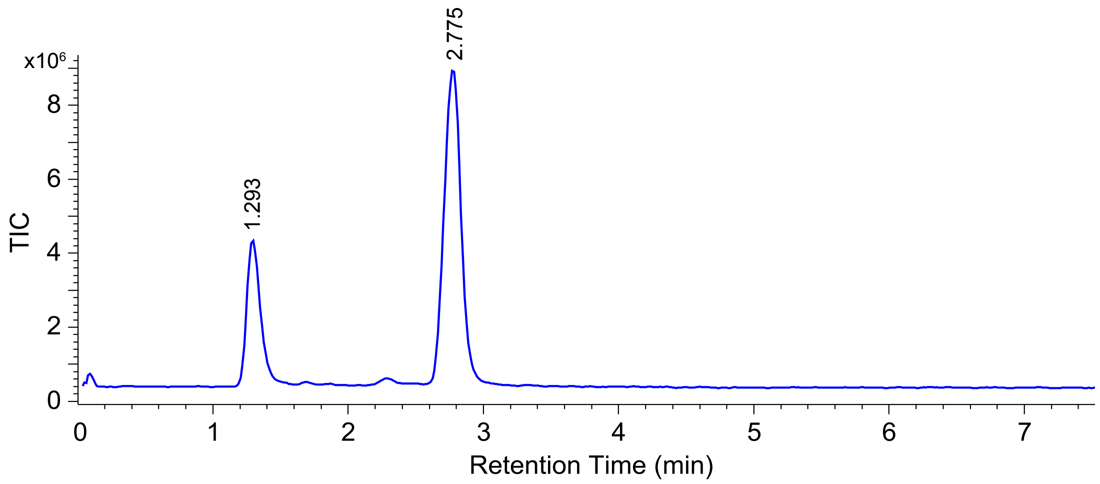

Figure S1. HPLC chromatogram of compound **15**
Run on an Agilent 6130B single quadrupole LCMS system using a C_{18} column with a gradient of 5-95% acetonitrile/water (0.025 % formic acid) at flowrate 0.25 mL/min with either A) UV detection at 254nm or B) TIC, positive ion mode.

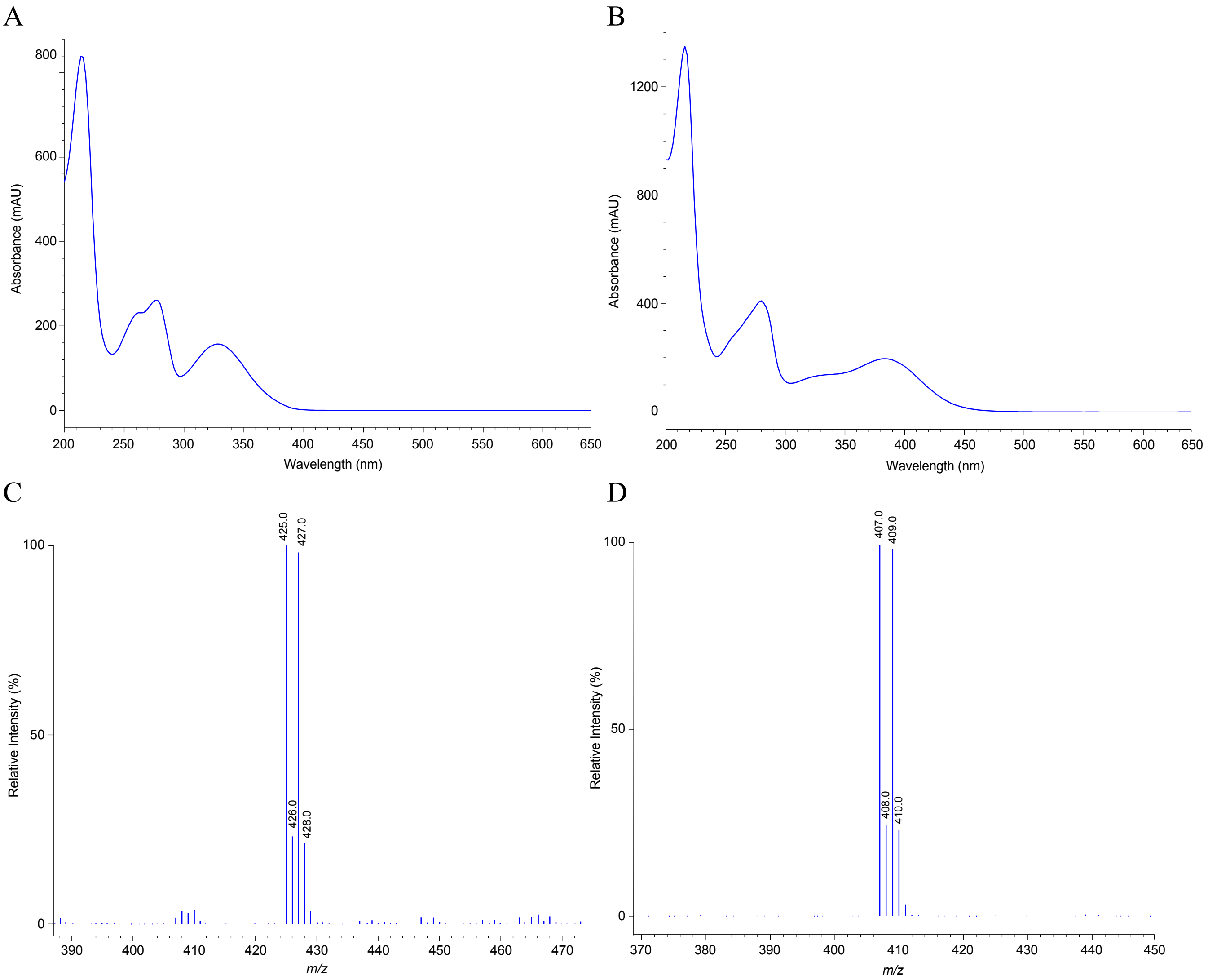

Figure S2. UV-Vis and Mass spectra of peaks eluting in Figure S1.
A) UV-Vis spectrum of the peak at 1.2 min. B) UV-Vis spectrum of the peak at 2.7 min. C) Mass spectrum of the peak eluting at 1.3 min. D) Mass spectrum of the peak 2.7 min.

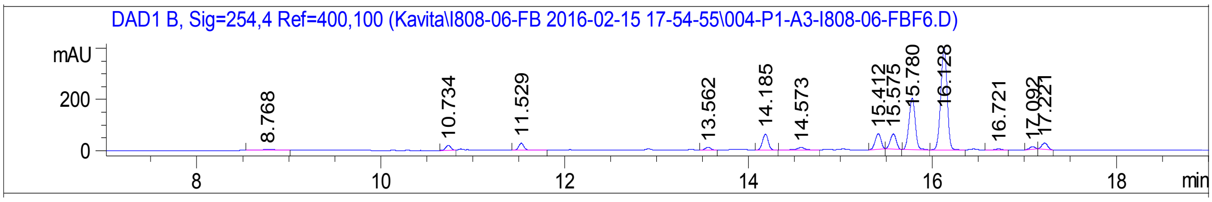

Figure S3. HPLC chromatogram of *n*-butanol fraction with UV detection at 254 nm. Run on an Agilent 6130B single quadrupole LCMS system using a C18 column with a gradient of 5-95% acetonitrile/water (0.025 % formic acid) at flowrate 0.25 mL/min.

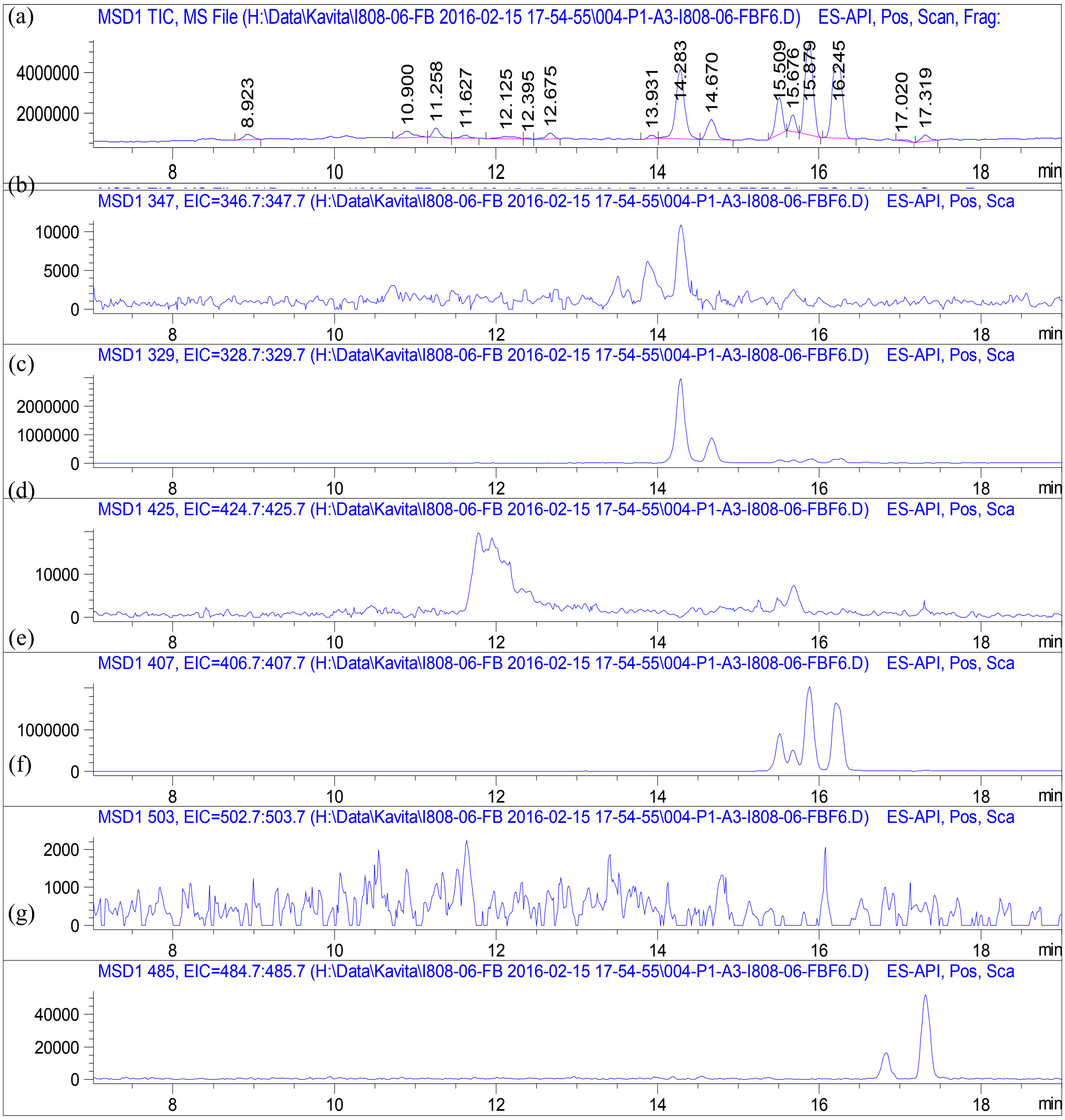

Figure S4. TIC and EIC of positive ion scan of *n*-butanol fraction
(a) TIC of positive ion scan, under the same conditions as Figure S76. (b) EIC of open ring form (m/z 347) 3,4-seco-6′,6″-didebromohamacanthin A (**16**) and 3,4-seco-6′,6″-didebromohamacanthin B (**21**) (c) EIC of closed ring form (m/z 329) 6′,6″-didebromohamacanthin A (**23**) and 6′,6″-didebromohamacanthin B (**28**) (d) EIC of open ring form (m/z 425) 3,4-seco-6″-debromohamacanthin A (**15**), 3,4-seco-6″-debromohamacanthin B (**19**), 3,4-seco-6′-debromohamacanthin B (**20**) (e) EIC of closed ring form (m/z 407) 6″-debromohamacanthin A (**22**), 6″-debromohamacanthin B (**26**), 6′-debromohamacanthin B (**27**) (f) EIC of open ring form (m/z 503) 3,4-seco-hamacanthin A (**17**) and 3,4-seco-hamacanthin B (**18**) (g) EIC of closed ring form (m/z 485) Hamacanthins A (**24**) and B (**25**)

UV and CD spectra

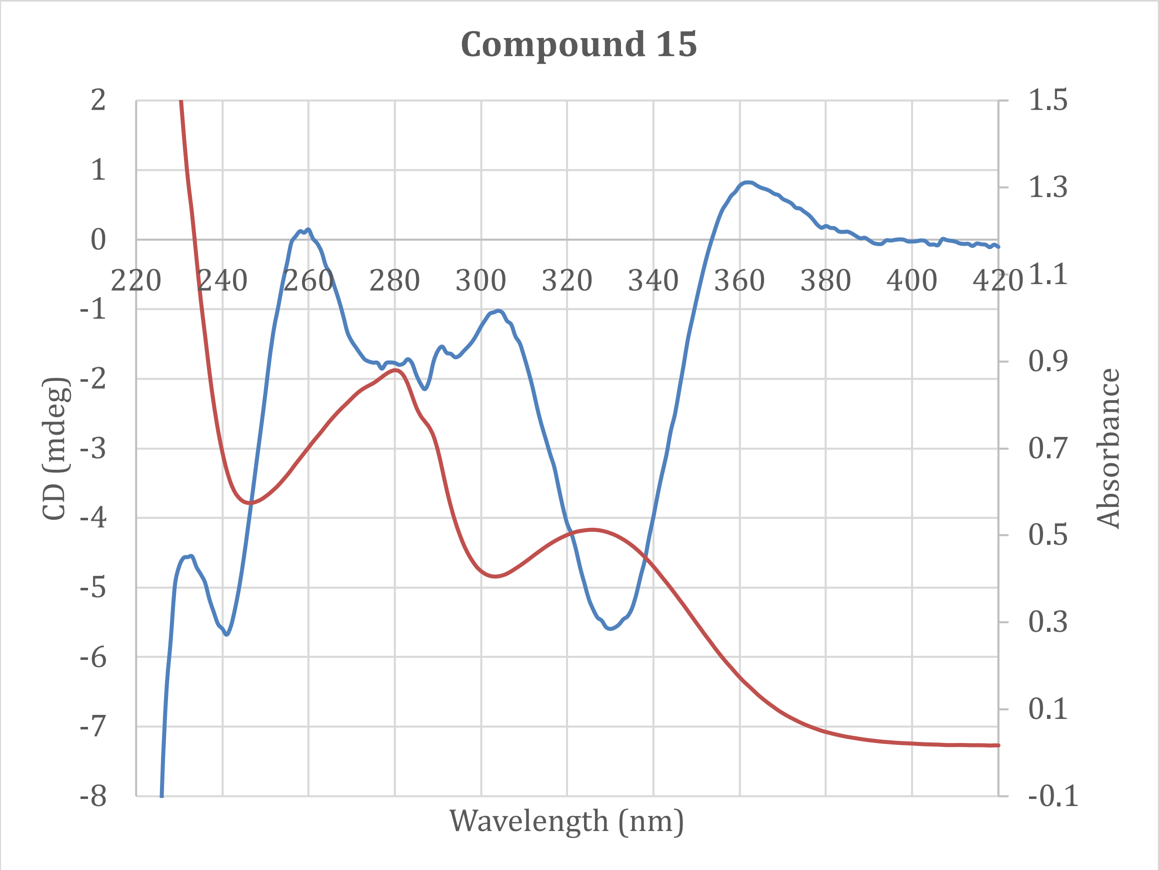

Figure S5. ECD and UV spectra of **15** (*c* 0.06 mM, MeOH)
Cell path 10 mm (UV - orange trace, ECD - blue trace)

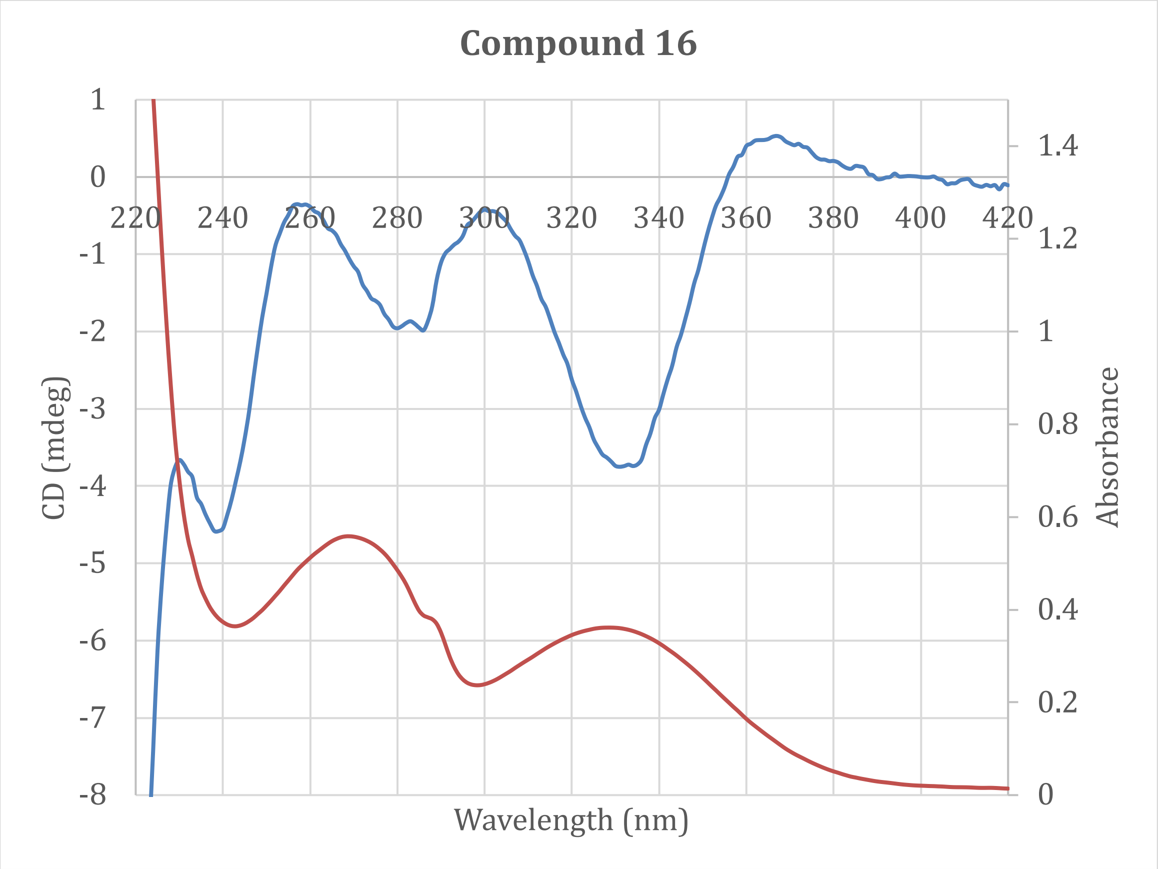

Figure S6. ECD and UV spectra of **16** (*c* 0.07 mM, MeOH)
Cell path 10 mm (UV - orange trace, ECD - blue trace)

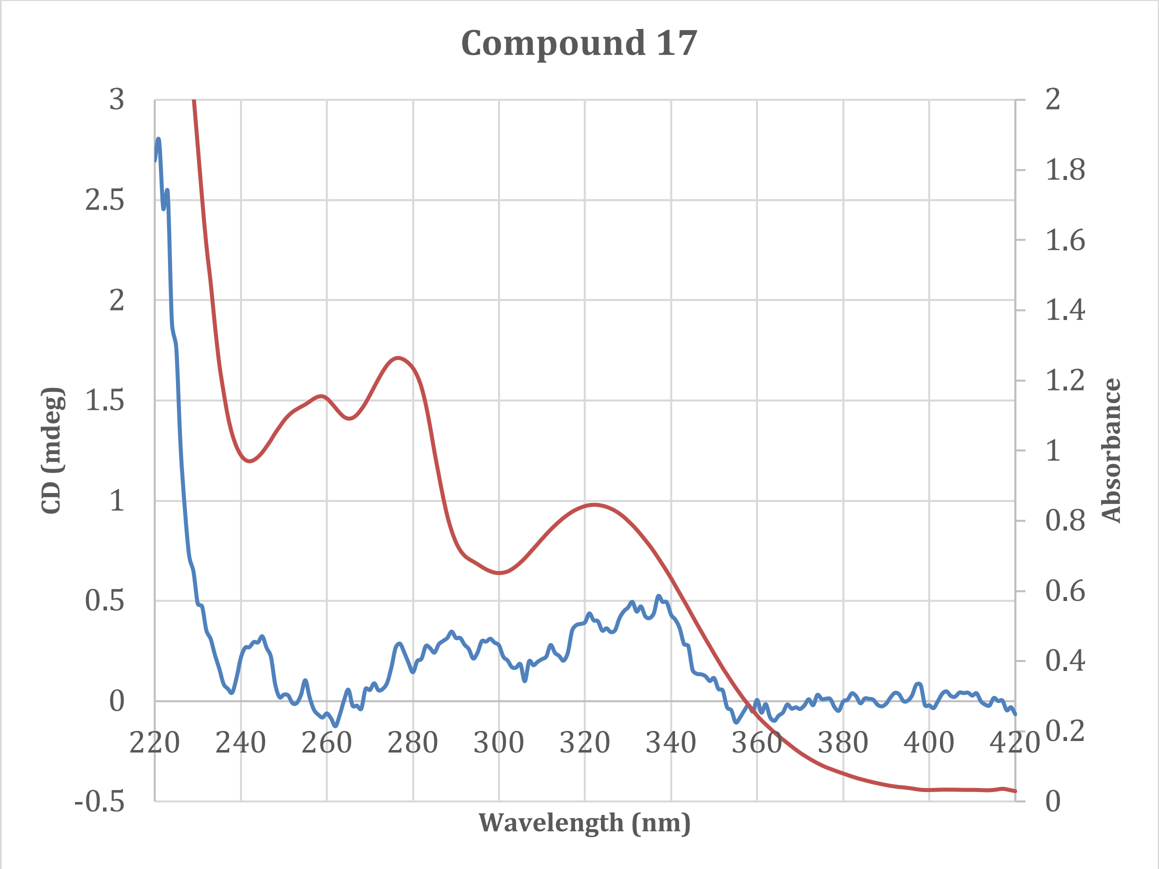

Figure S7. ECD and UV spectra of **17** (*c* 0.05 mM, MeOH)
Cell path 10 mm (UV - orange trace, ECD - blue trace)

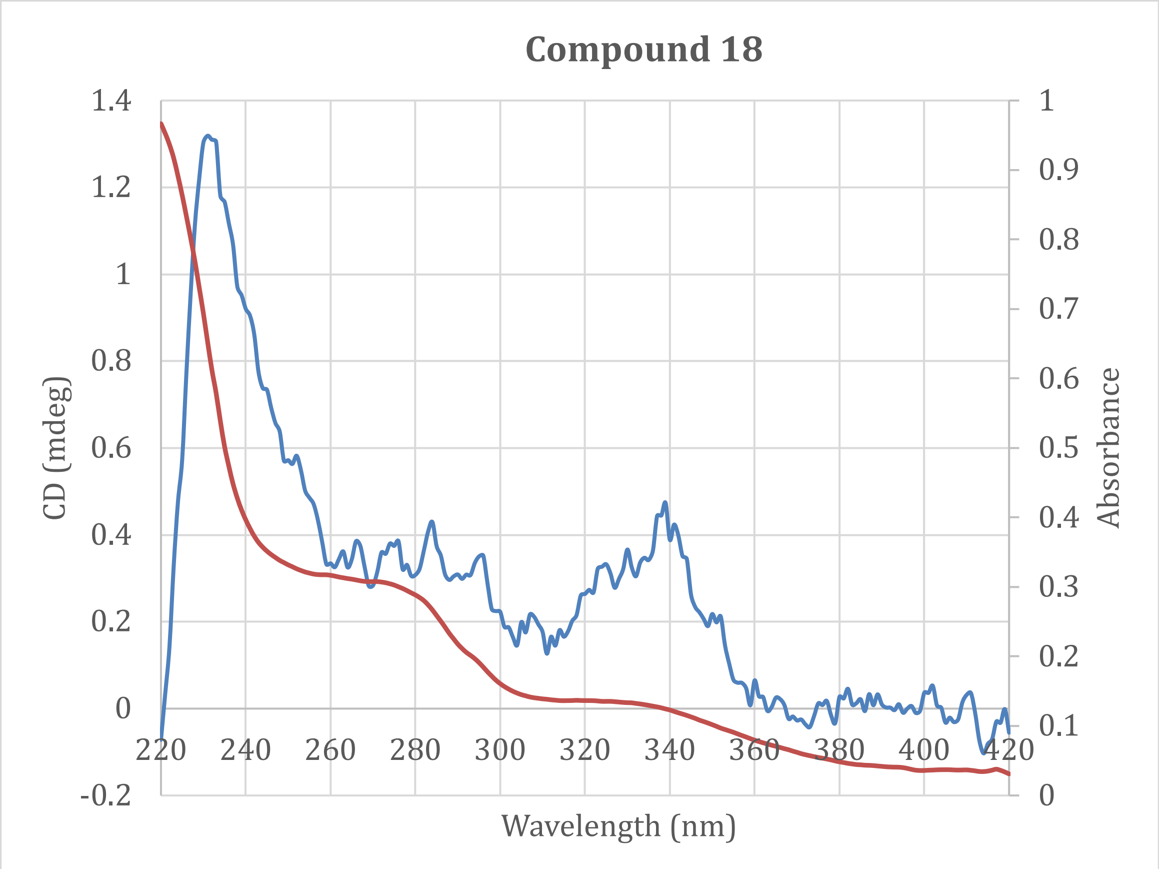

Figure S8. ECD and UV spectra of **18** (*c* 0.05 mM, MeOH)
Cell path 10 mm (UV - orange trace, ECD - blue trace)

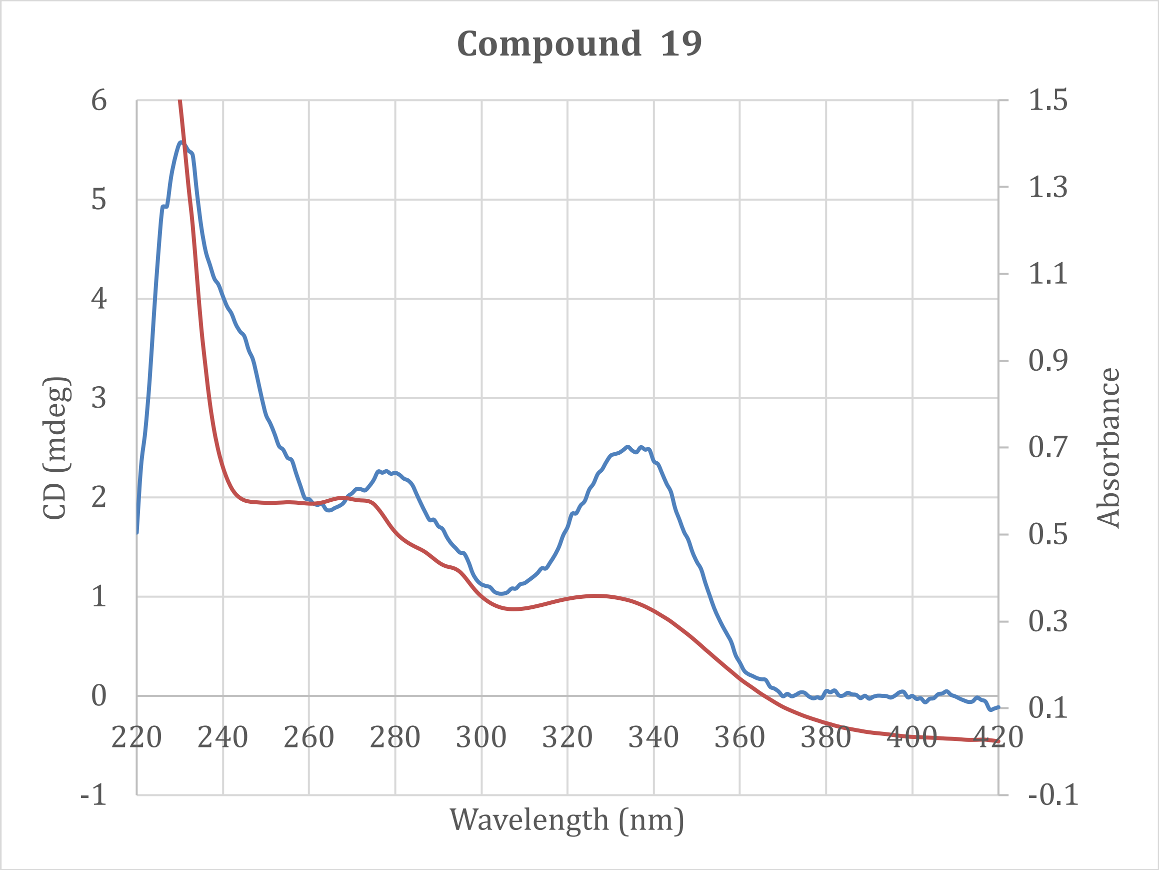

Figure S9. ECD and UV spectra of **19** (*c* 0.12 mM, MeOH)
Cell path 10 mm (UV - orange trace, ECD - blue trace)

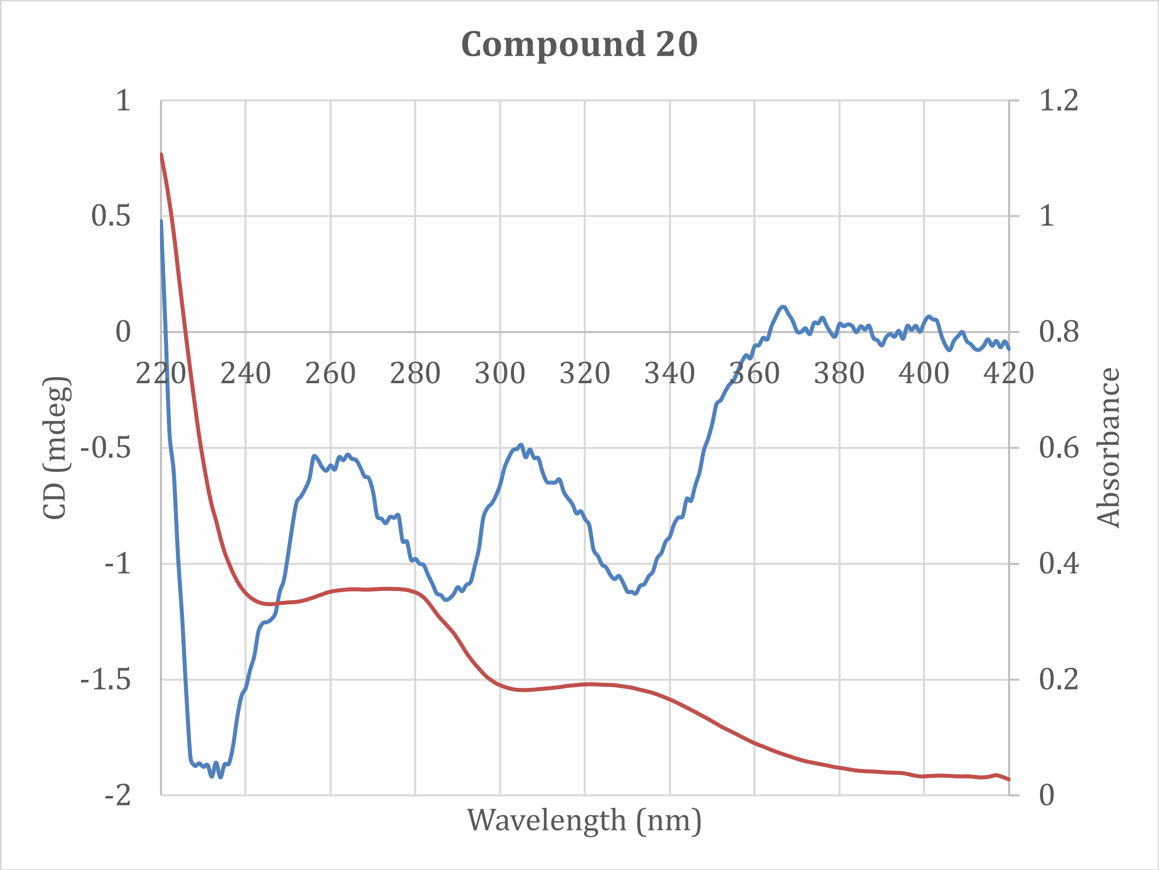

Figure S10. ECD and UV spectra of **20** (*c* 0.06 mM, MeOH)
Cell path 10 mm (UV - orange trace, ECD - blue trace)

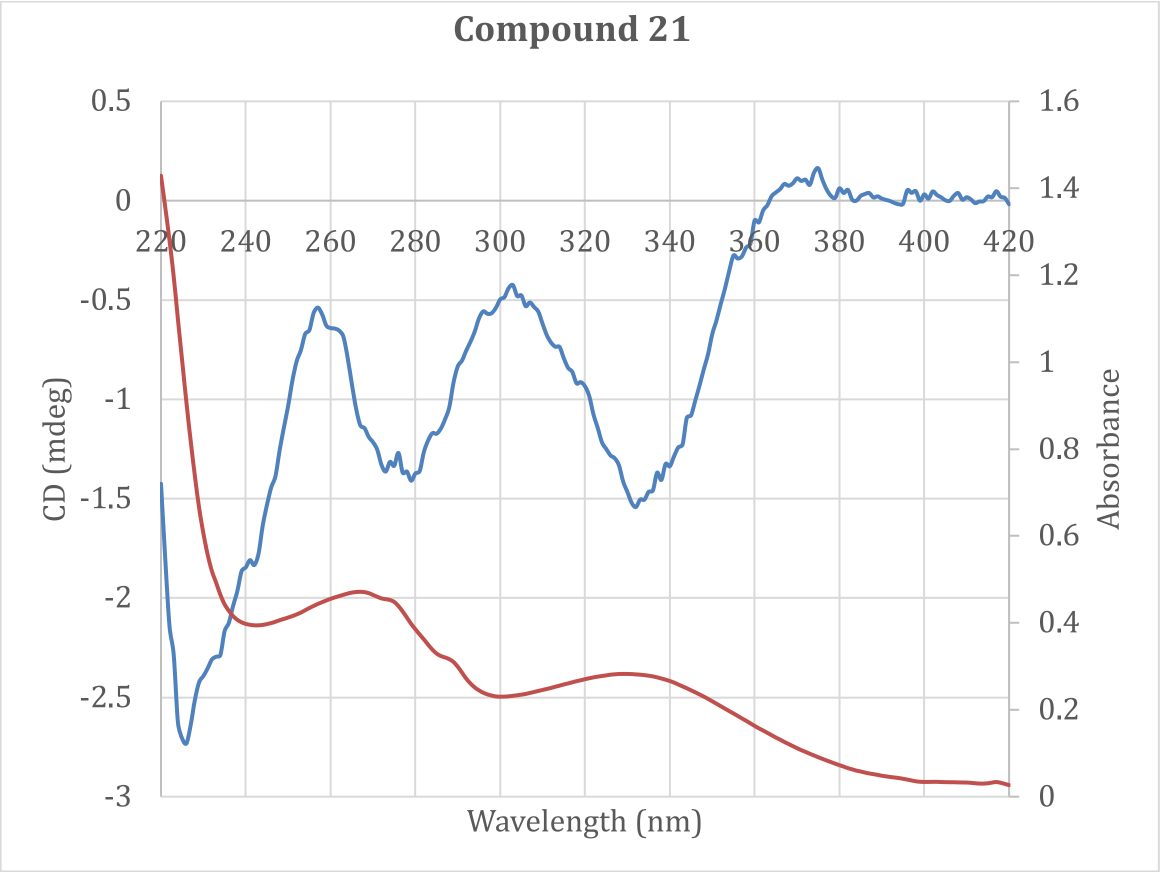

Figure S11. ECD and UV spectra of **21** (*c* 0.07 mM, MeOH)
Cell path 10 mm (UV - orange trace, ECD - blue trace)

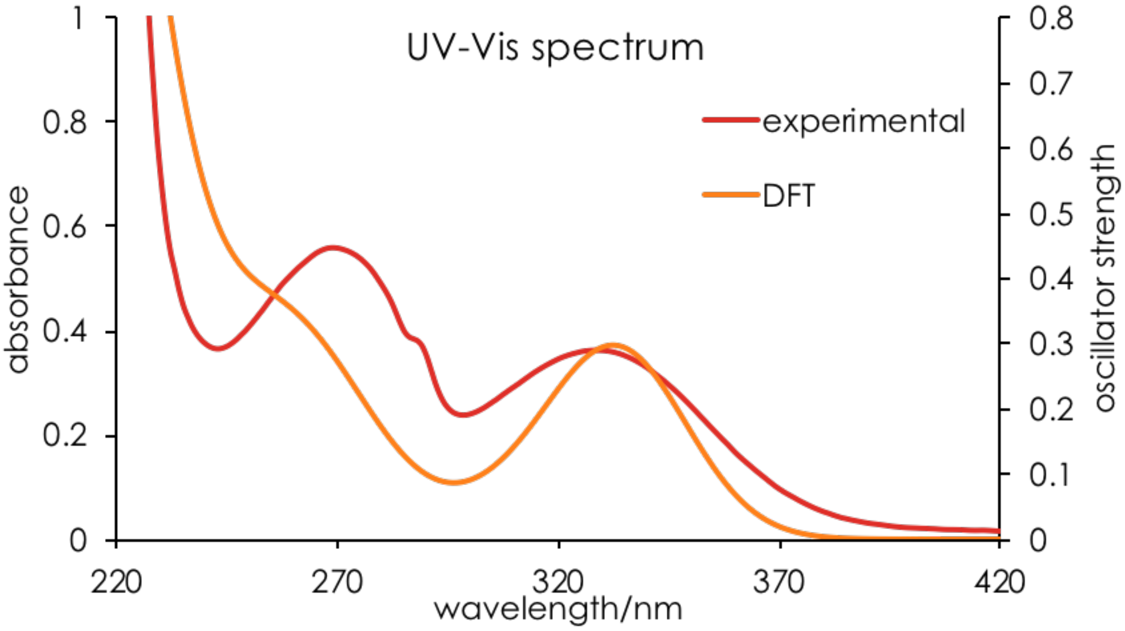

Figure S12. UV spectrum of **16** (*c* 0.07 mM, MeOH) compared to the calculated spectrum.

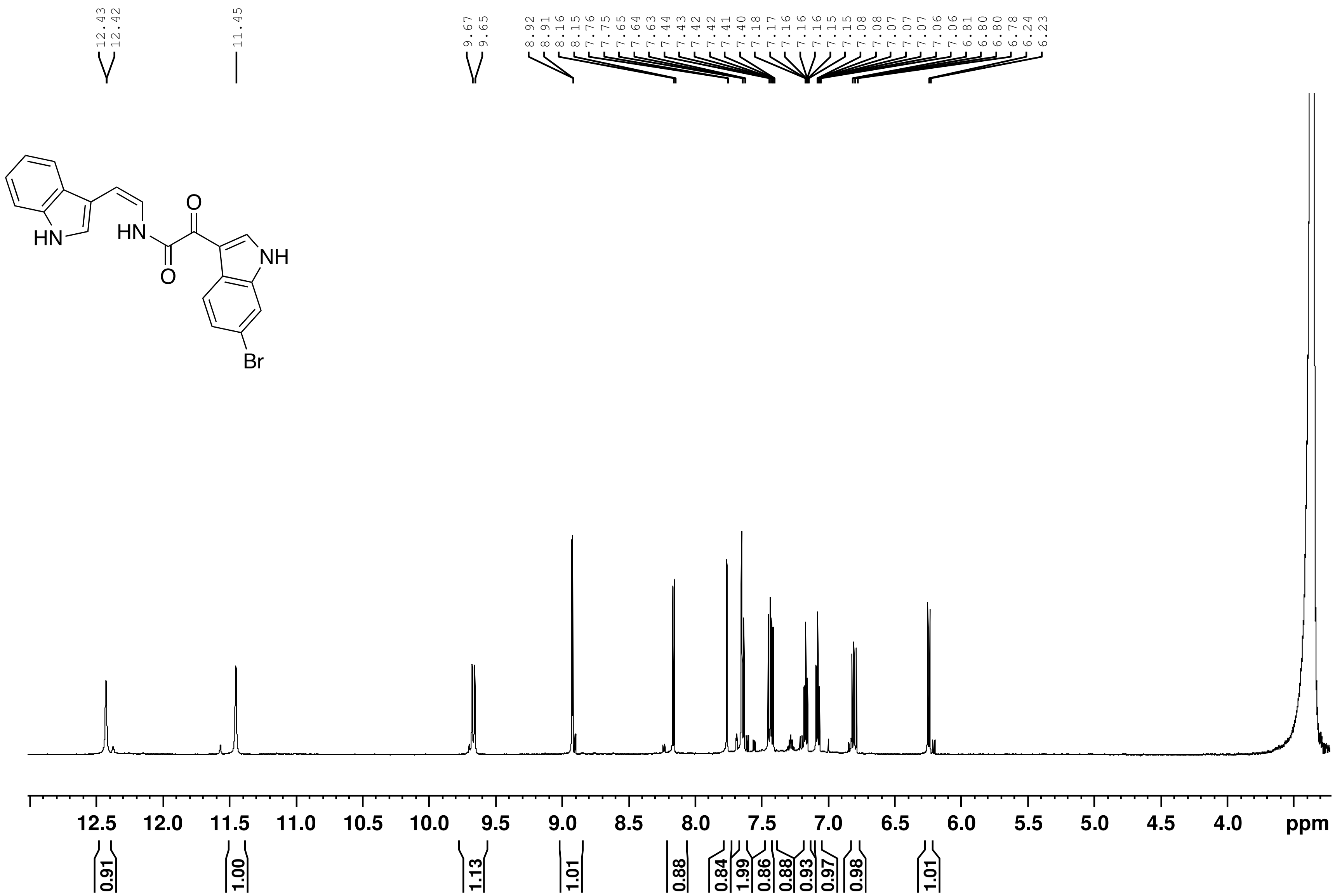

Figure S13. ^{1}H NMR spectrum (600 MHz) of (*Z*)-coscinamide D (**1**) in DMSO-d_6

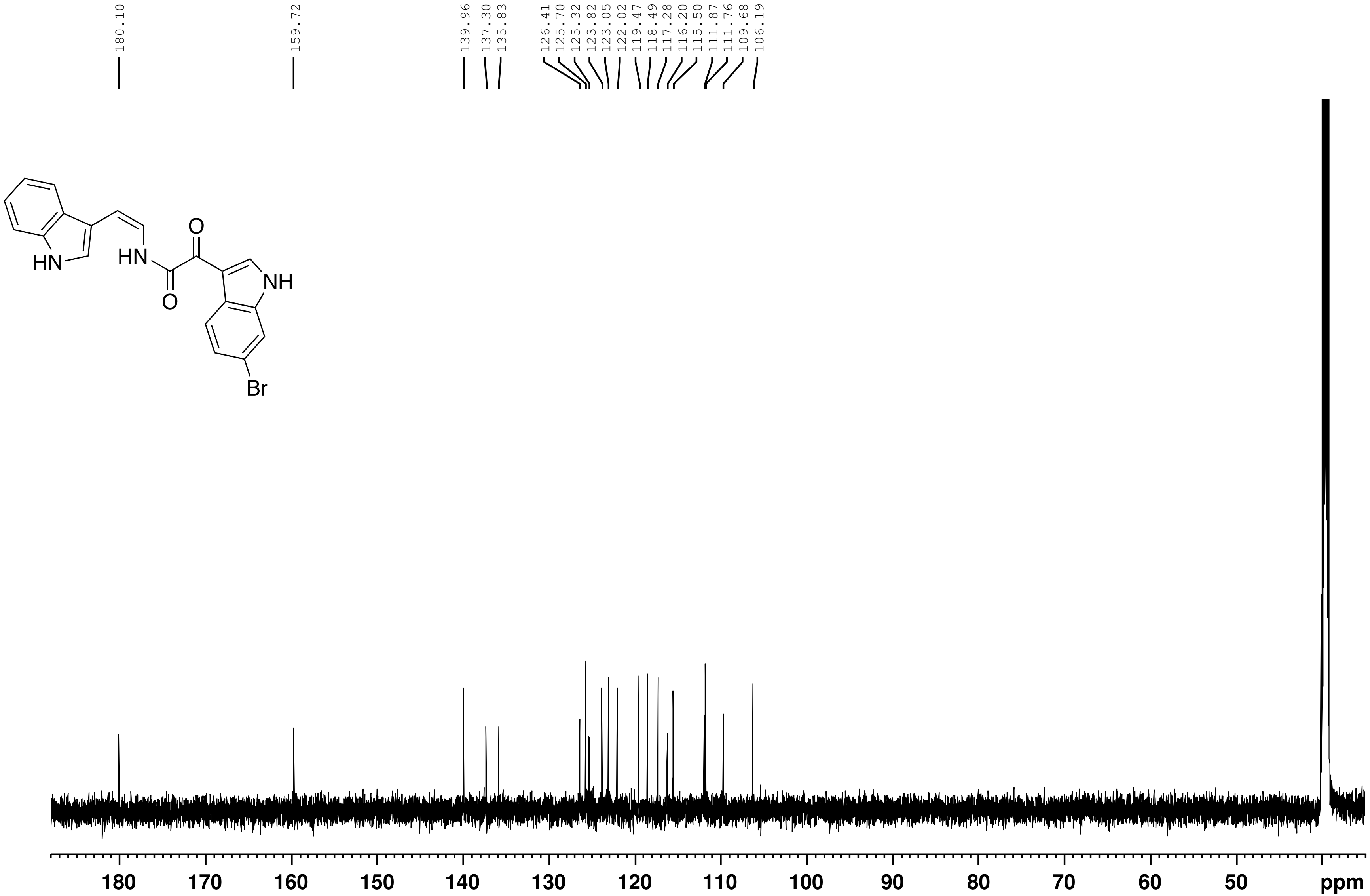

Figure S14. ^{13}C NMR spectrum (150 MHz) of (*Z*)-coscinamide D (**1**) in DMSO-d_6

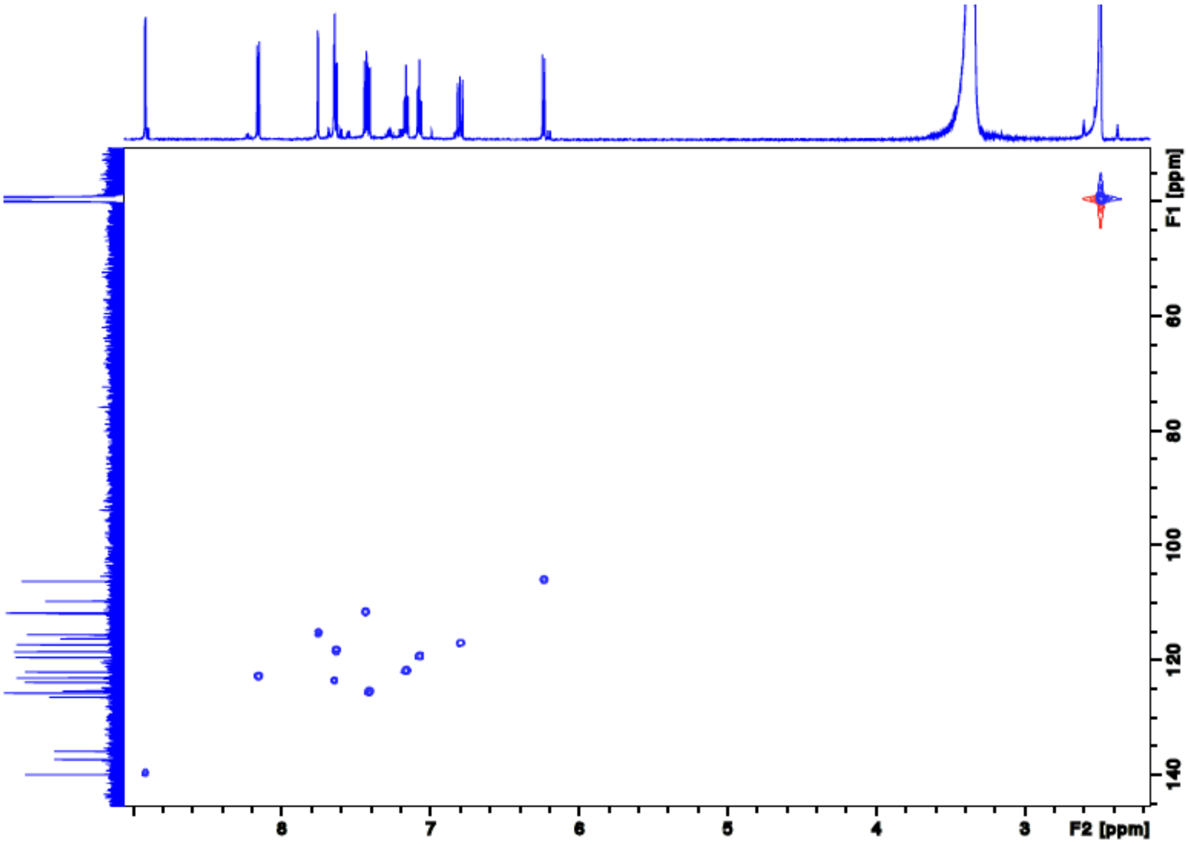

Figure S15. ^1H-^{13}C HSQC spectrum (600 MHz) of (*Z*)-coscinamide D (**1**) in DMSO-d_6

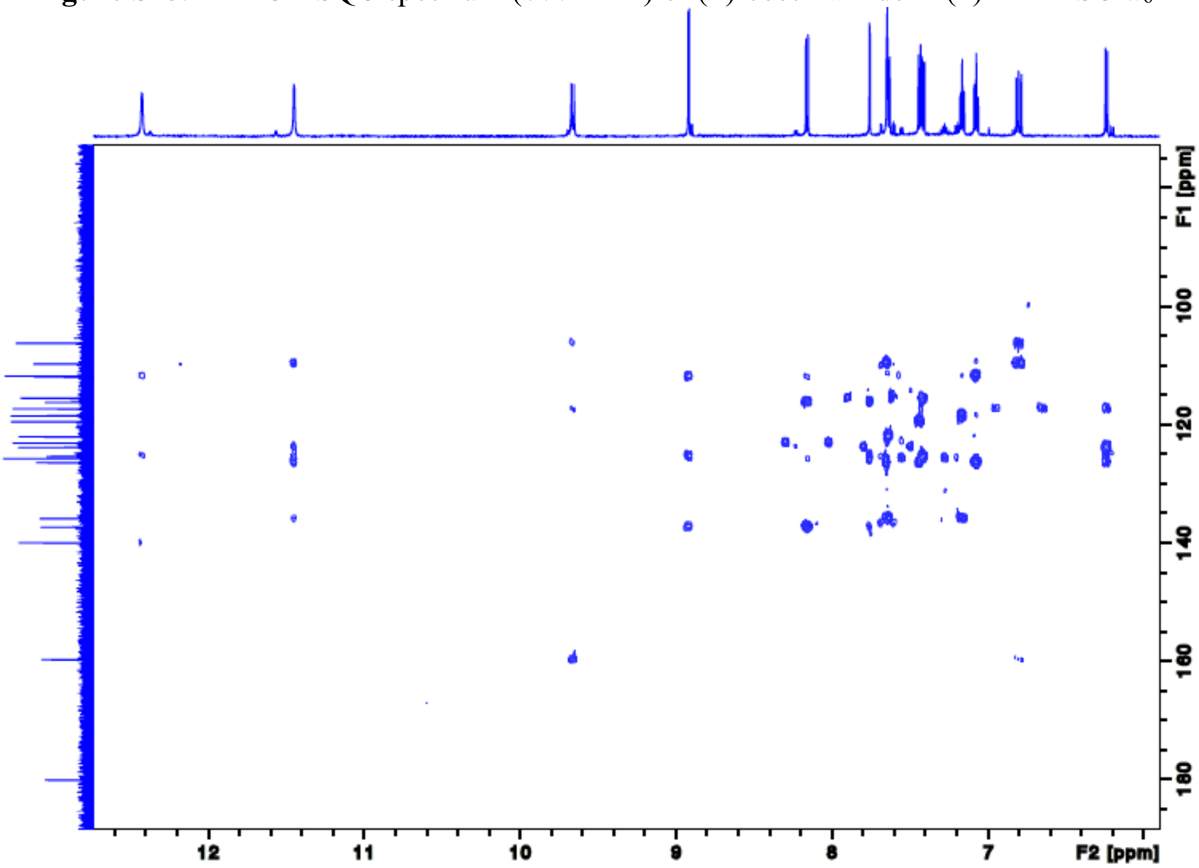

Figure S16. ^1H-^{13}C HMBC spectrum (600 MHz) of (*Z*)-coscinamide D (**1**) in DMSO-d_6

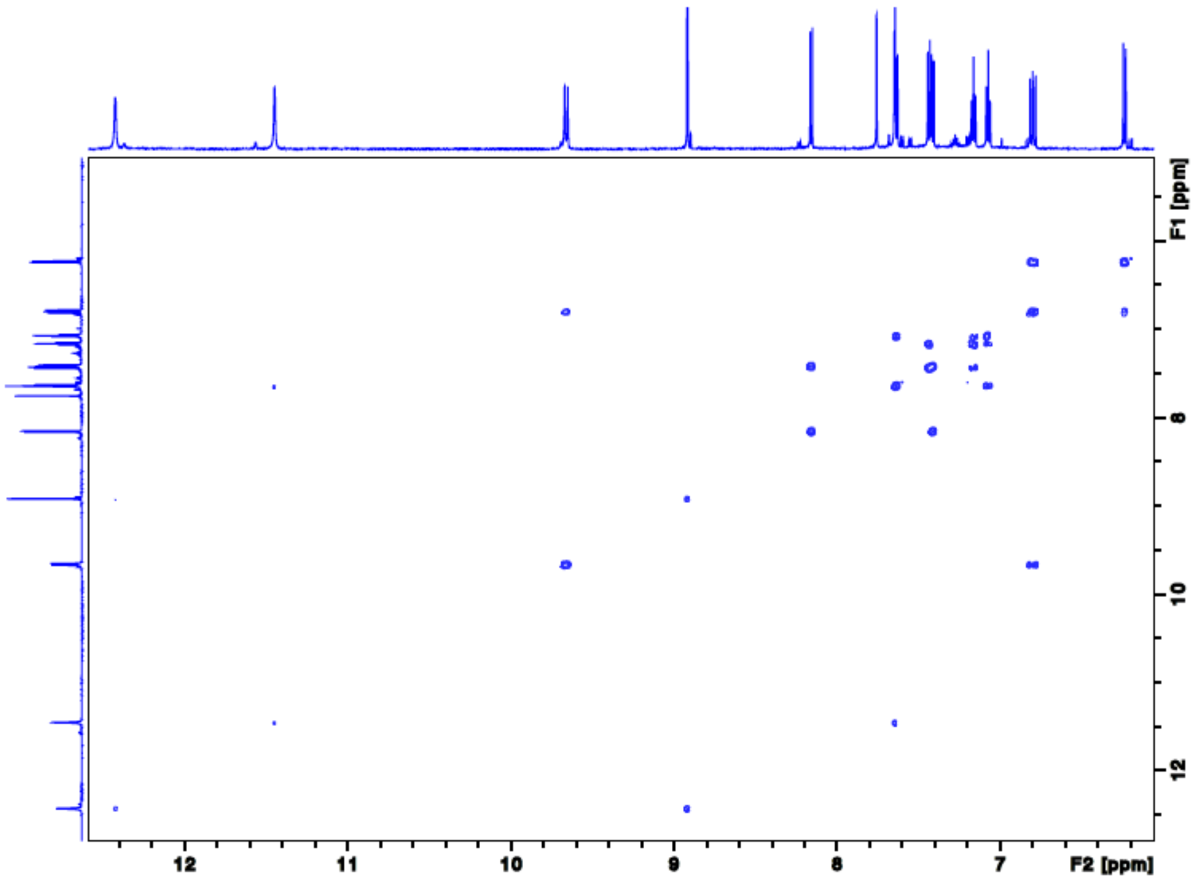

Figure S17. ^{1}H-^{1}H COSY spectrum (600 MHz) of (*Z*)-coscinamide D (**1**) in DMSO-d_6

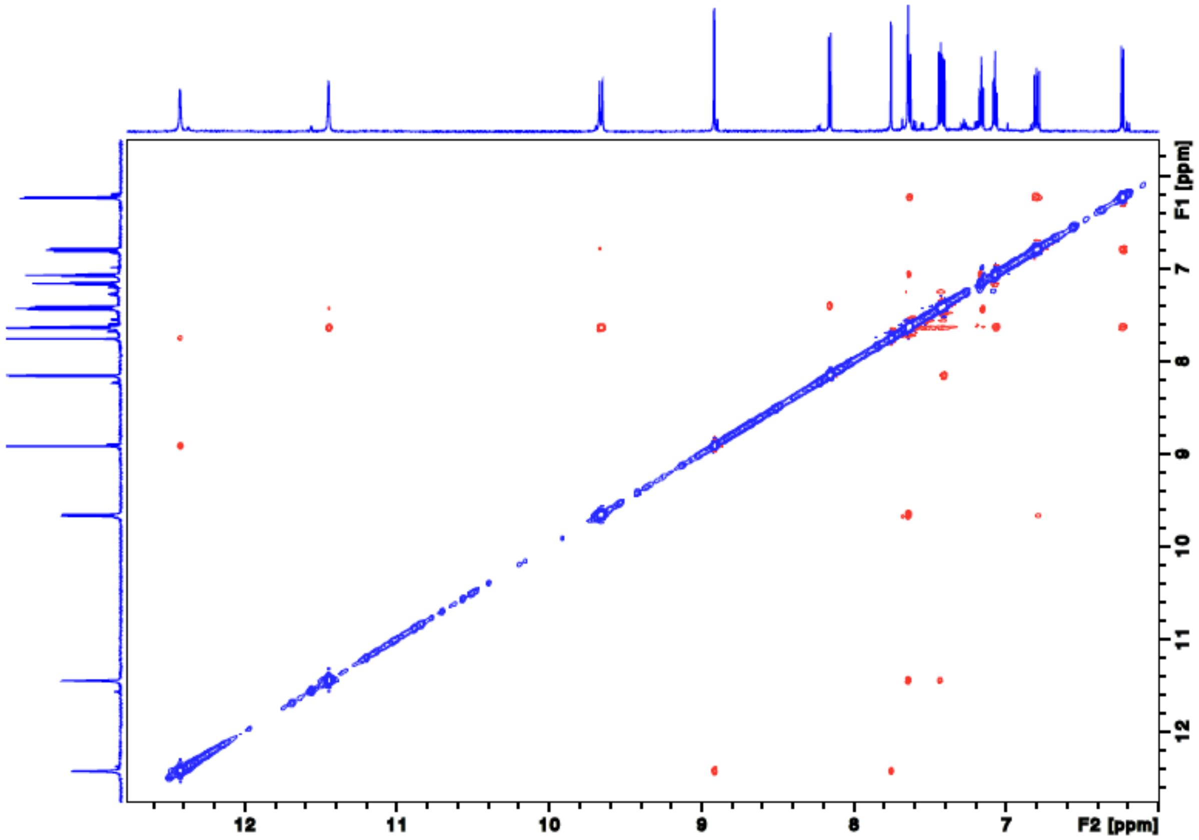

Figure S18. ^{1}H-^{1}H ROESY spectrum (600 MHz) of (*Z*)-coscinamide D (**1**) in DMSO-d_6

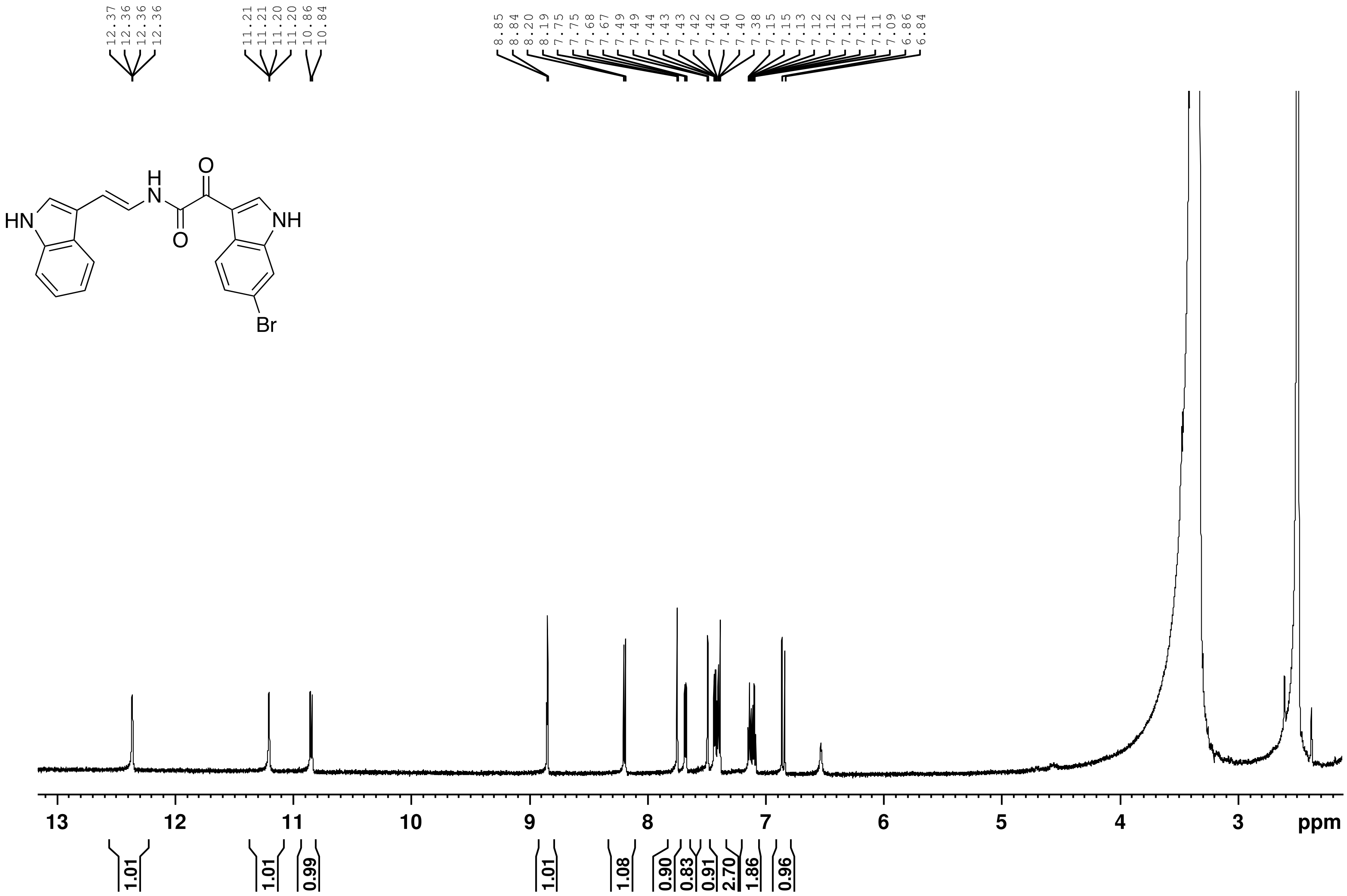

Figure S19. ^{1}H NMR spectrum (600 MHz) of coscinamide D (**2**) in DMSO-d_6

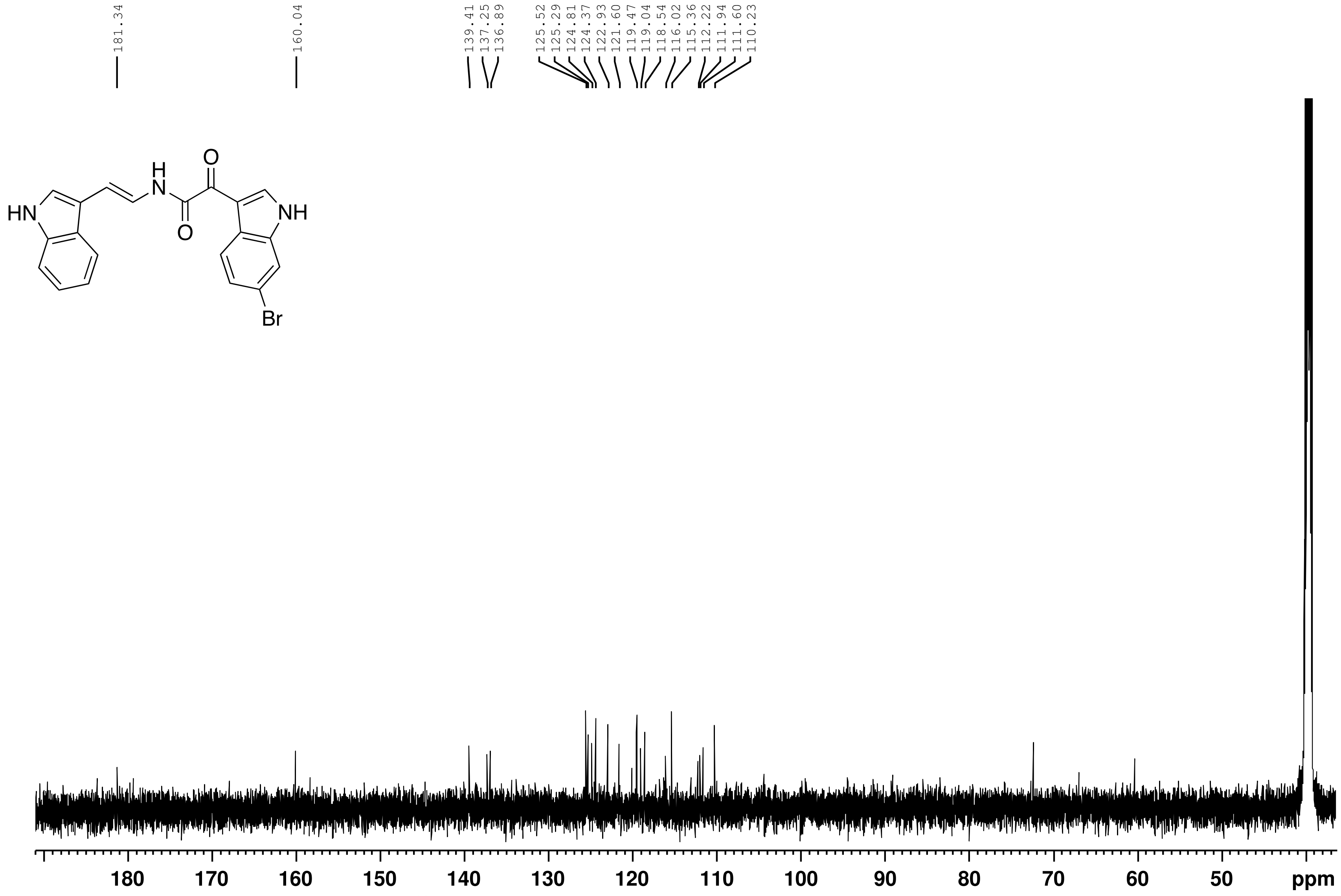

Figure S20. ^{13}C NMR spectrum (150 MHz) of coscinamide D (**2**) in DMSO-d_6

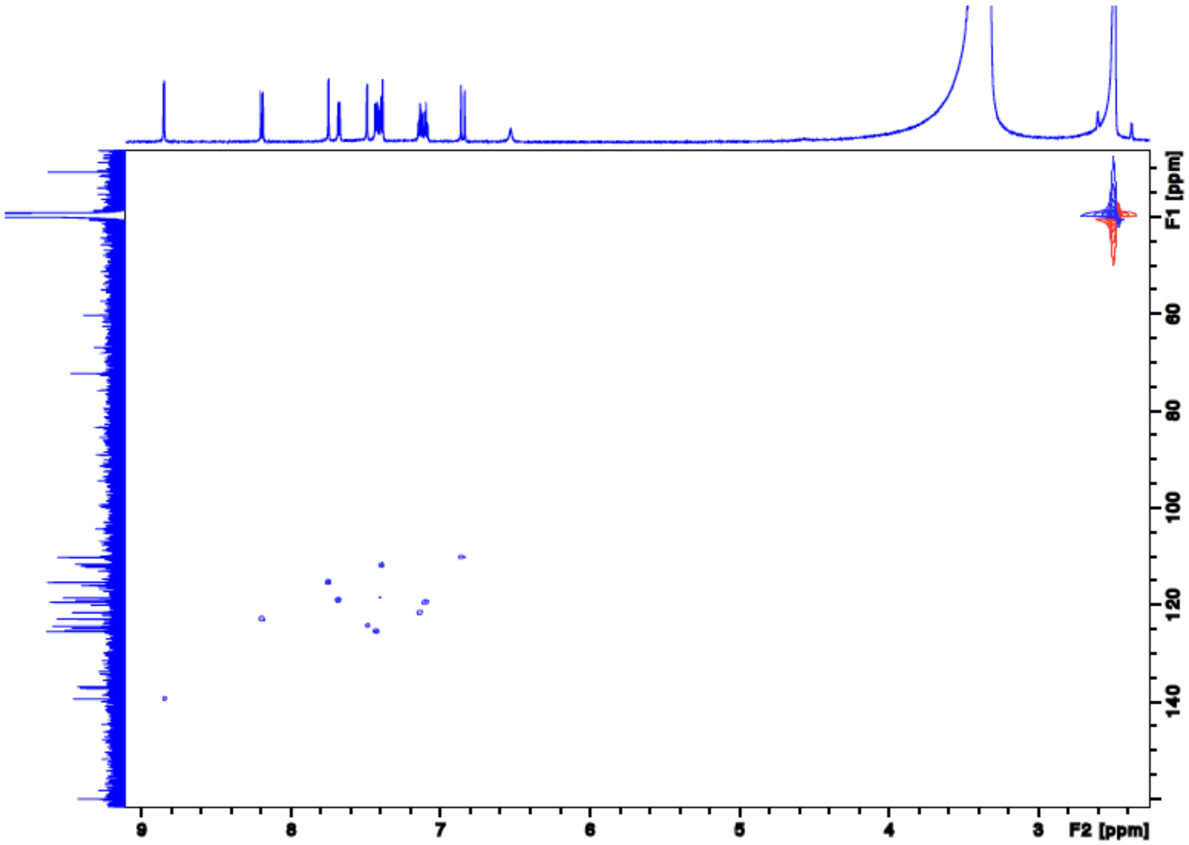

Figure S21. ^{1}H-^{13}C HSQC spectrum (600 MHz) of coscinamide D (**2**) in DMSO-d_6

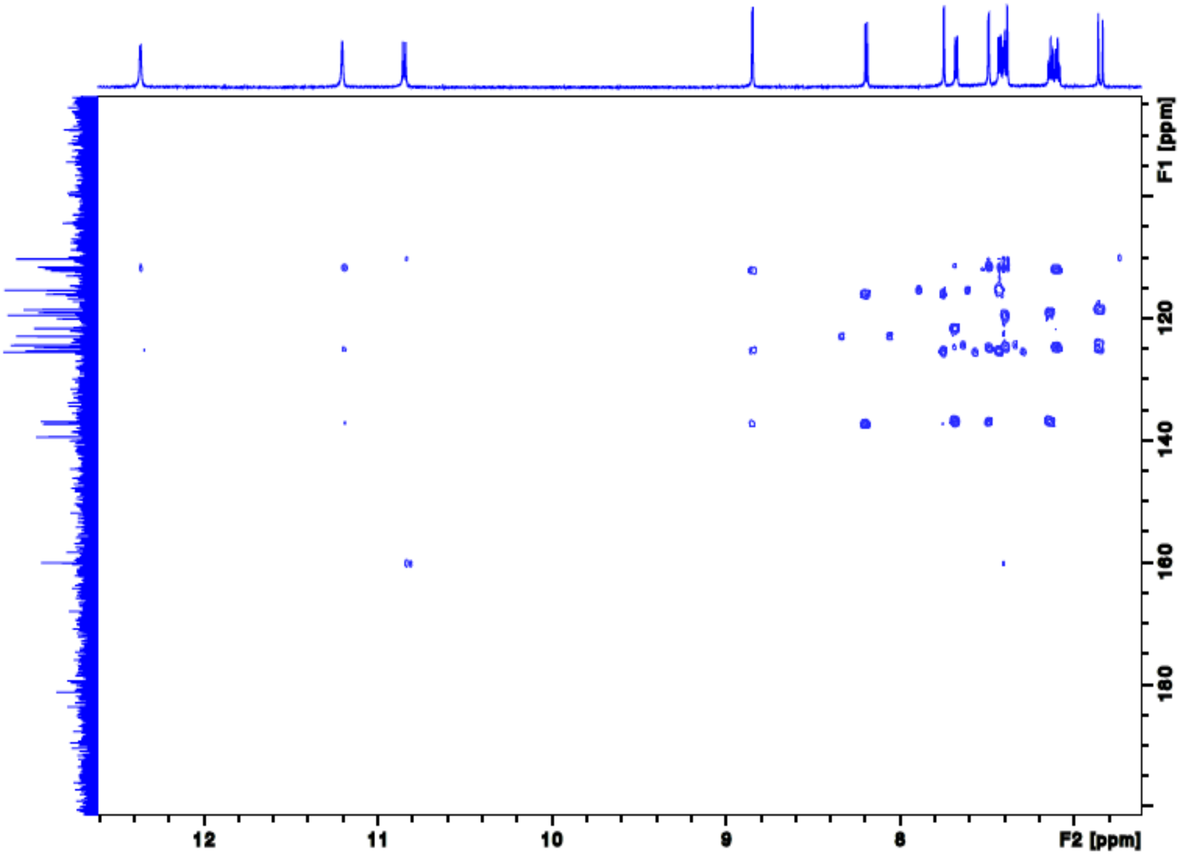

Figure S22. ^{1}H-^{13}C HMBC spectrum (600 MHz) of coscinamide D (**2**) in DMSO-d_6

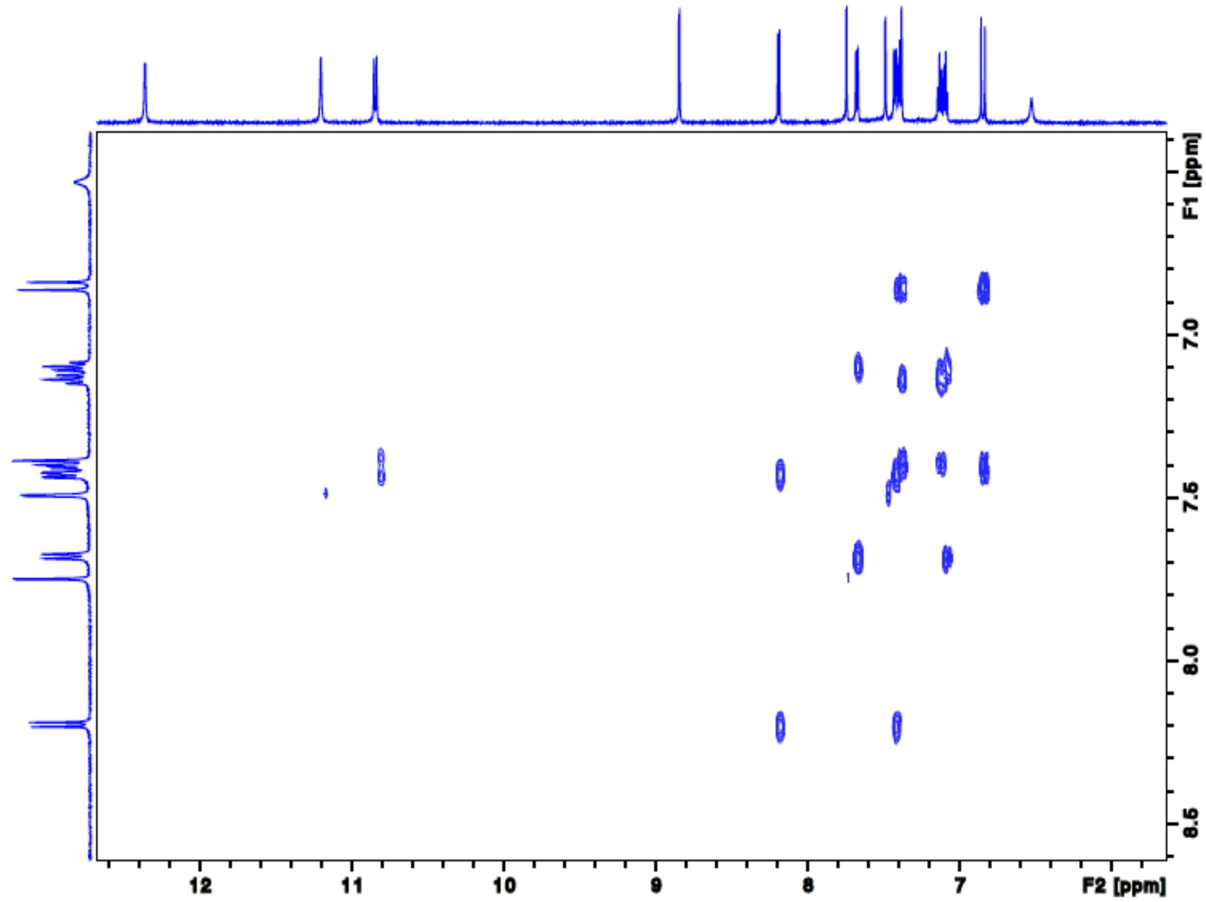

Figure S23. ^{1}H-^{1}H COSY spectrum (600 MHz) of coscinamide D (**2**) in DMSO-d_6

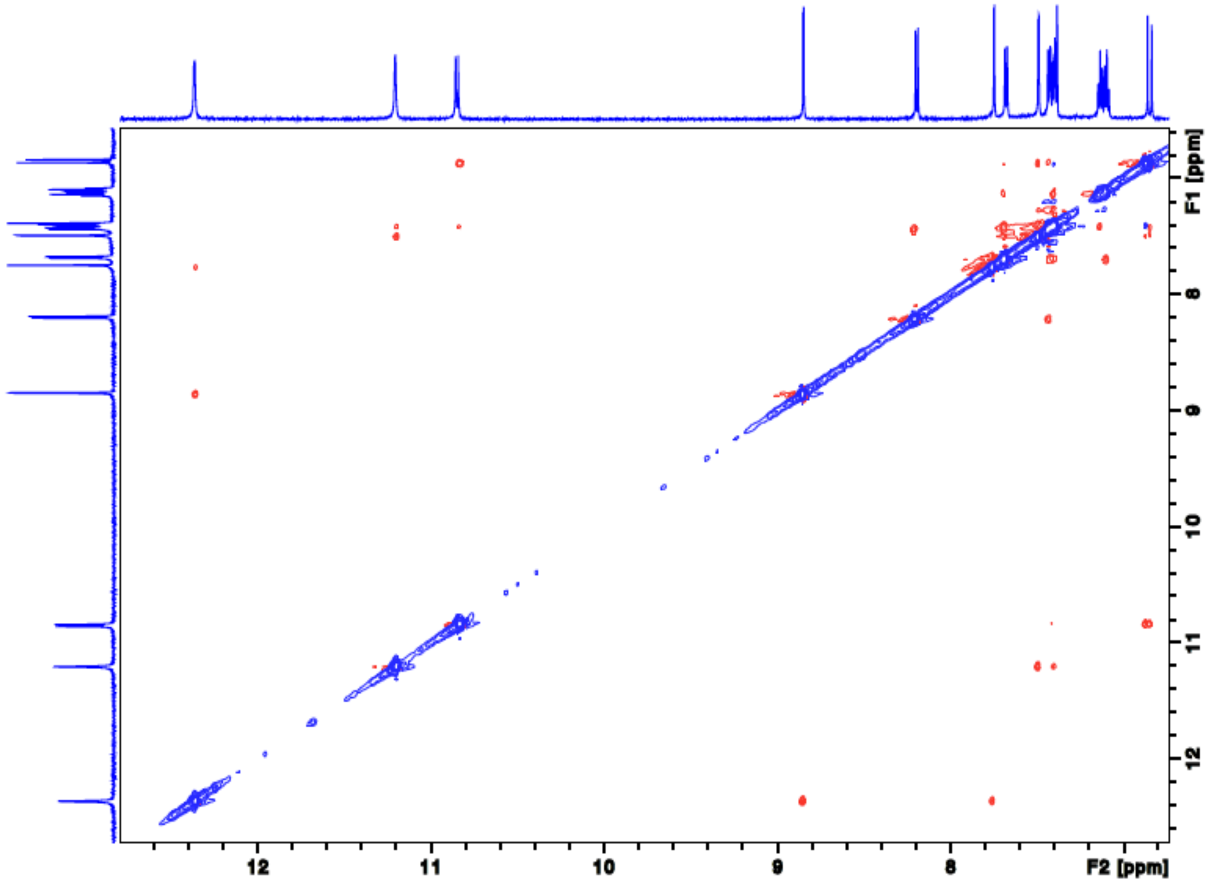

Figure S24. ^{1}H-^{1}H ROESY spectrum (600 MHz) of coscinamide D (**2**) in DMSO-*d*

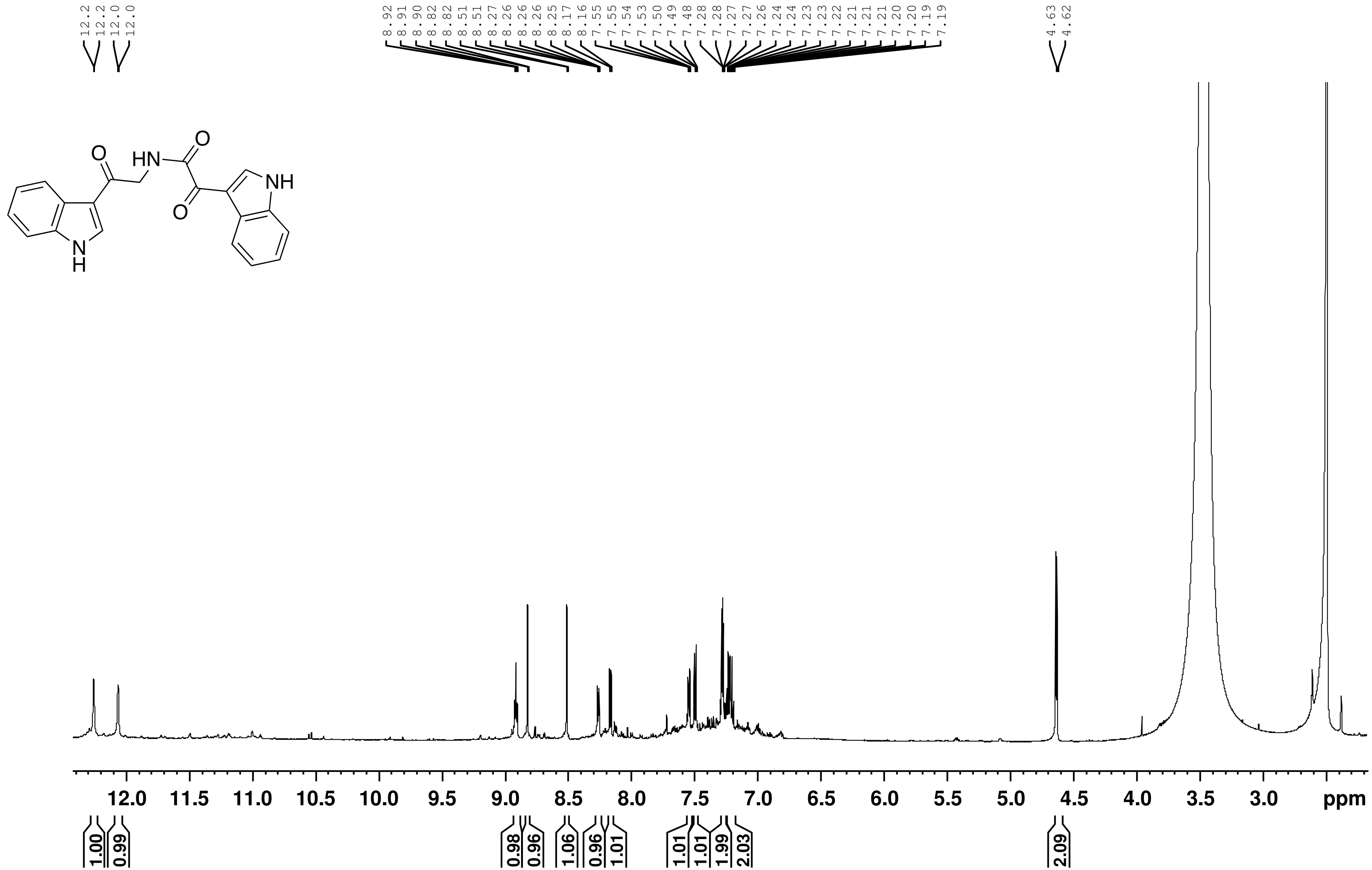

Figure S25. ^{1}H NMR spectrum (600 MHz) of lamellomorphamide A (**3**) in DMSO-d_6

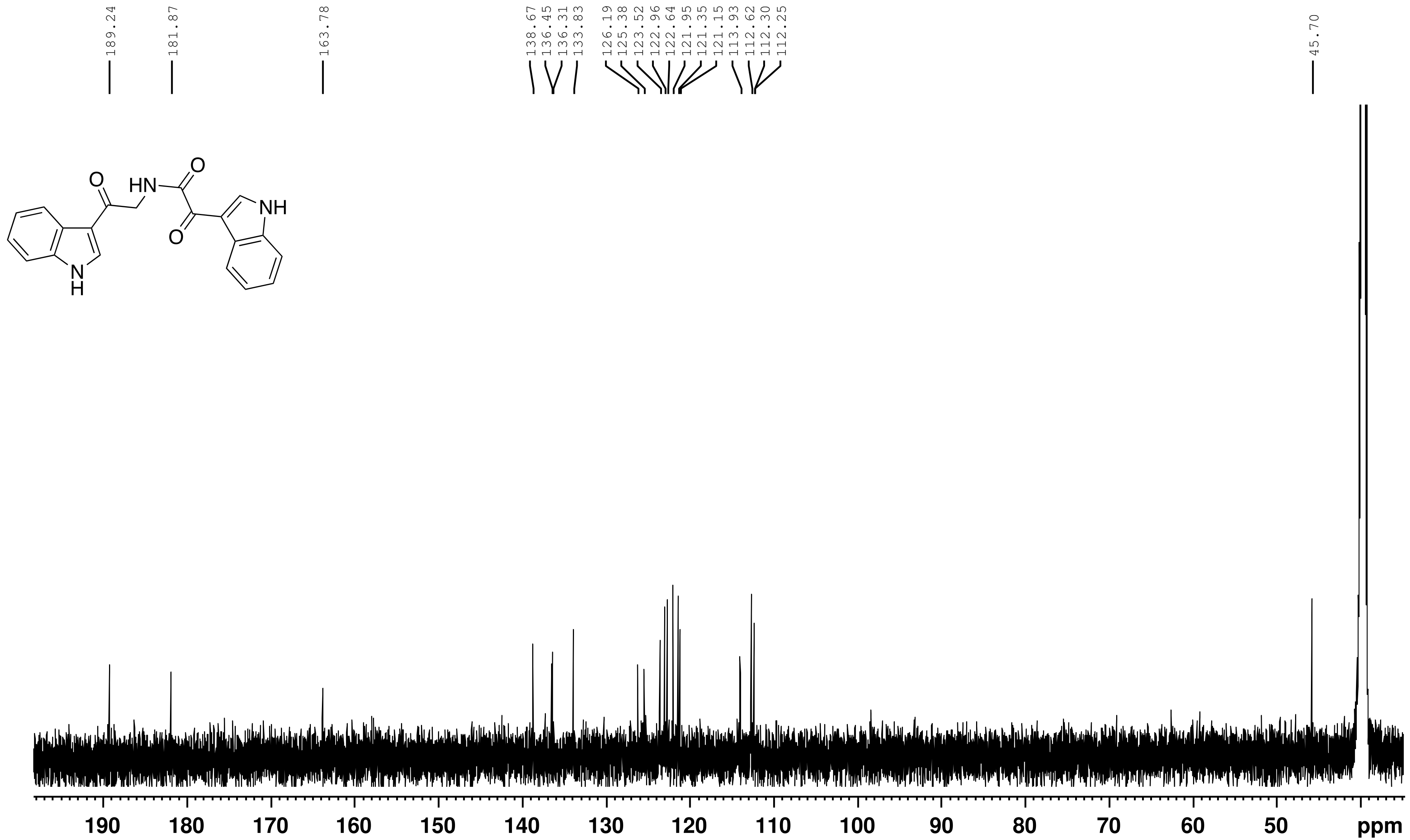

Figure S26. ^{13}C NMR spectrum (150 MHz) of lamellomorphamide A (**3**) in DMSO-d_6

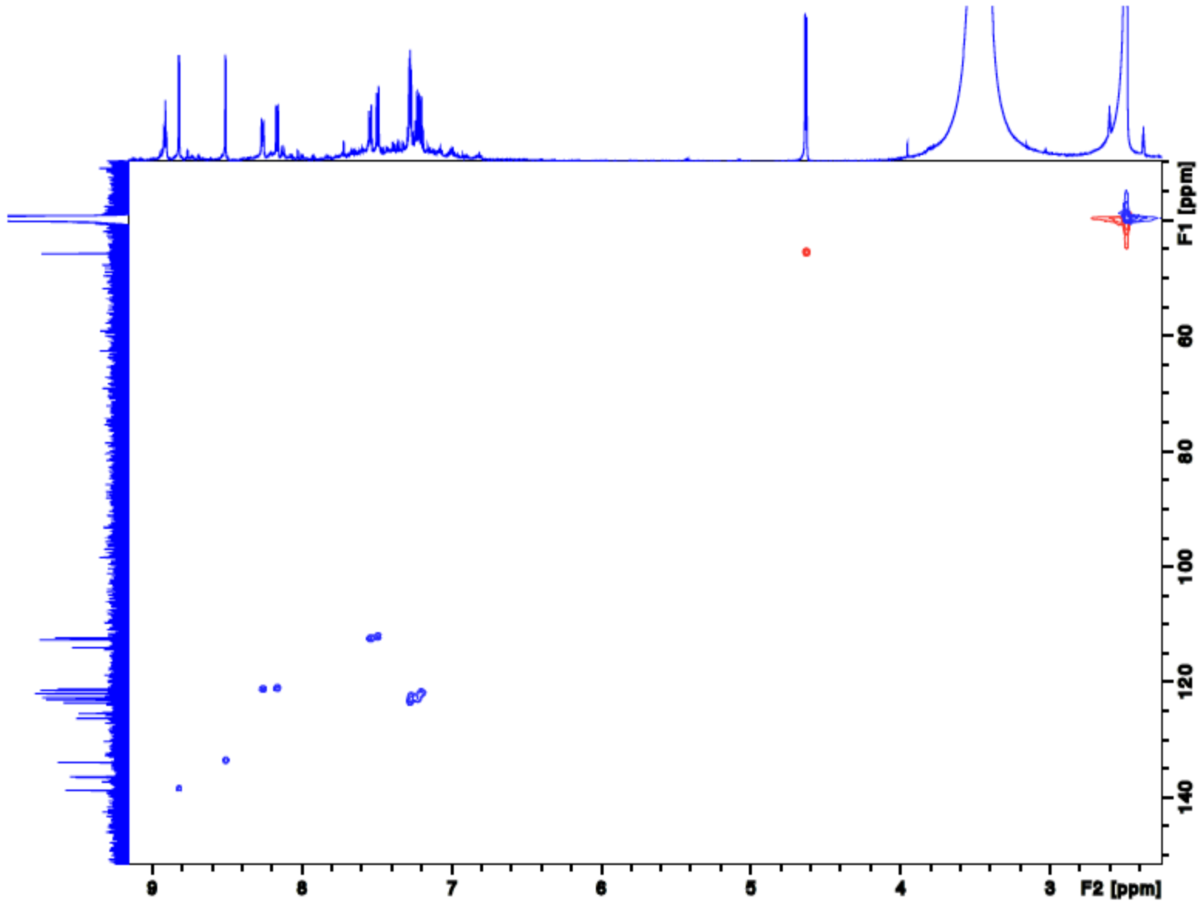

Figure S27. ^{1}H- ^{13}C HSQC spectrum (600 MHz) of lamellomorphamide A (**3**) in DMSO-d_6

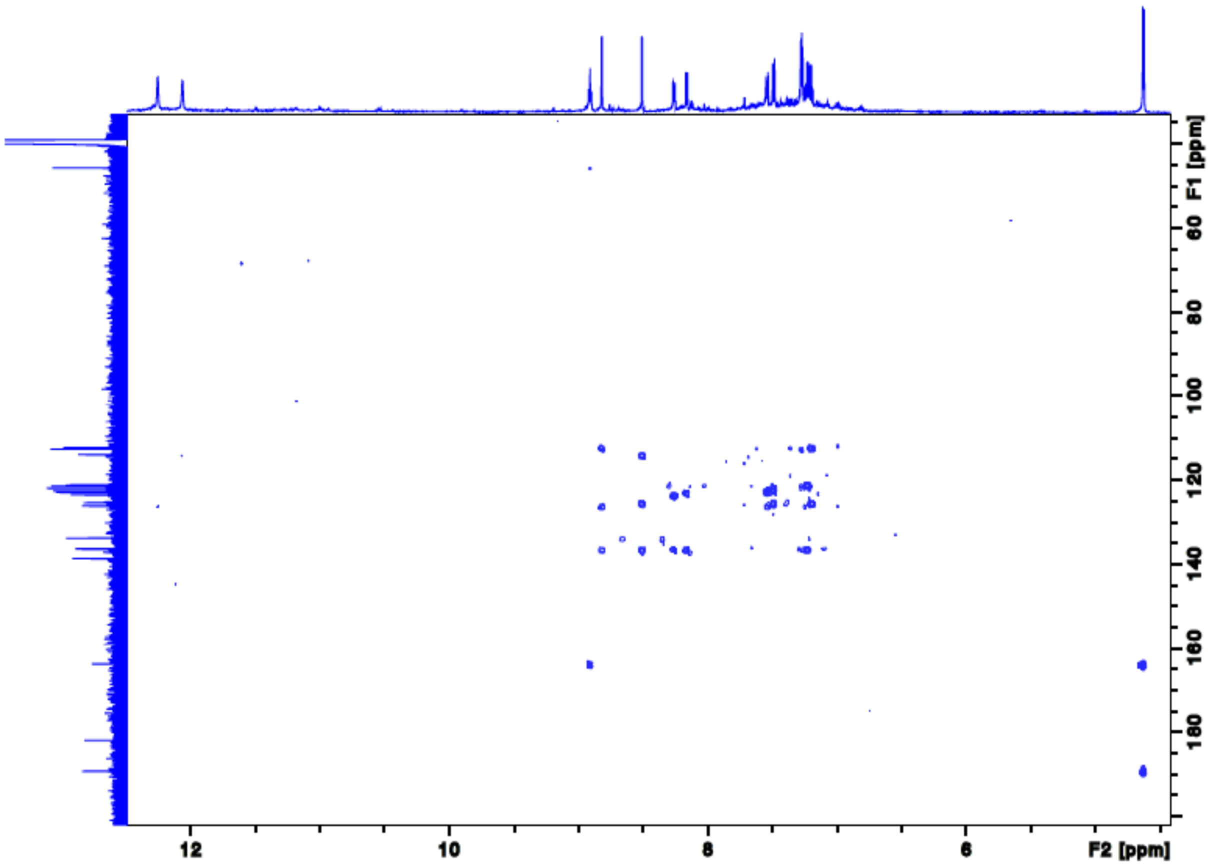

Figure S28. ^{1}H- ^{13}C HMBC spectrum (600 MHz) of lamellomorphamide A (**3**) in DMSO-d_6

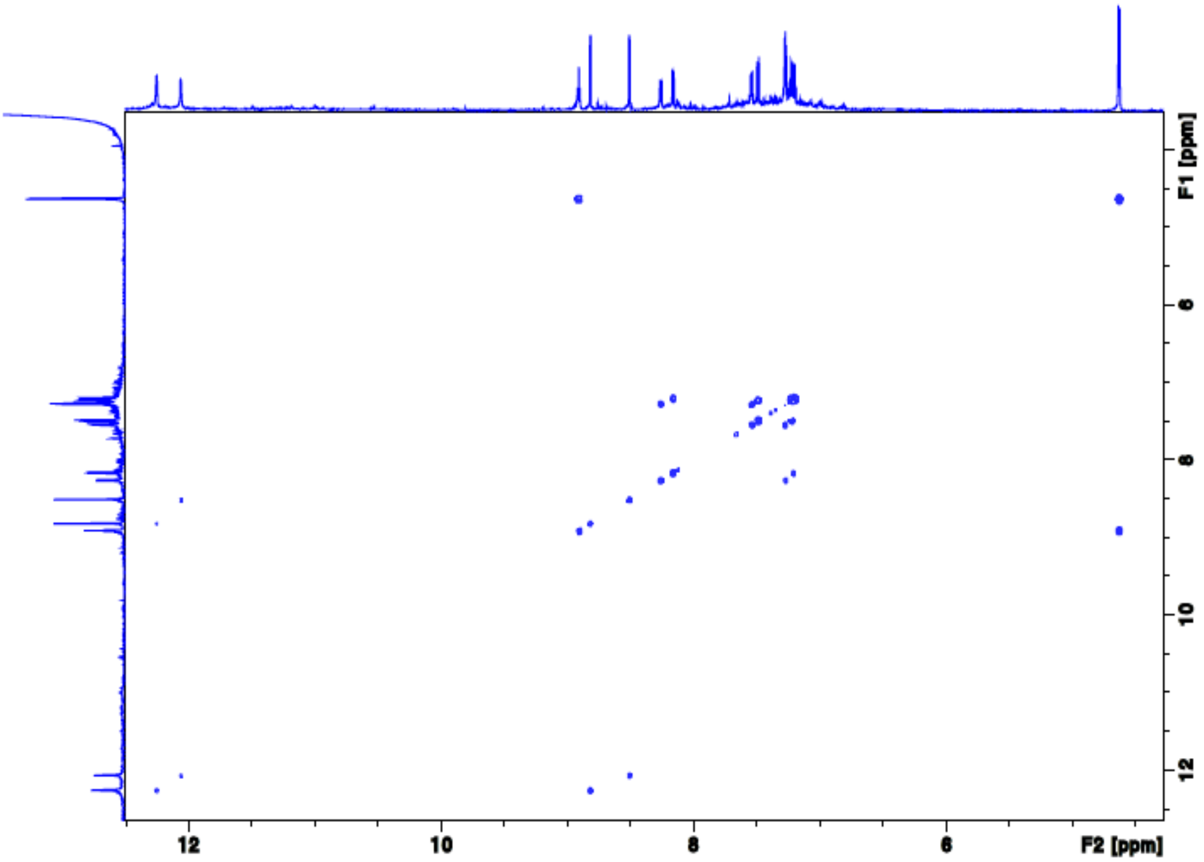

Figure S29. ^{1}H-^{1}H COSY spectrum (600 MHz) of lamellomorphamide A (**3**) in DMSO-d_6

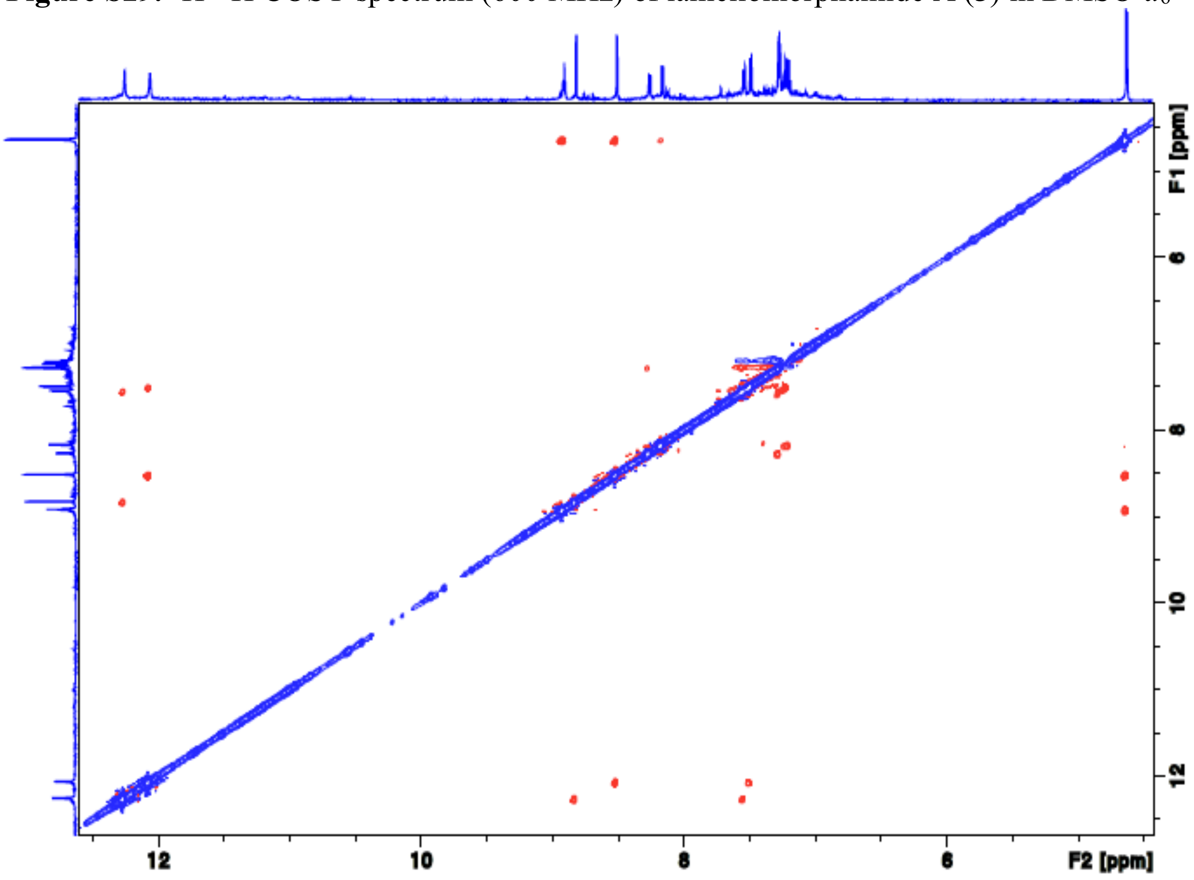

Figure S30. ^{1}H-^{1}H ROESY spectrum (600 MHz) of lamellomorphamide A (**3**) in DMSO-

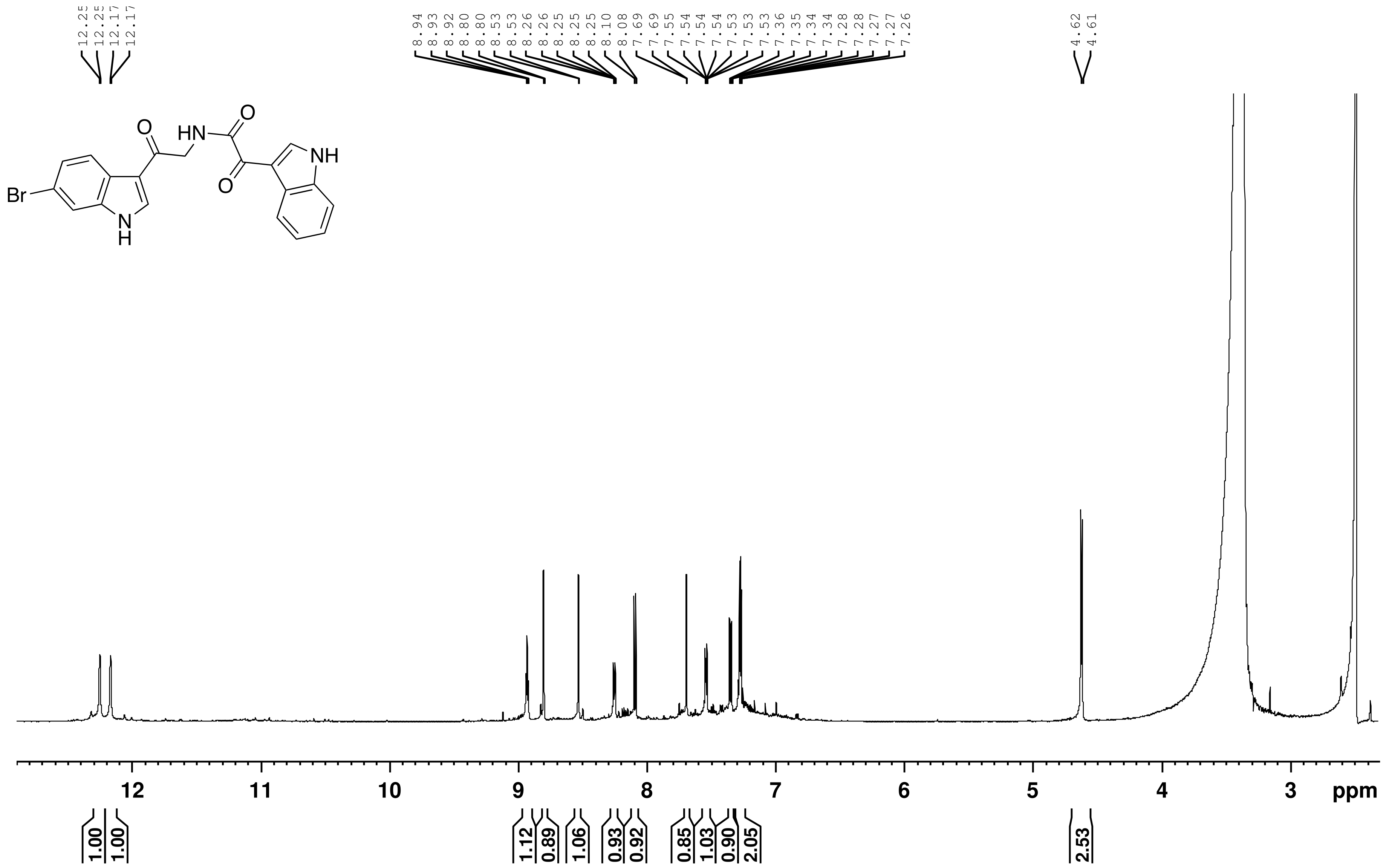

Figure S31. ^{1}H NMR spectrum (600 MHz) of lamellomorphamide B (**4**) in DMSO-d_6

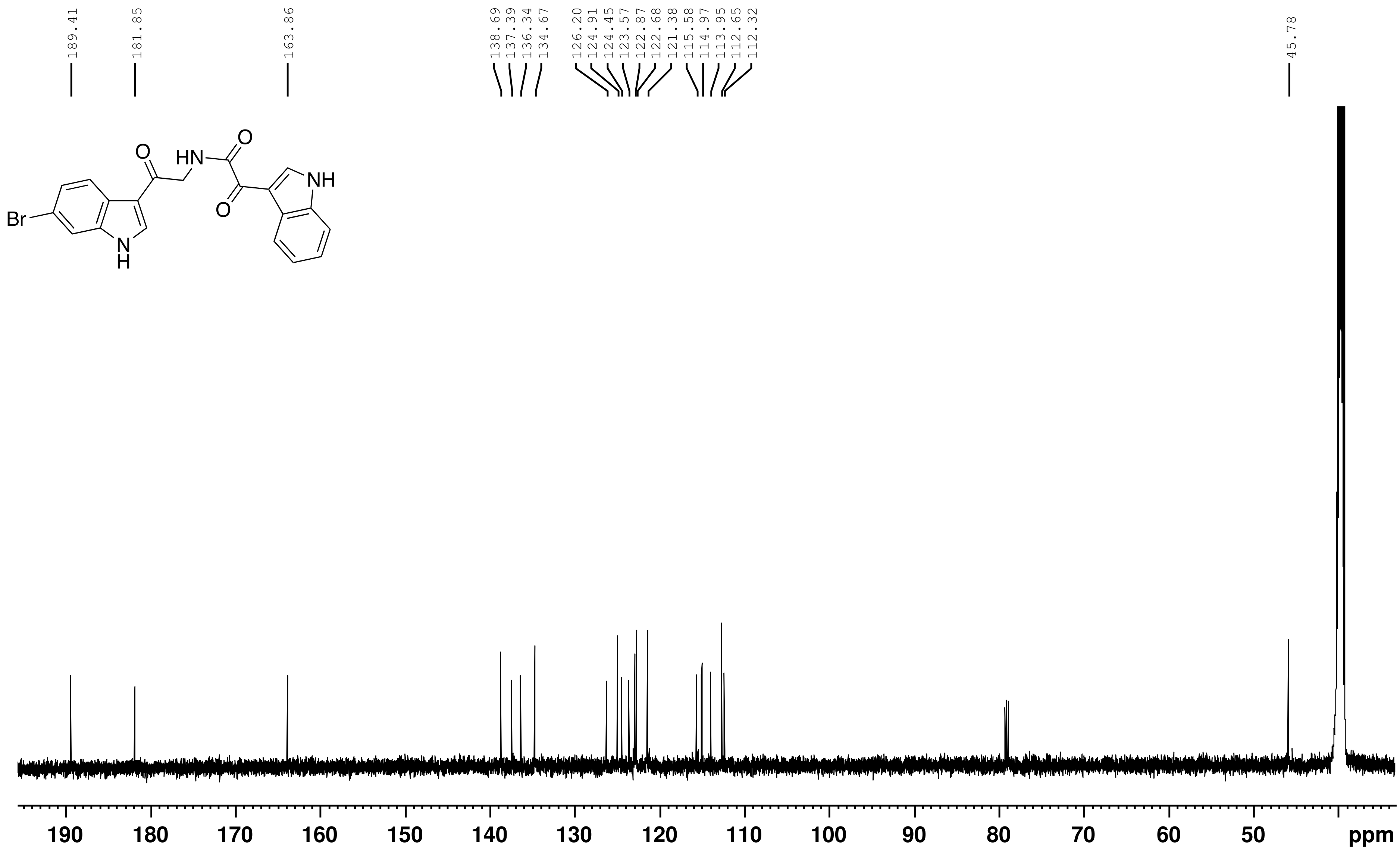

Figure S32. ^{13}C NMR spectrum (150 MHz) of lamellomorphamide B (**4**) in DMSO-d_6

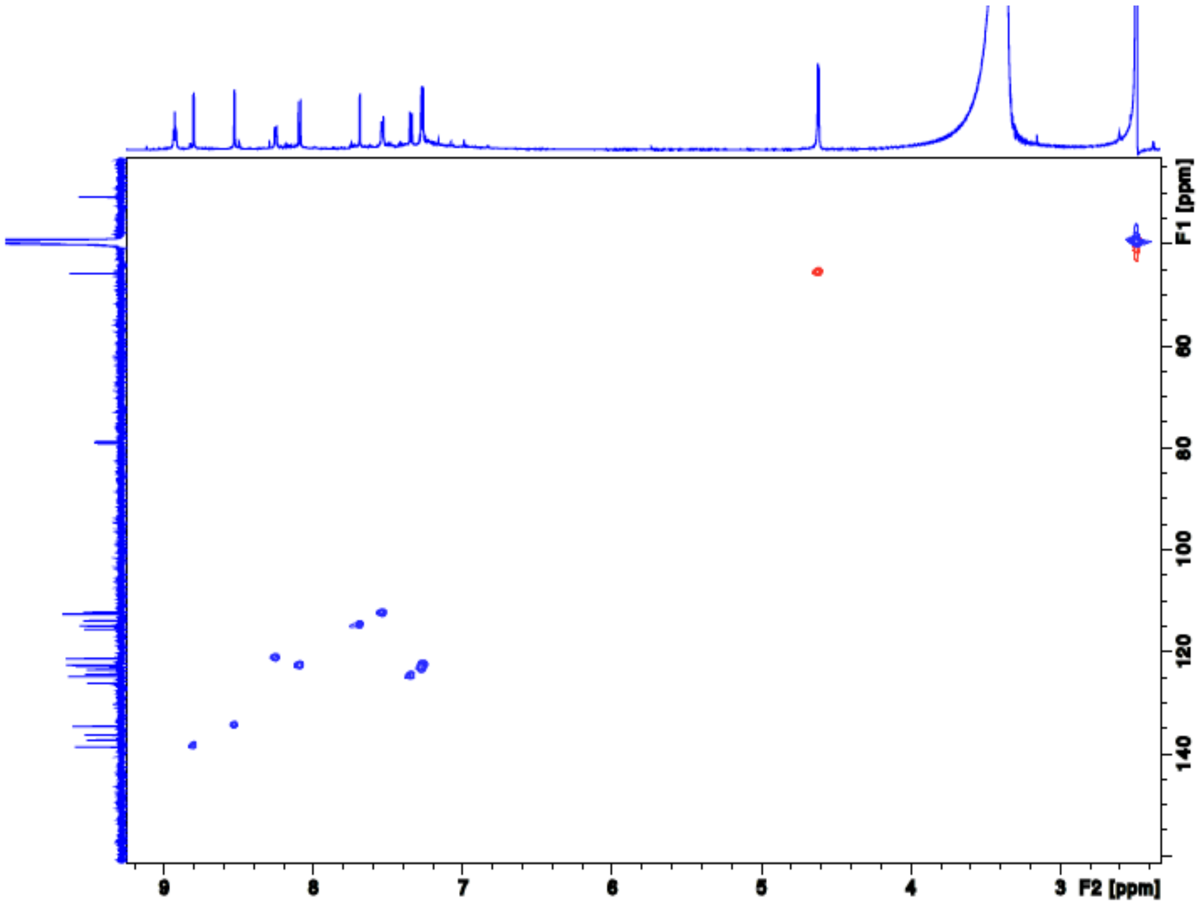

Figure S33. ^{1}H- ^{13}C HSQC spectrum (600 MHz) of lamellomorphamide B (**4**) in DMSO-d_6

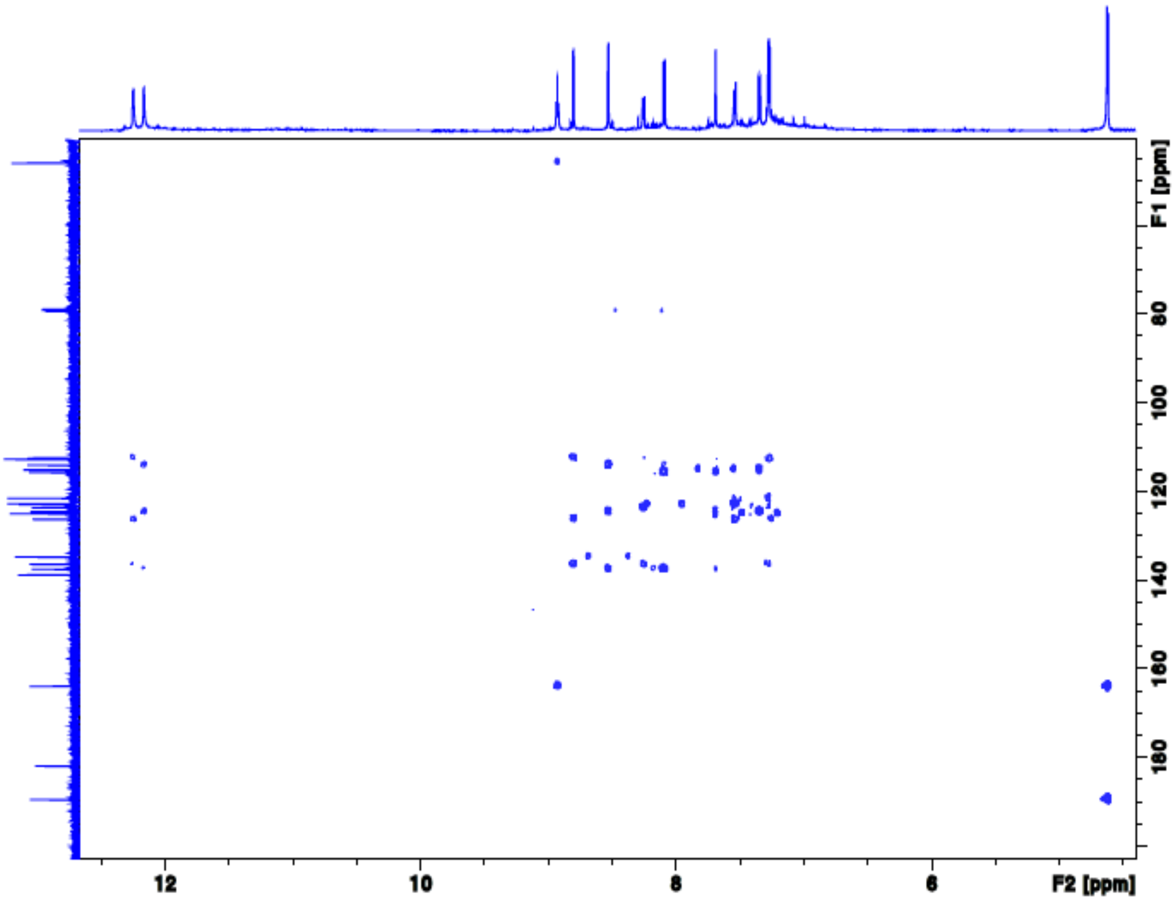

Figure S34. ^{1}H- ^{13}C HMBC spectrum (600 MHz) of lamellomorphamide B (**4**) in DMSO-d_6

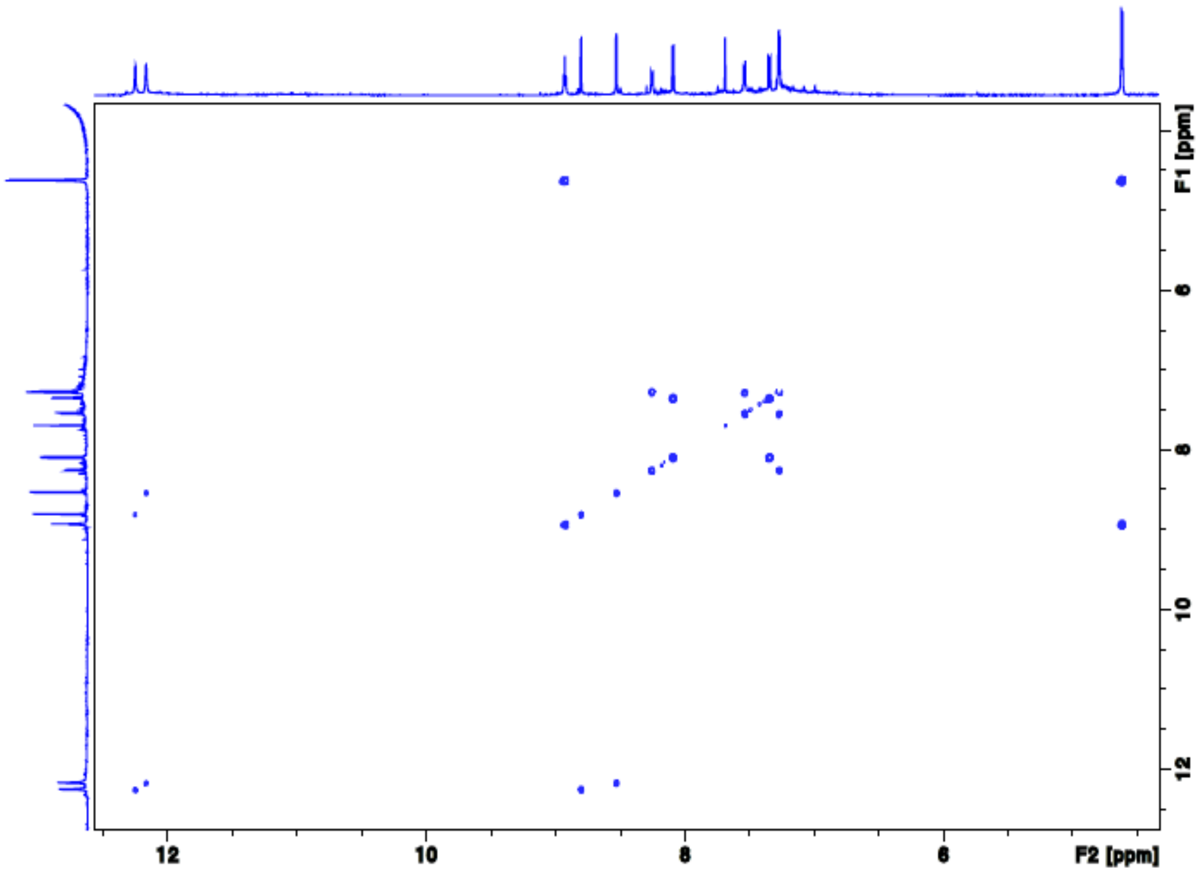

Figure S35. ^{1}H-^{1}H COSY spectrum (600 MHz) of lamellomorphamide B (**4**) in DMSO-d_6

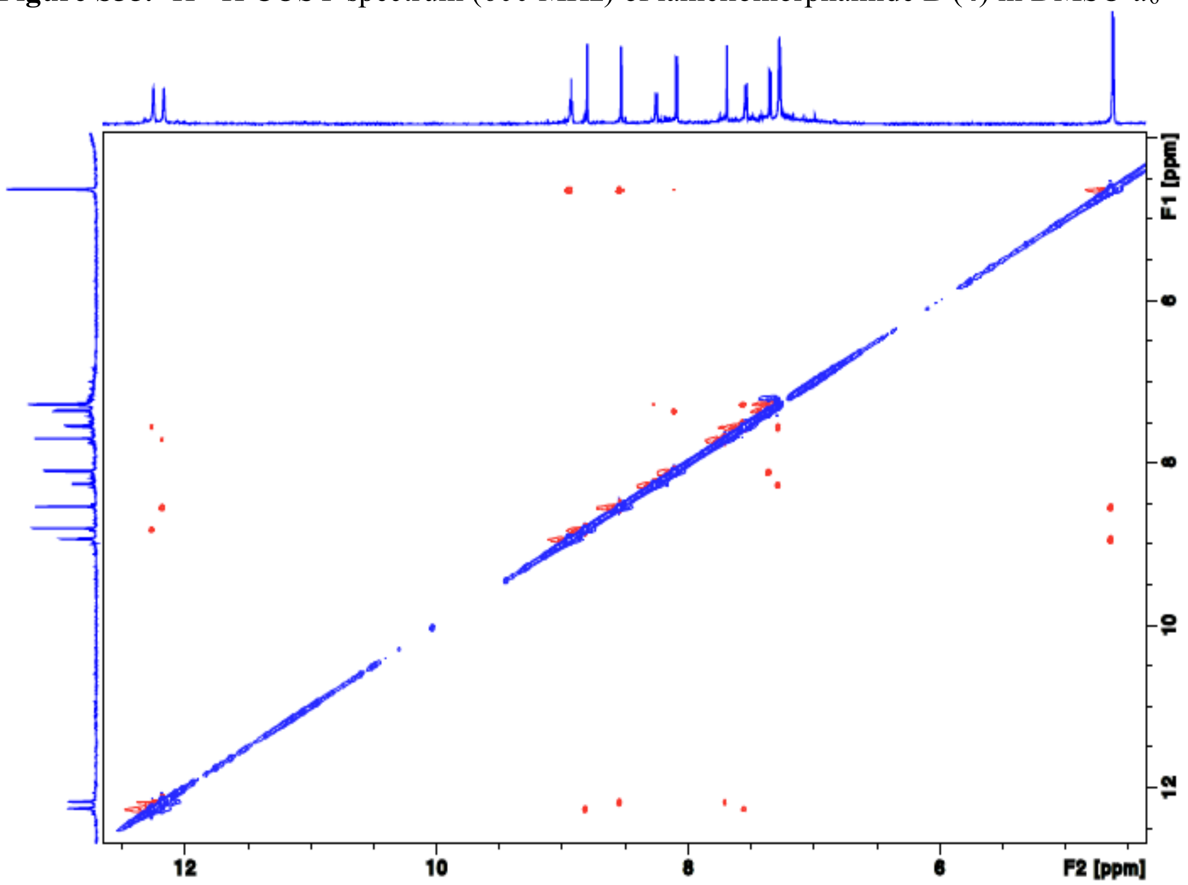

Figure S36. ^{1}H-^{1}H ROESY spectrum (600 MHz) of lamellomorphamide B (**4**) in DMSO-d_6

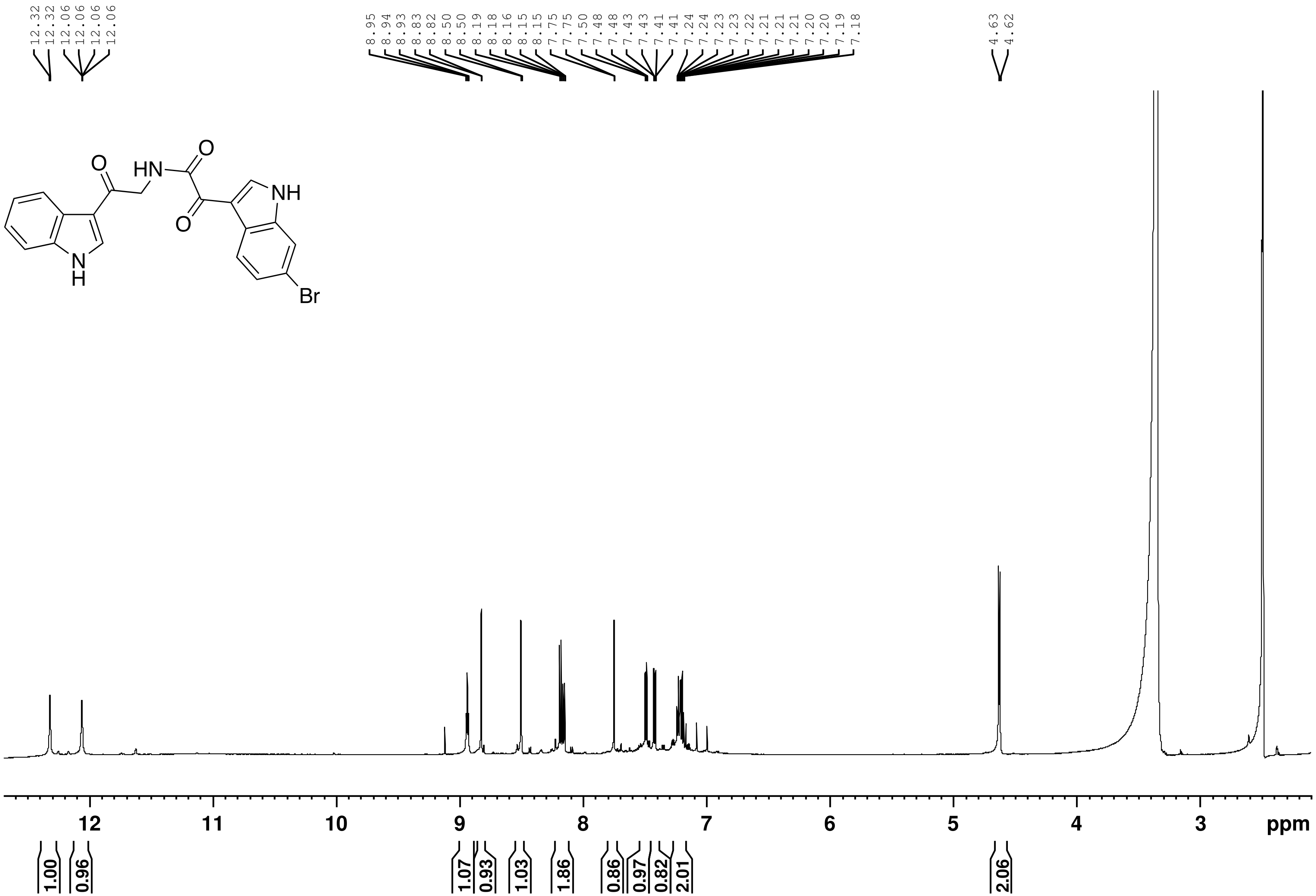

Figure S37. ^{1}H NMR spectrum (600 MHz) of lamellomorphamide C (**5**) in DMSO-d_6

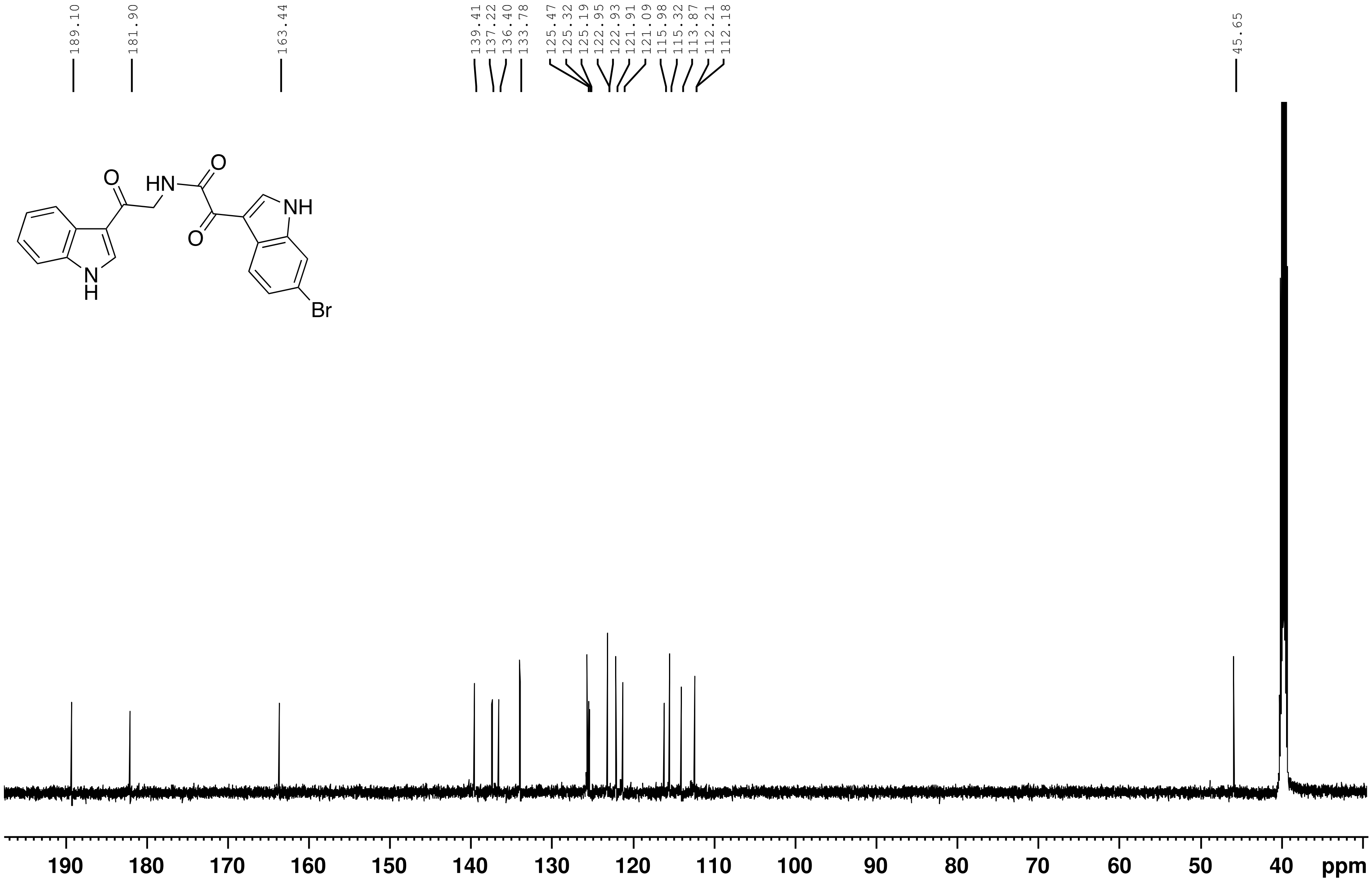

Figure S38. ^{13}C NMR spectrum (150 MHz) of lamellomorphamide C (**5**) in DMSO-d_6

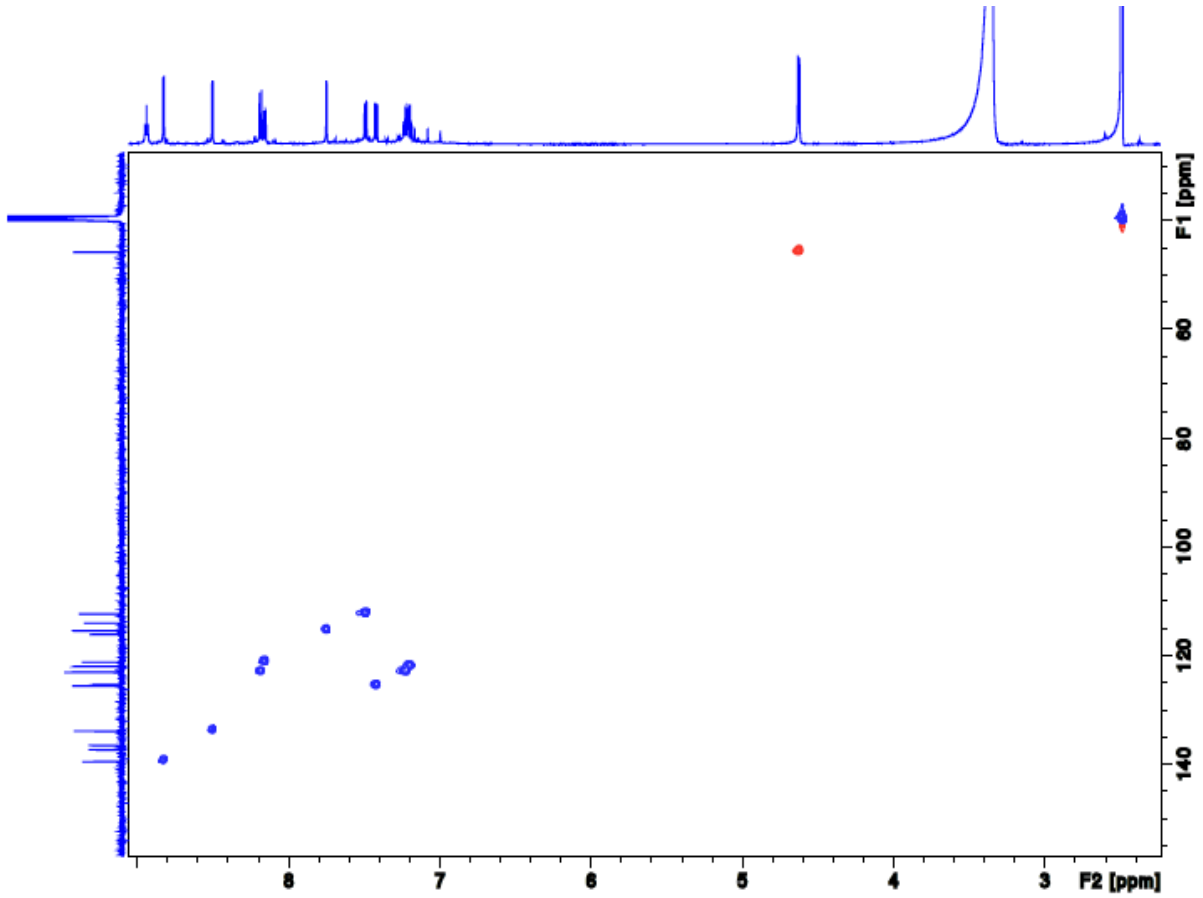

Figure S39. ^{1}H- ^{13}C HSQC spectrum (600 MHz) of lamellomorphamide C (**5**) in DMSO-d_6

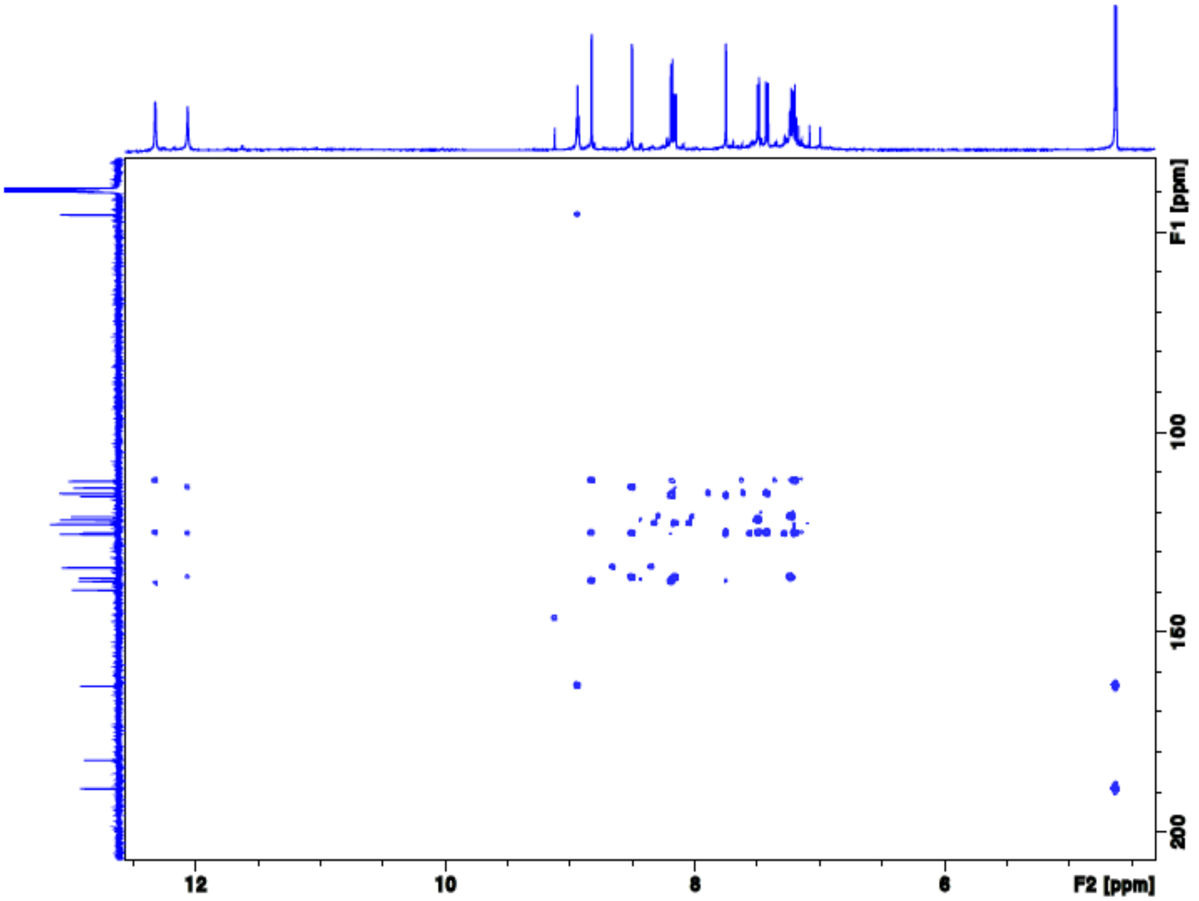

Figure S40. ^{1}H- ^{13}C HMBC spectrum (600 MHz) of lamellomorphamide C (**5**) in DMSO-d_6

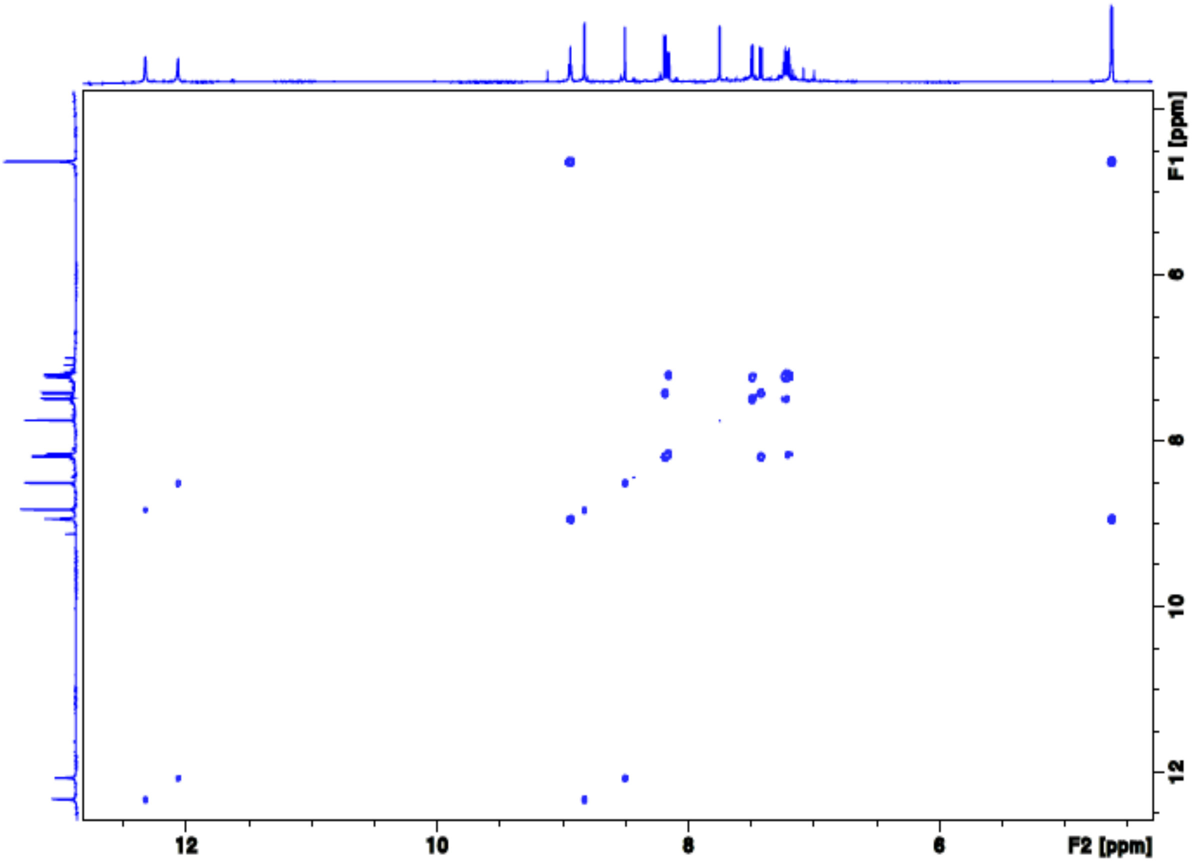

Figure S41. ^{1}H-^{1}H COSY spectrum (600 MHz) of lamellomorphamide C (**5**) in DMSO-d_6

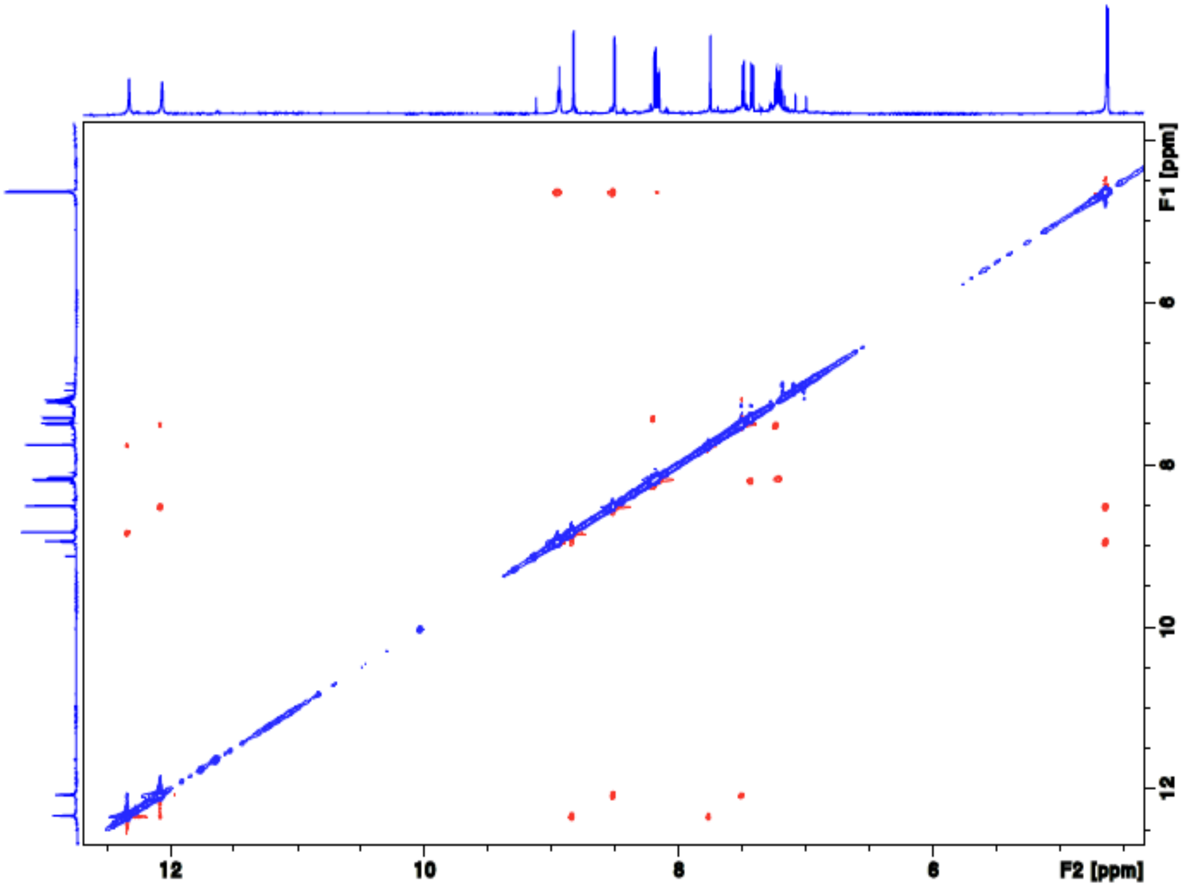

Figure S42. ^{1}H-^{1}H ROESY spectrum (600 MHz) of lamellomorphamide C (**5**) in DMSO-*d*

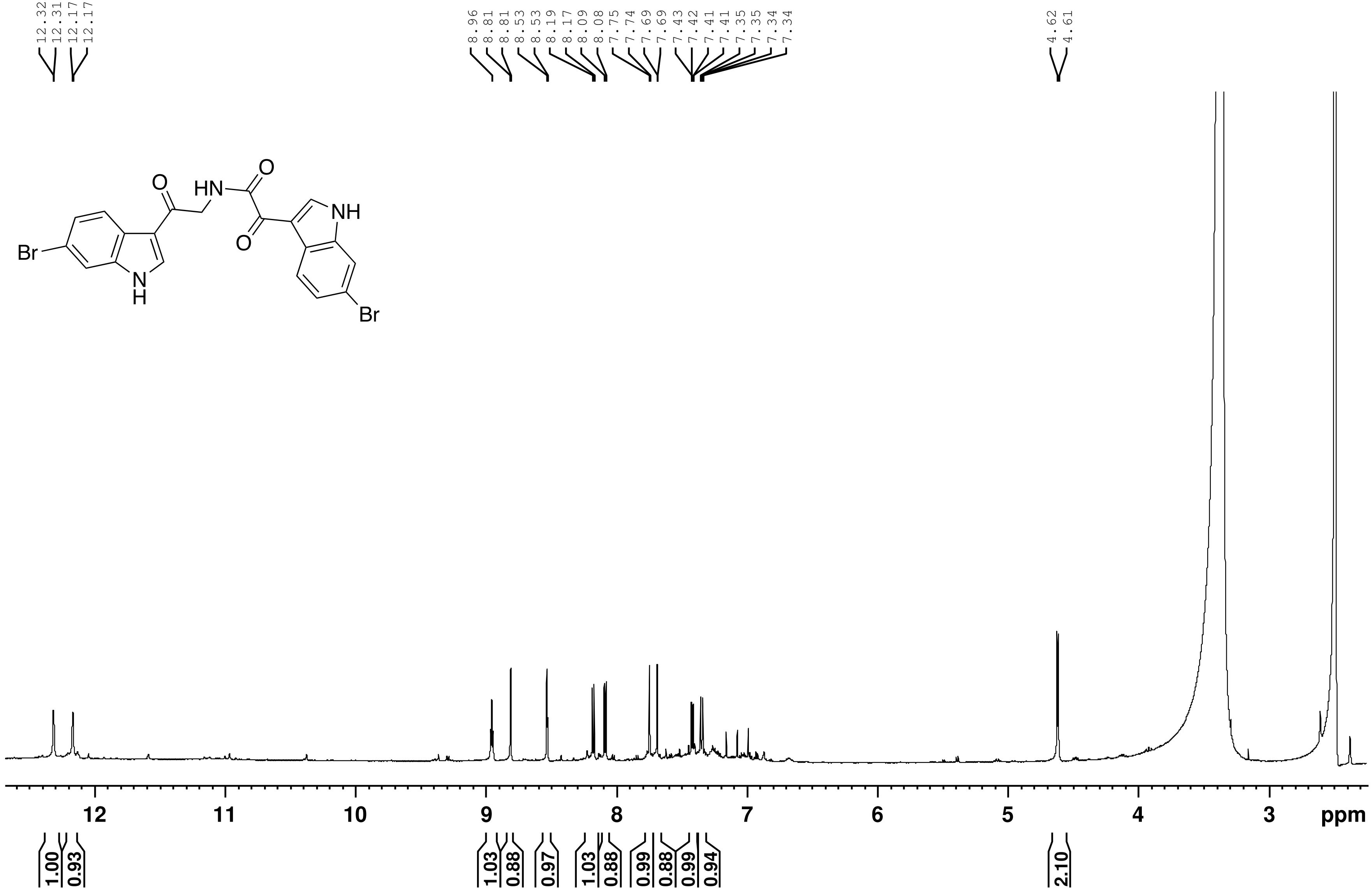

Figure S43. ^{1}H NMR spectrum (600 MHz) of lamellomorphamide D (**6**) in DMSO-d_6

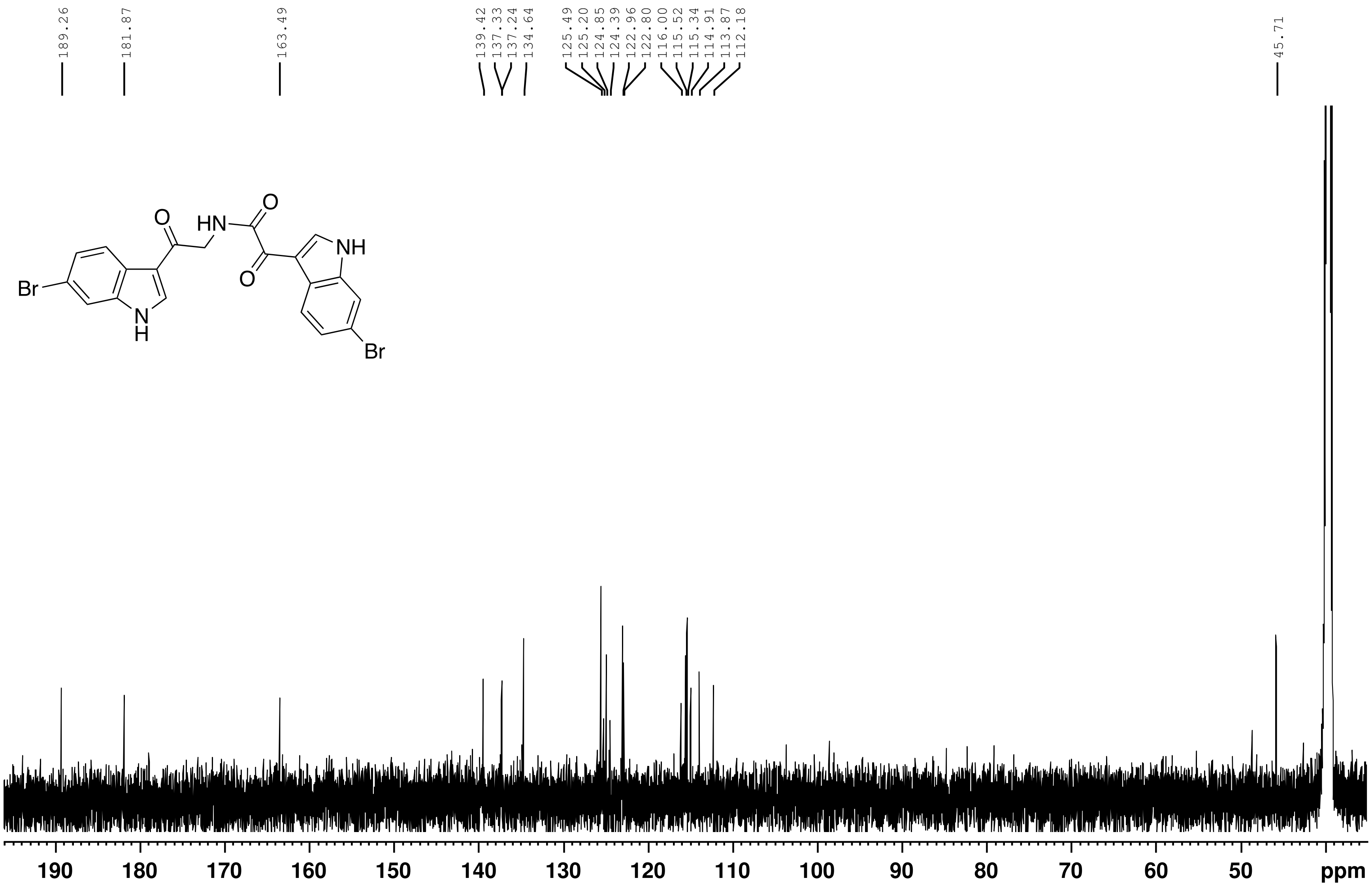

Figure S44. ^{13}C NMR spectrum (150 MHz) of lamellomorphamide D (**6**) in DMSO-d_6

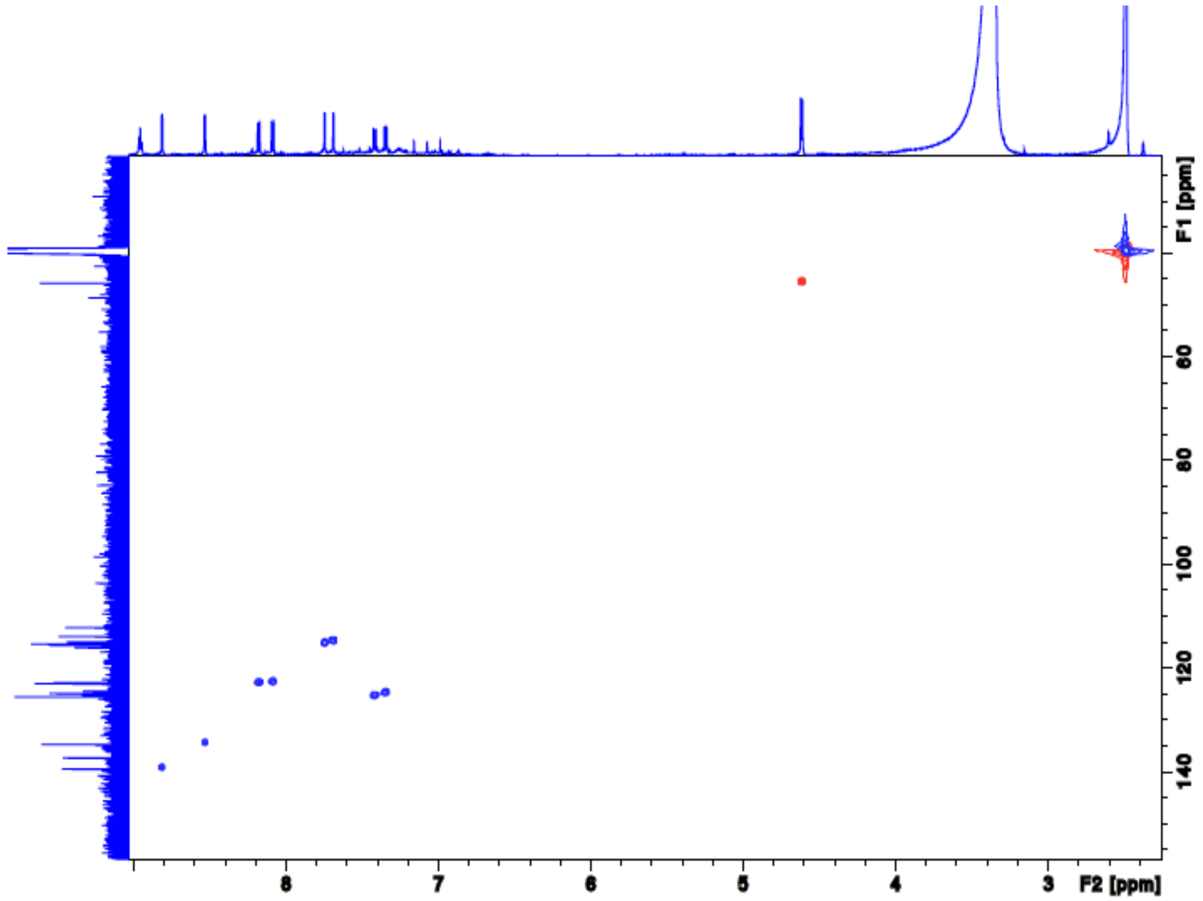

Figure S45. ^{1}H- ^{13}C HSQC spectrum (600 MHz) of lamellomorphamide D (**6**) in DMSO-d_6

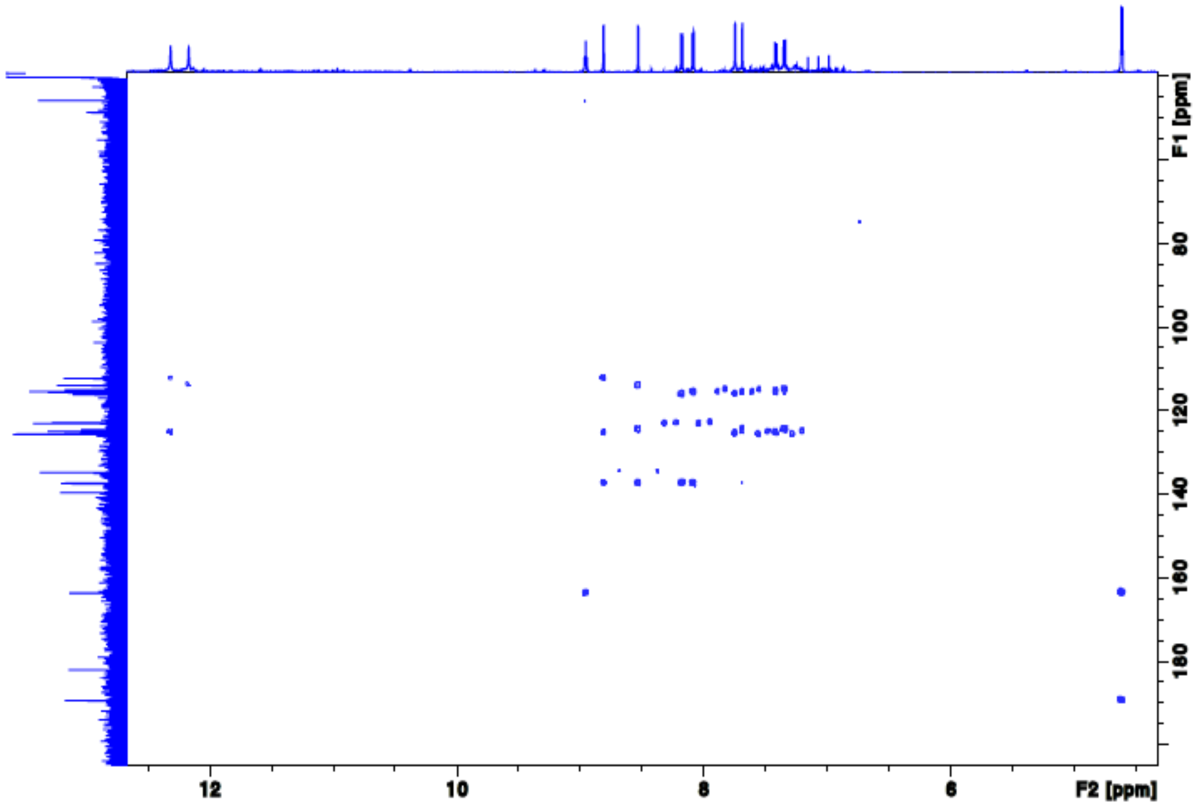

Figure S46. ^{1}H- ^{13}C HMBC spectrum (600 MHz) of lamellomorphamide D (**6**) in DMSO-d_6

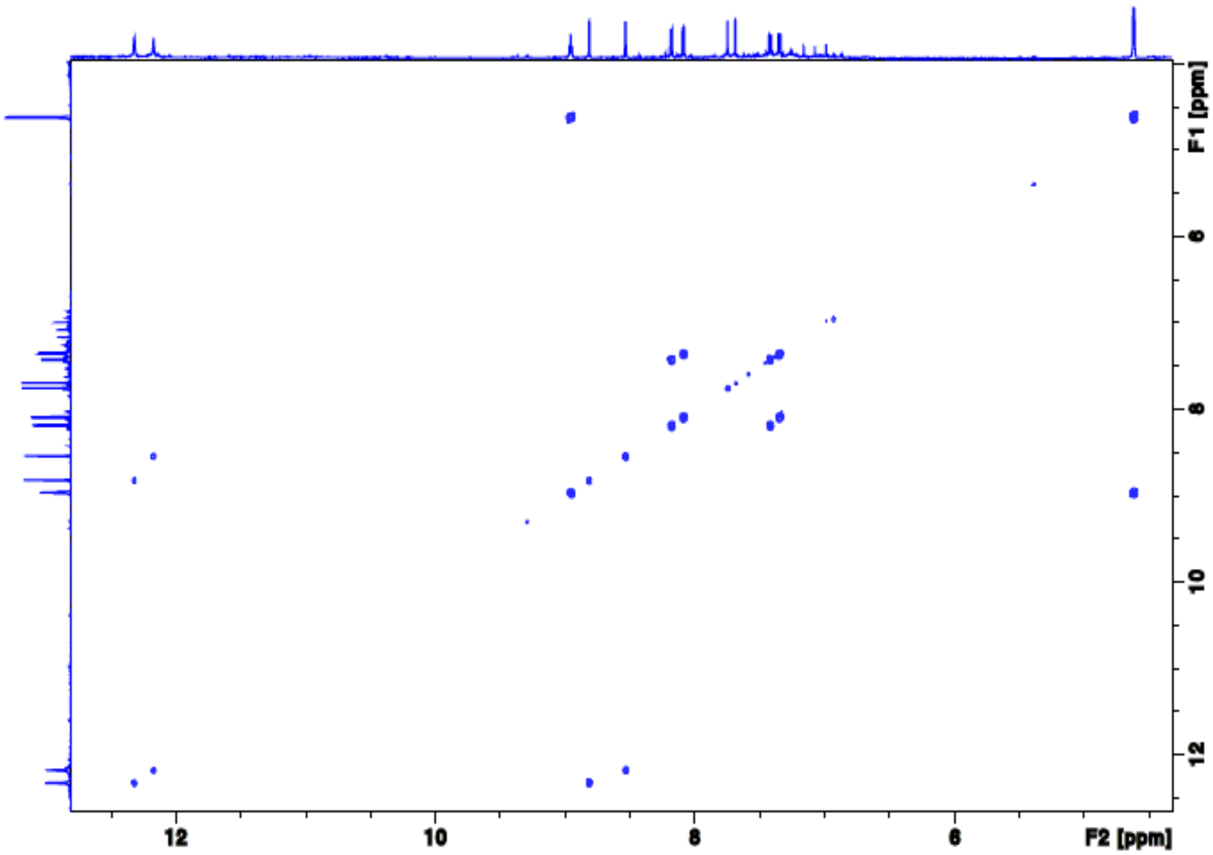

Figure S47. ^1H-^1H COSY spectrum (600 MHz) of lamellomorphamide D (**6**) in DMSO-d_6

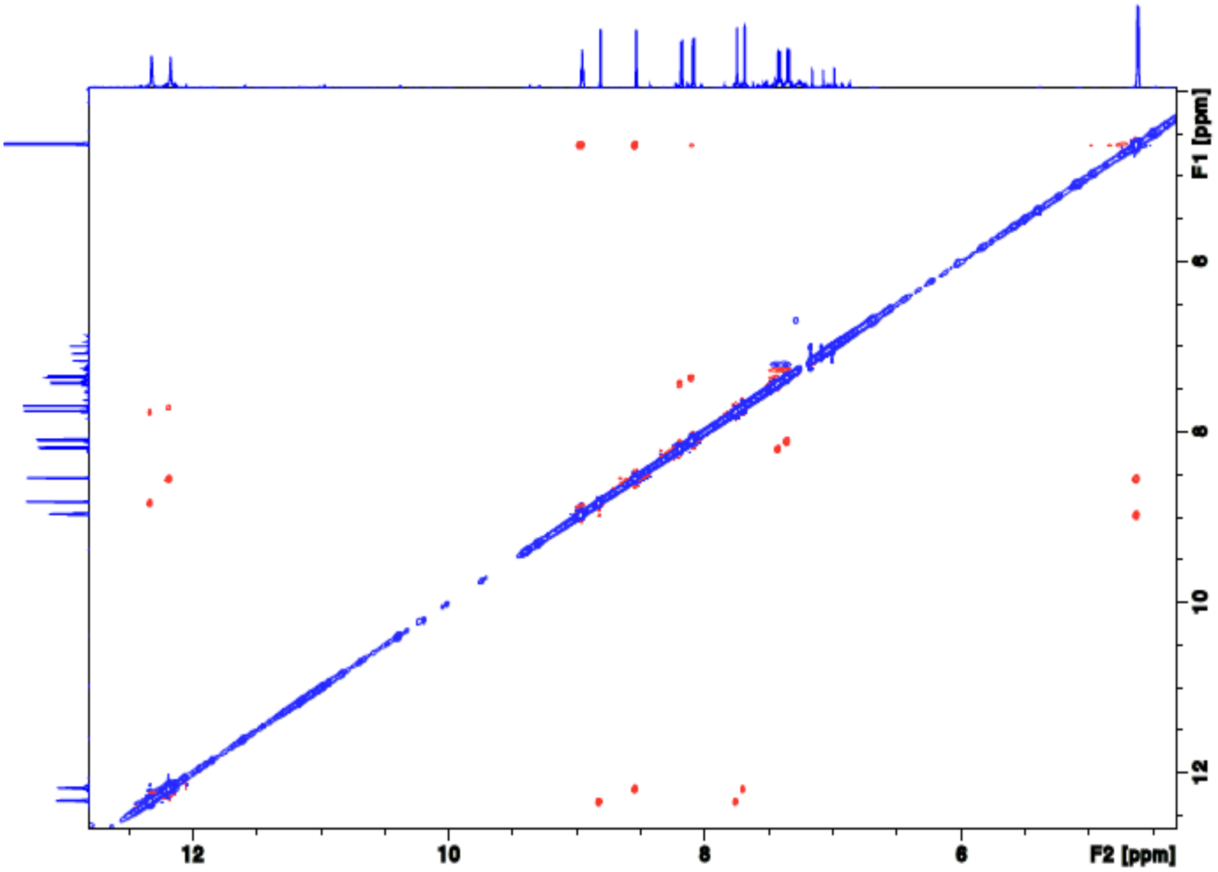

Figure S48. ^1H-^1H ROESY spectrum (600 MHz) of lamellomorphamide D (**6**) in DMSO-d_6

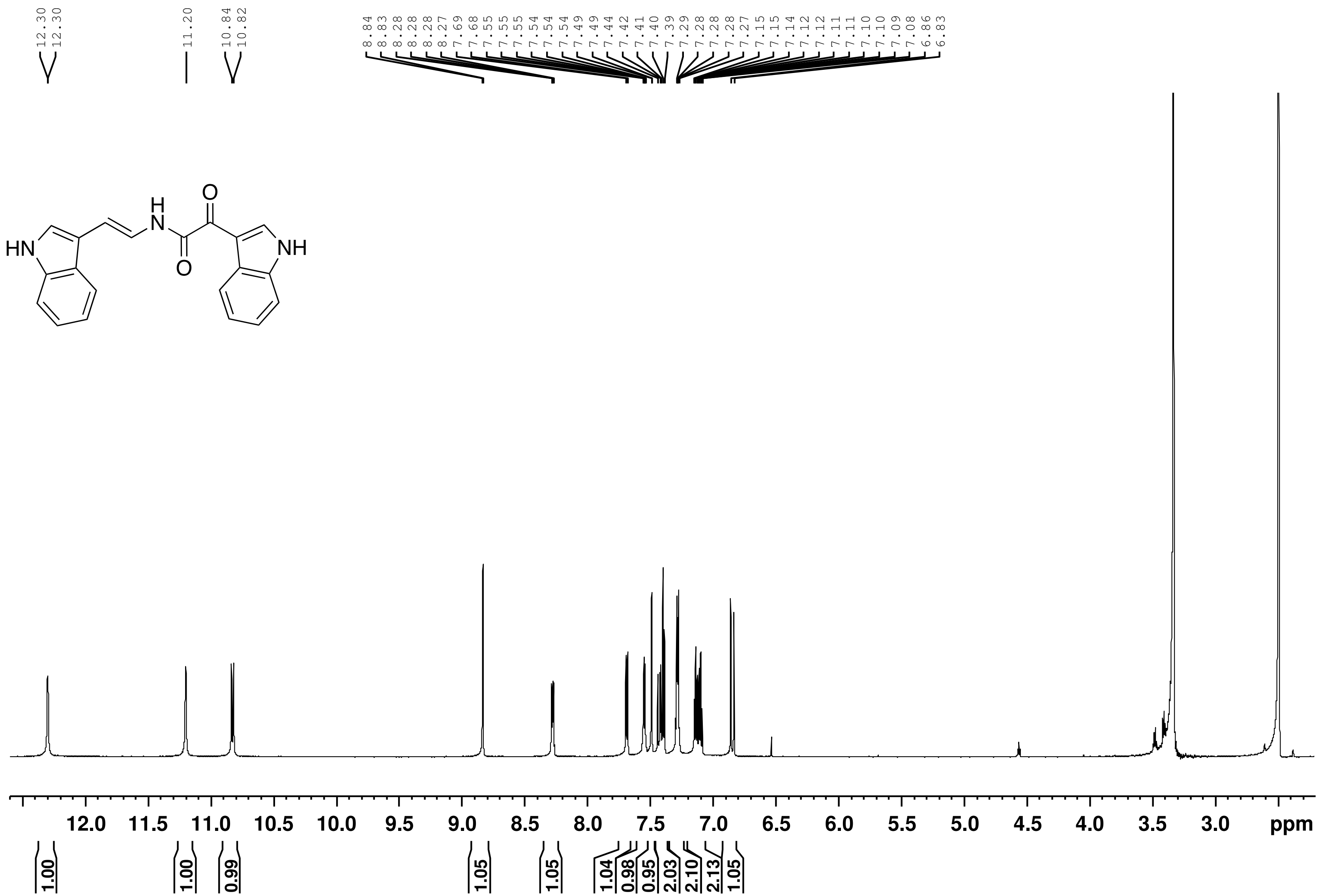

Figure S49. ^{1}H NMR spectrum (600 MHz) of coscinamide B (**7**) in DMSO-d_6

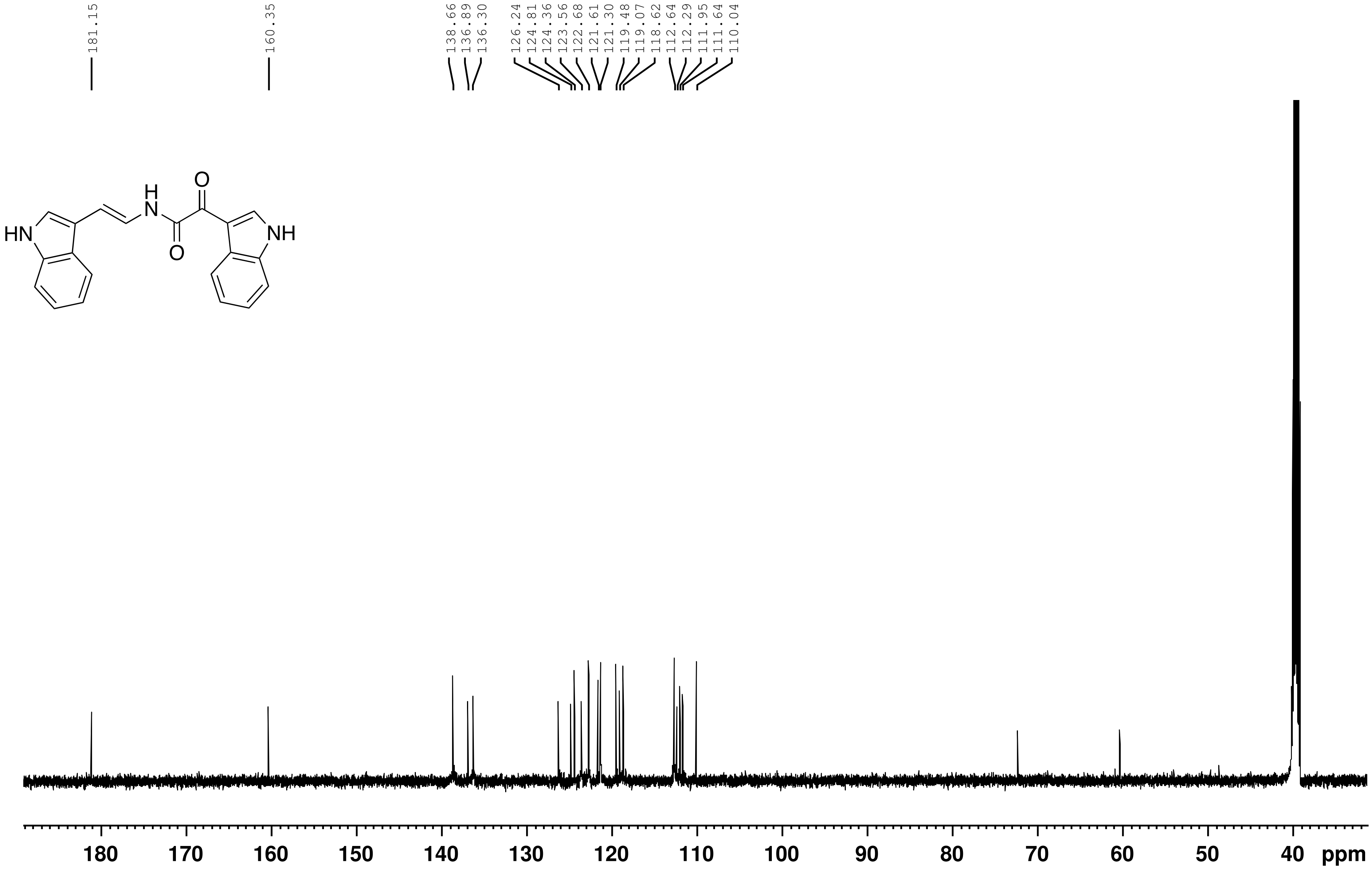

Figure S50. ^{1}H NMR spectrum (600 MHz) of coscinamide B (**7**) in DMSO-d_6

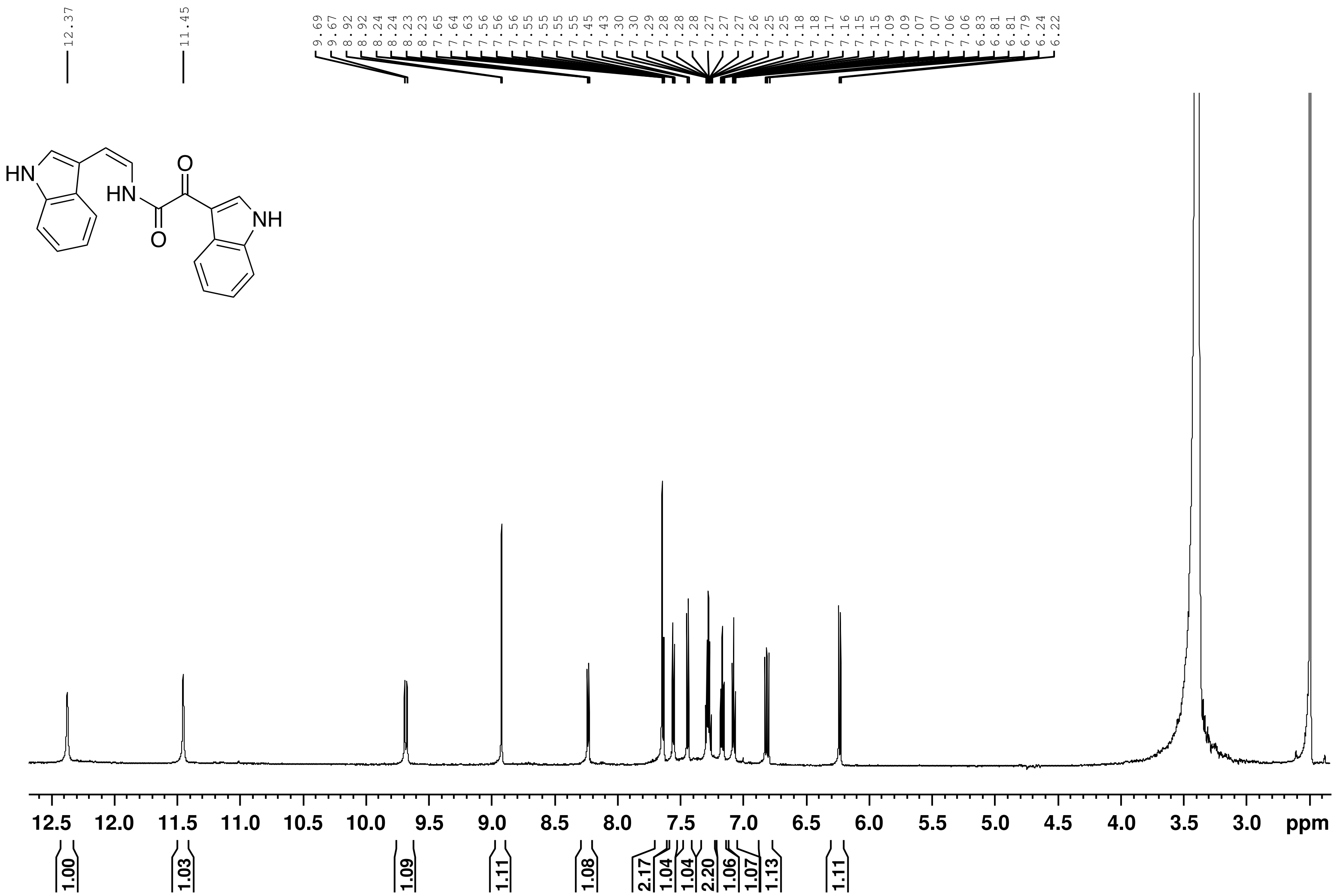

Figure S51. ^{1}H NMR spectrum (600 MHz) of (*Z*)-coscinamide B (**8**) in DMSO-d_6

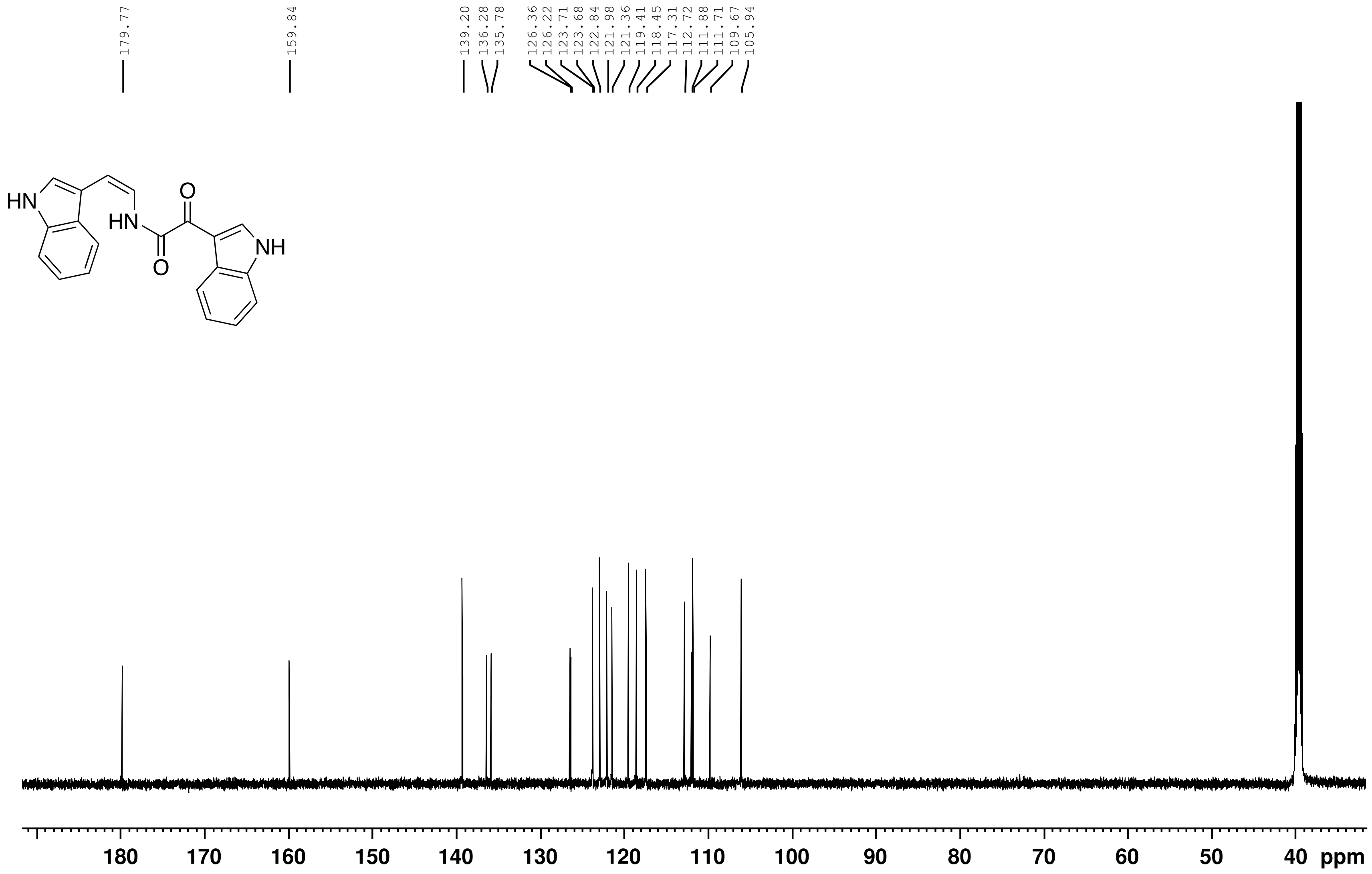

Figure S52. ^{13}C NMR spectrum (150 MHz) of (*Z*)-coscinamide B (**8**) in DMSO-d_6

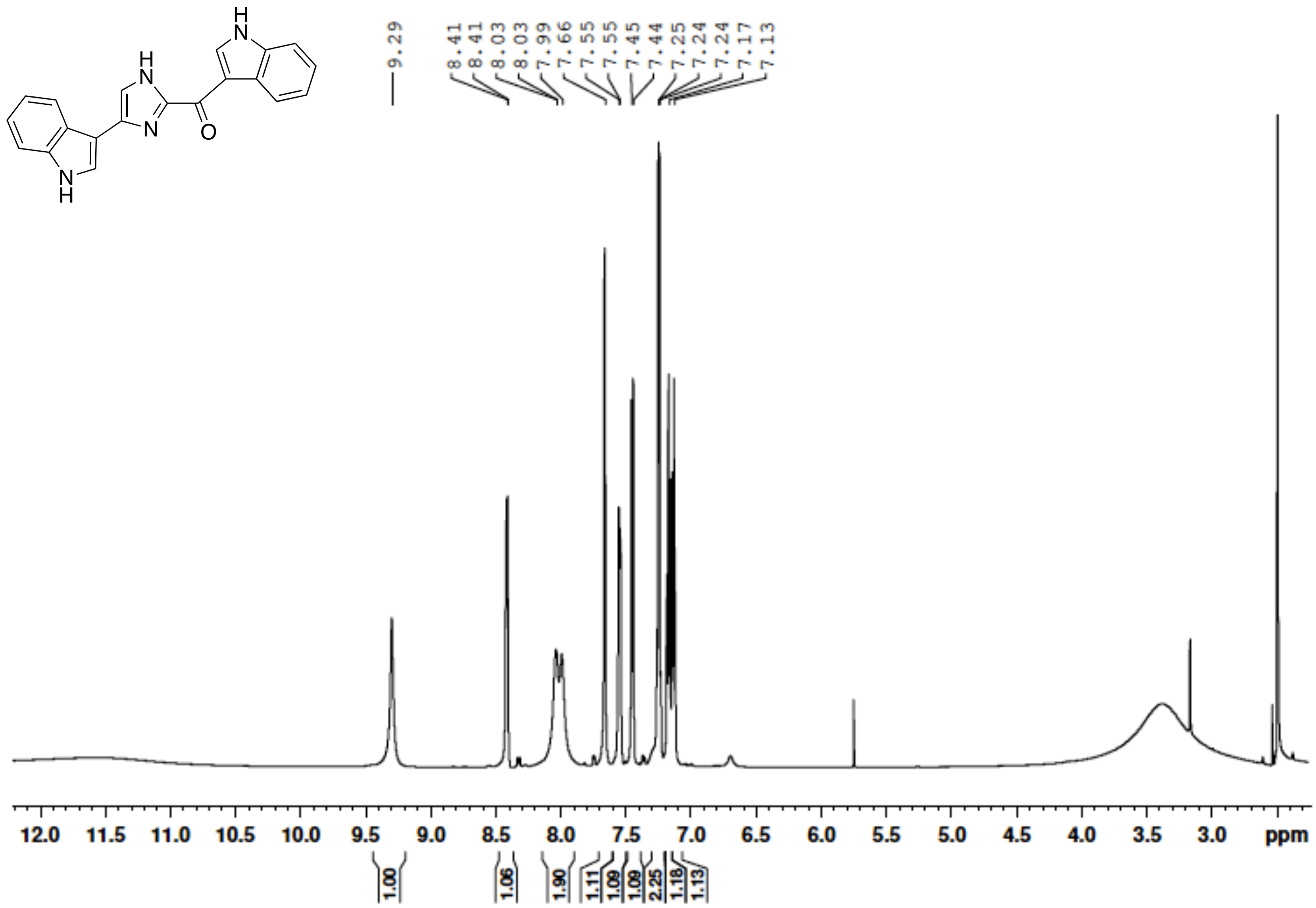

Figure S53. ^{1}H NMR spectrum (600 MHz) of deoxytopsentin (**9**) in DMSO-d_6

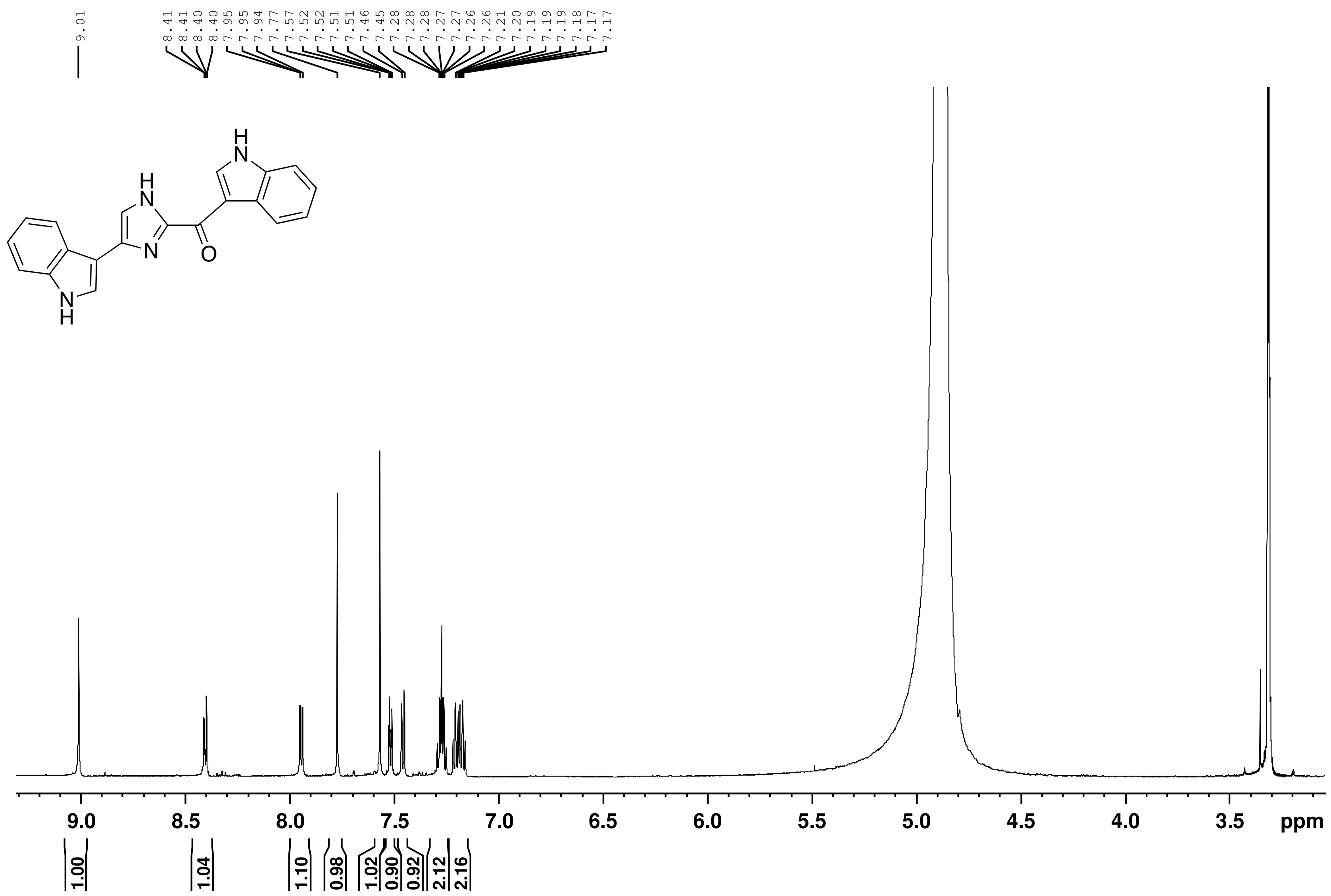

Figure S54. ^{1}H NMR spectrum (600 MHz) of deoxytopsentin (**9**) in methanol-d_4

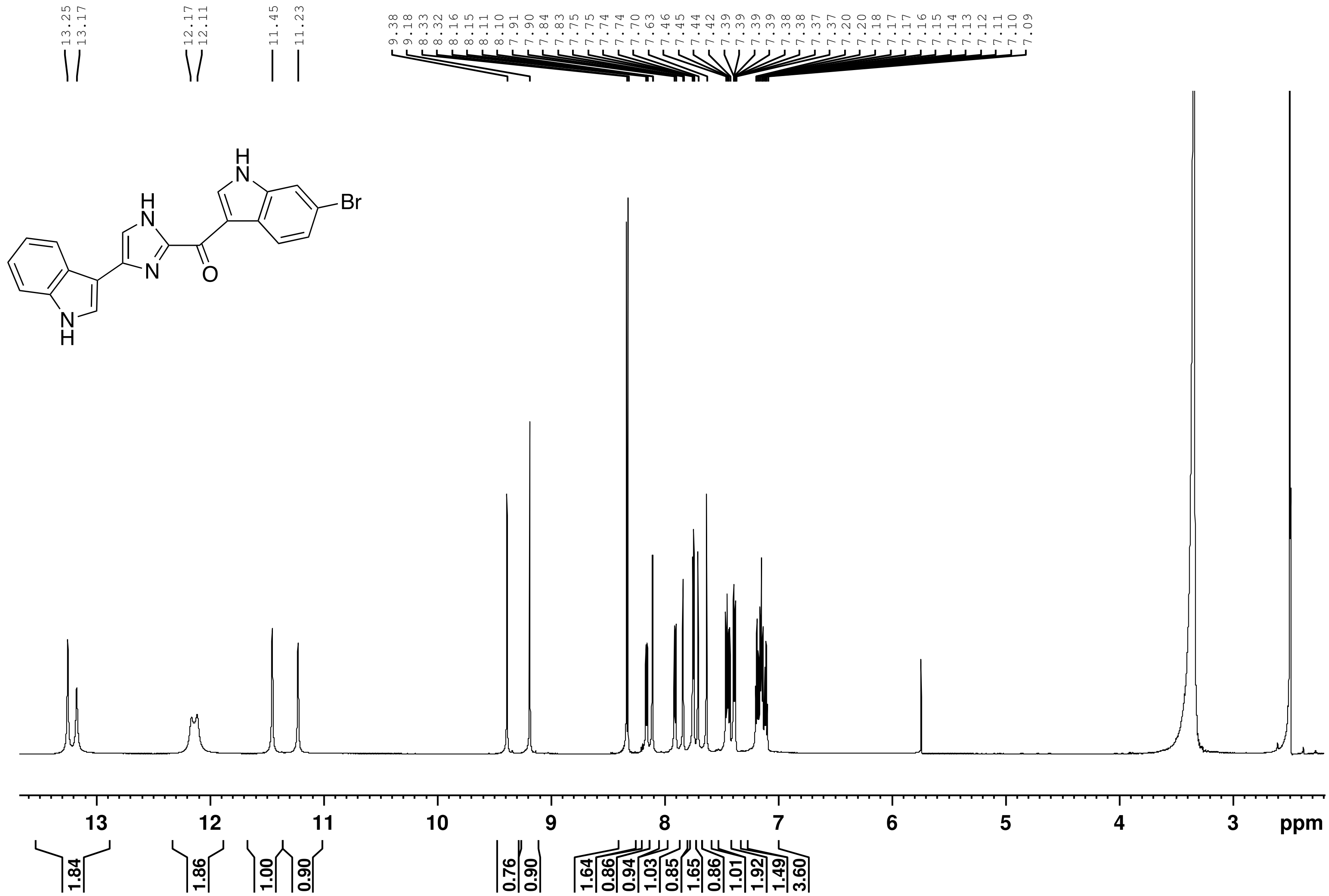

Figure S55. ^{1}H NMR spectrum (600 MHz) of isobromodeoxytopsentin (**10**) in DMSO-d_6

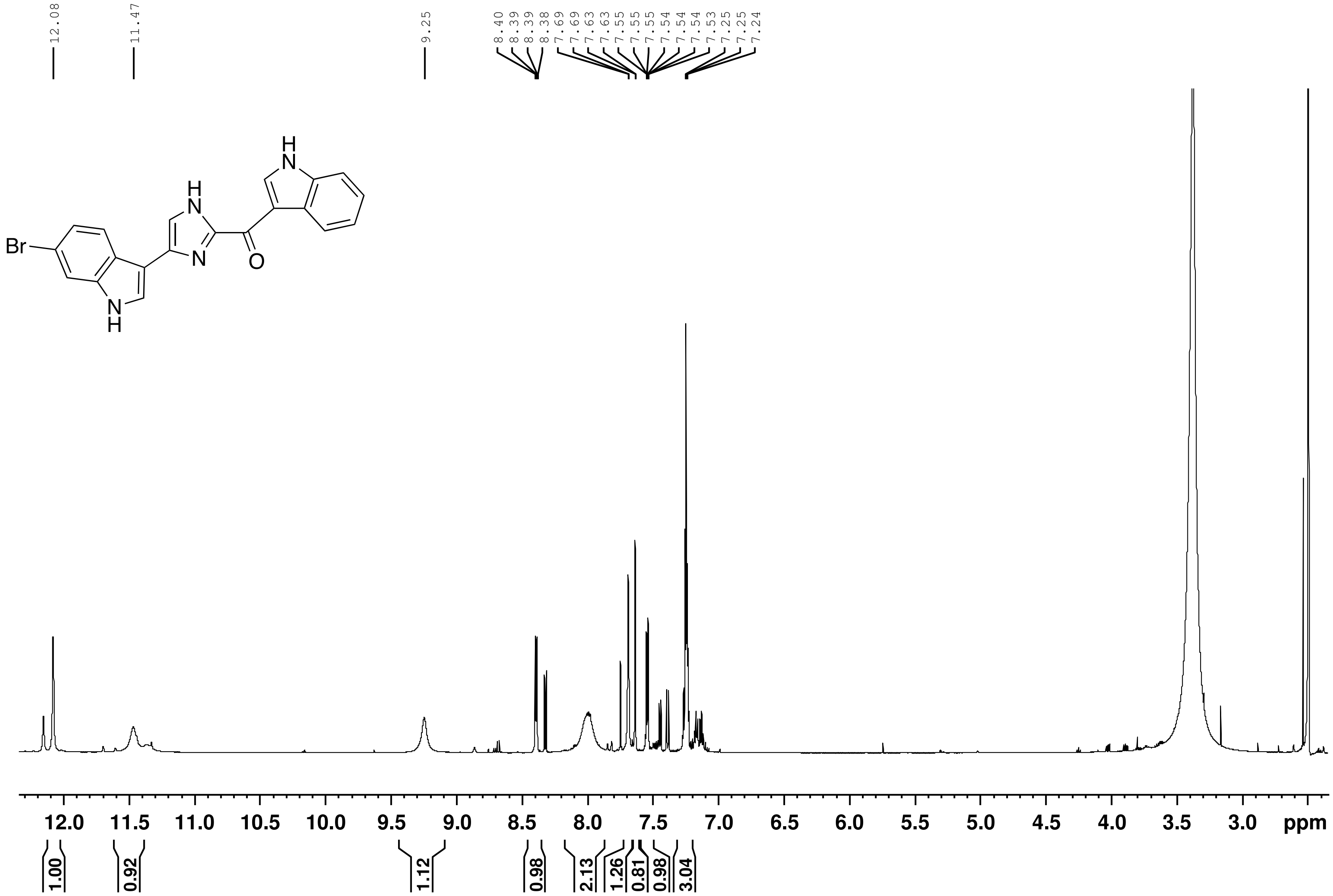

Figure S56. ^{1}H NMR spectrum (600 MHz) of bromodeoxytopsentin (**11**) in DMSO-d_6

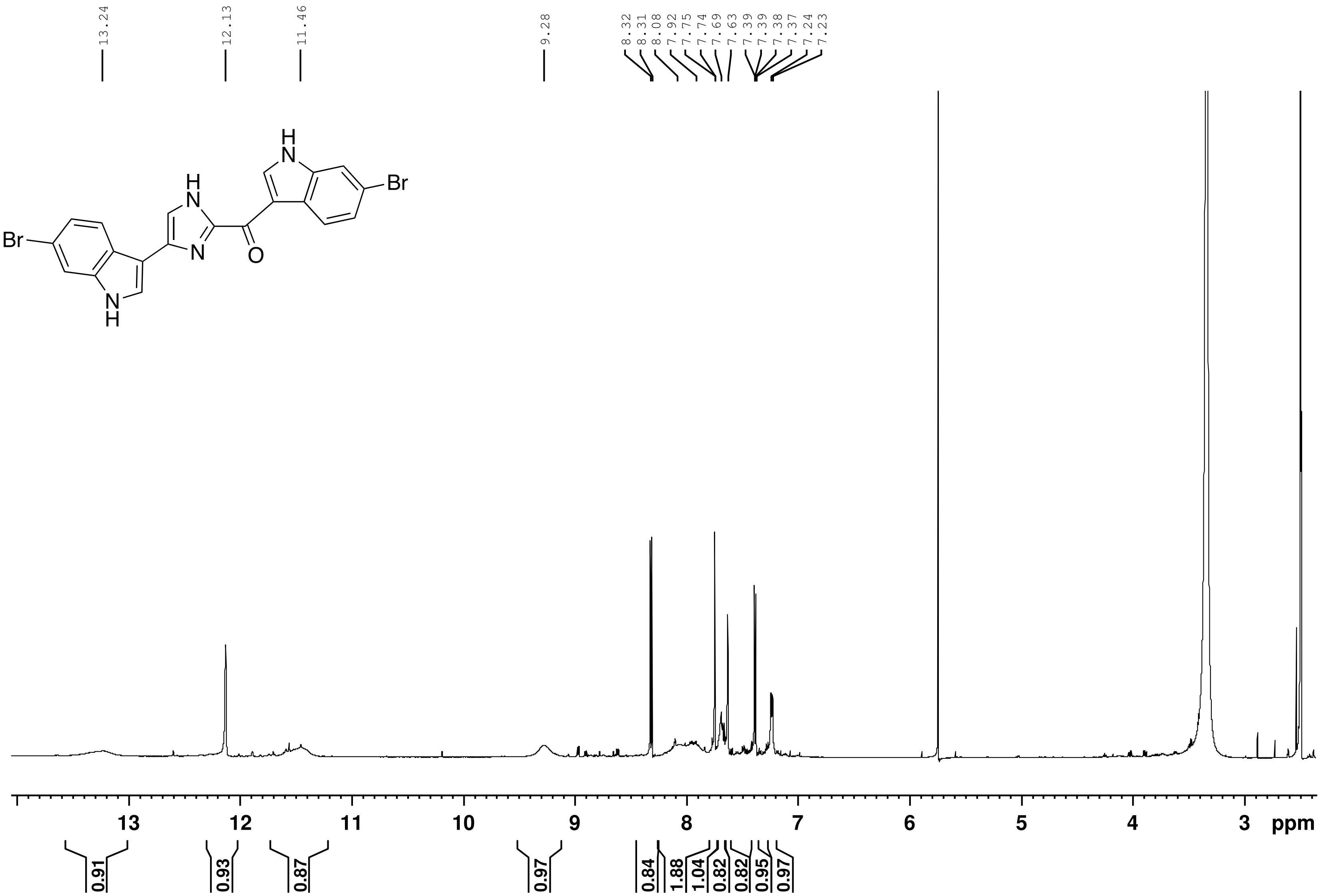

Figure S57. ^{1}H NMR spectrum (600 MHz) of dibromodeoxytopsentin (**12**) in DMSO-d_6

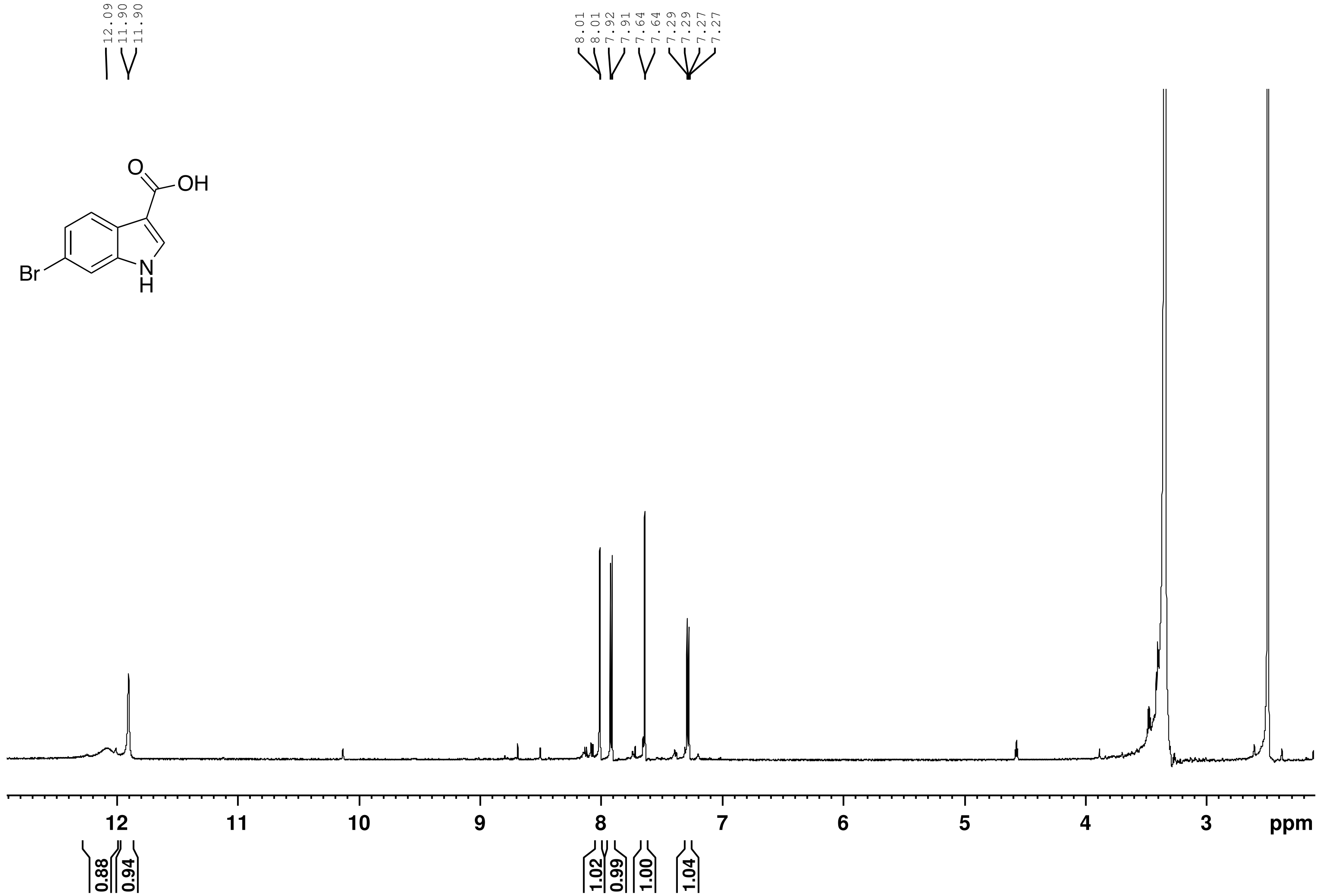

Figure S58. ^{1}H NMR spectrum (600 MHz) of 6-bromoindole-3-carboxylic acid (**13**) in DMSO-d_6

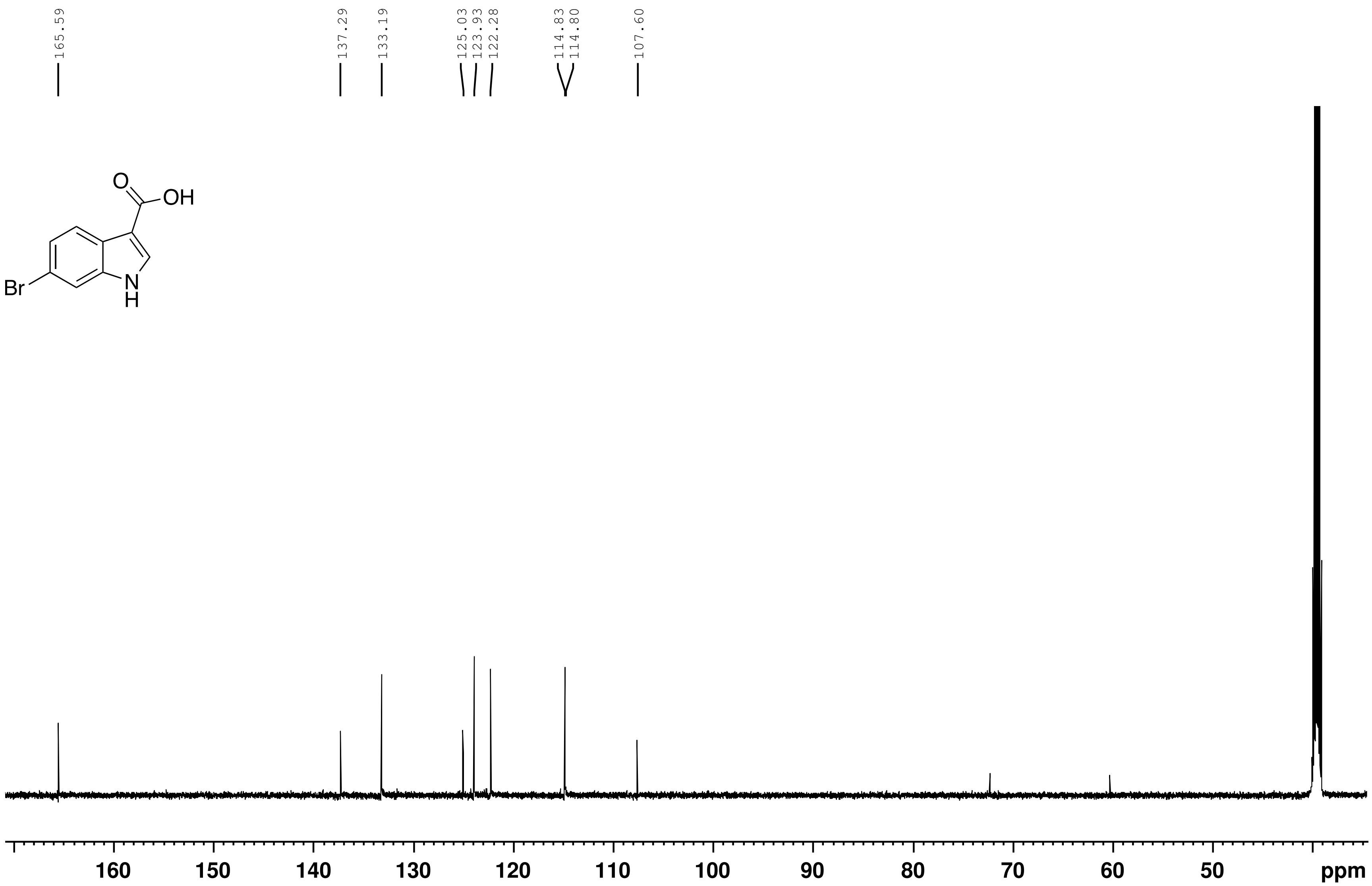

Figure S59. ^{13}C NMR spectrum (150 MHz) of 6-bromoindole-3-carboxylic acid (**13**) in DMSO-d_6

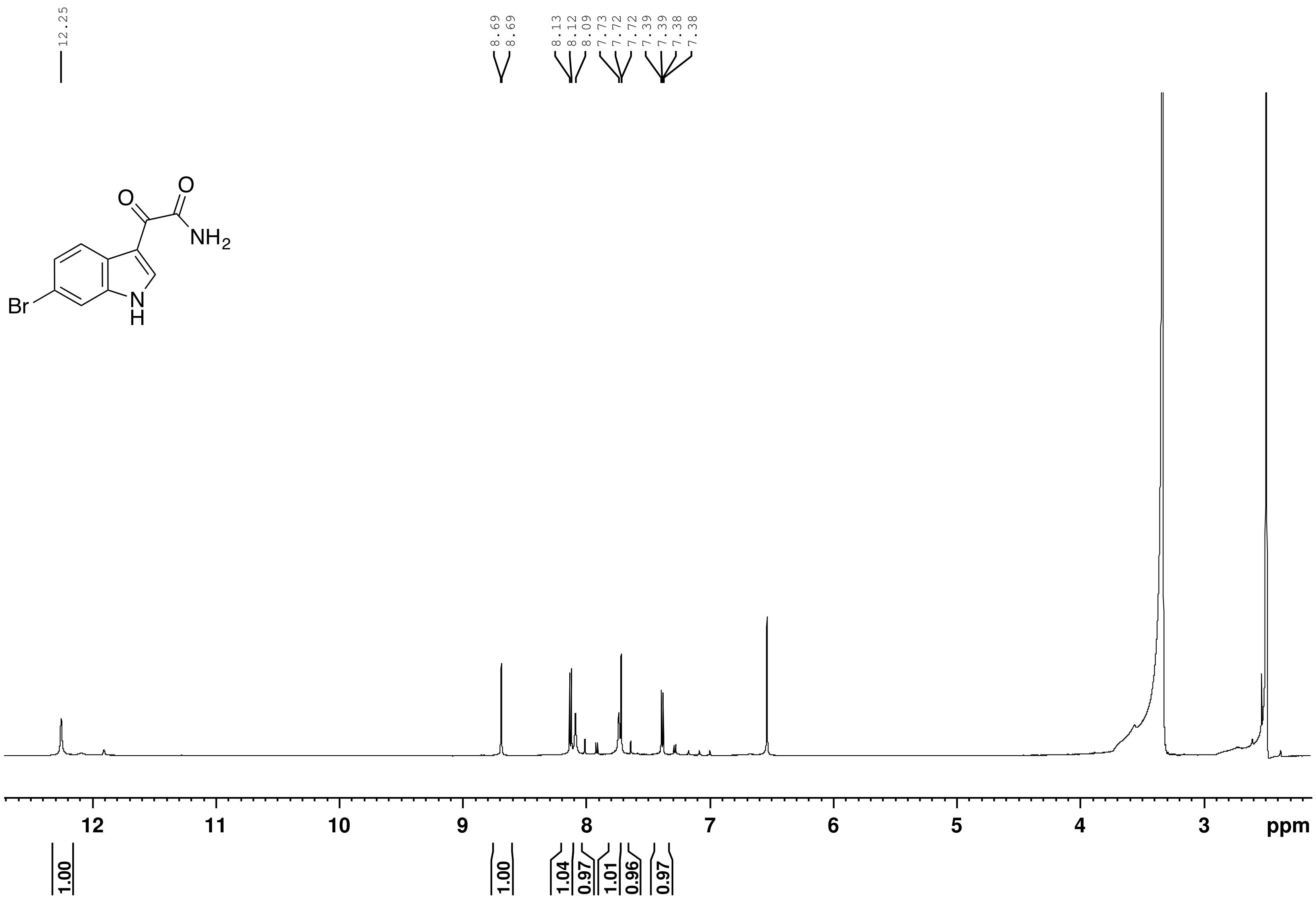

Figure S60. ^{1}H NMR spectrum (600 MHz) of (6-bromo-1*H*-indol-3-yl) oxoacetamide (**14**) in DMSO-d_6

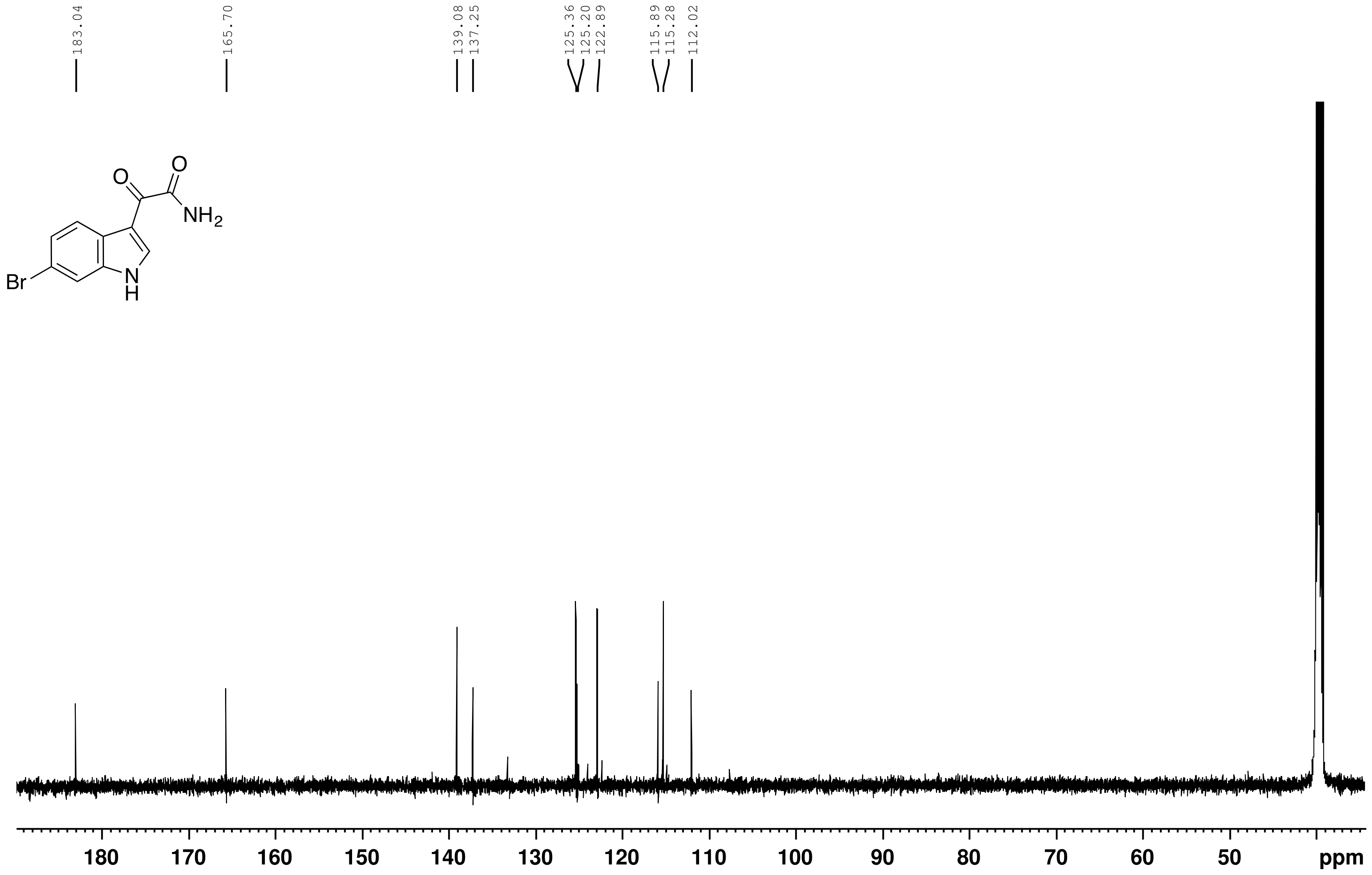

Figure S61. ^{13}C NMR spectrum (150 MHz) of (6-bromo-1*H*-indol-3-yl) oxoacetamide (**14**) in DMSO-d_6

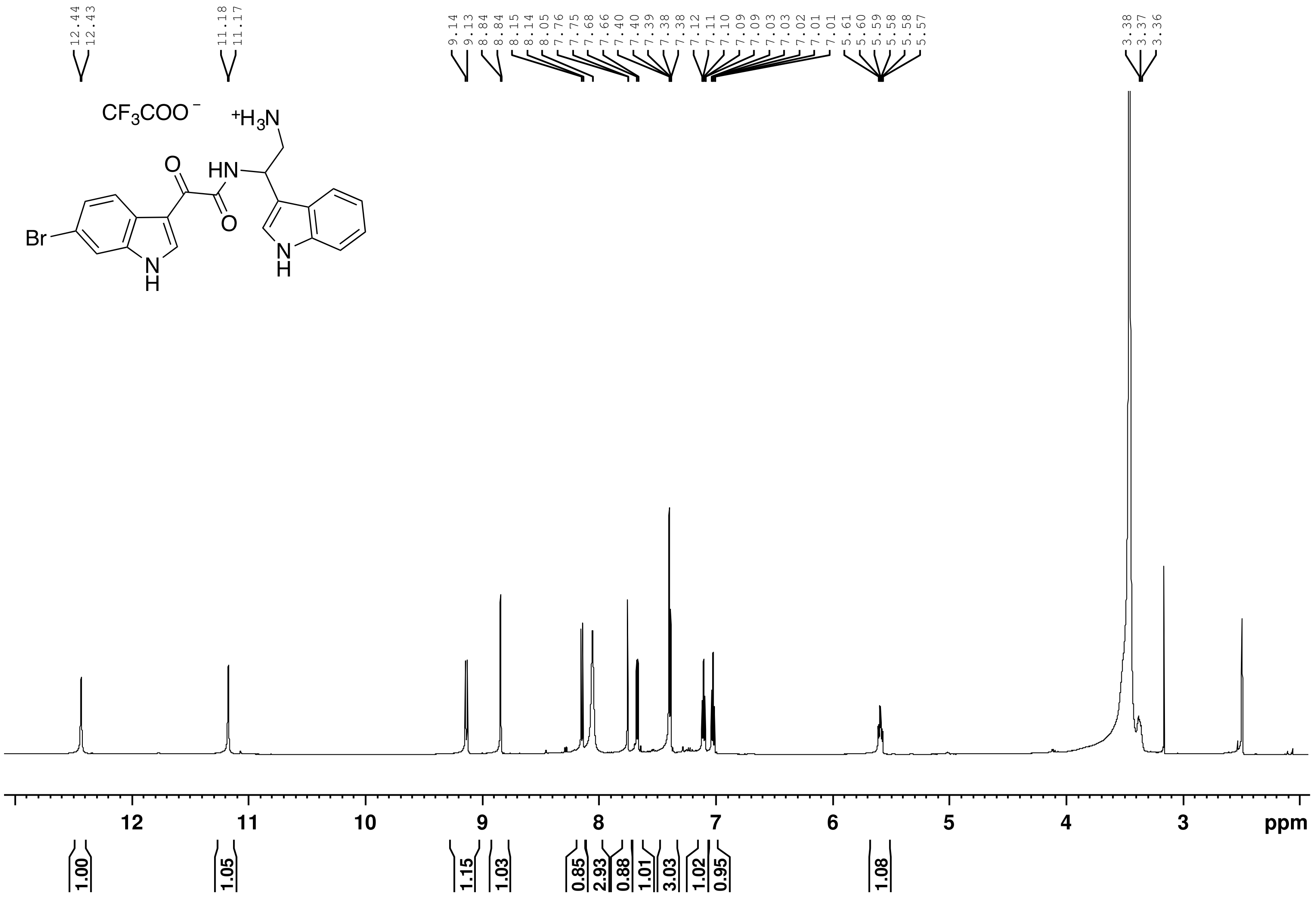

Figure S62. ^{1}H NMR spectrum (600 MHz) of 3,4-seco-6″-debromohamacanthin A (**15**) in DMSO-d_6

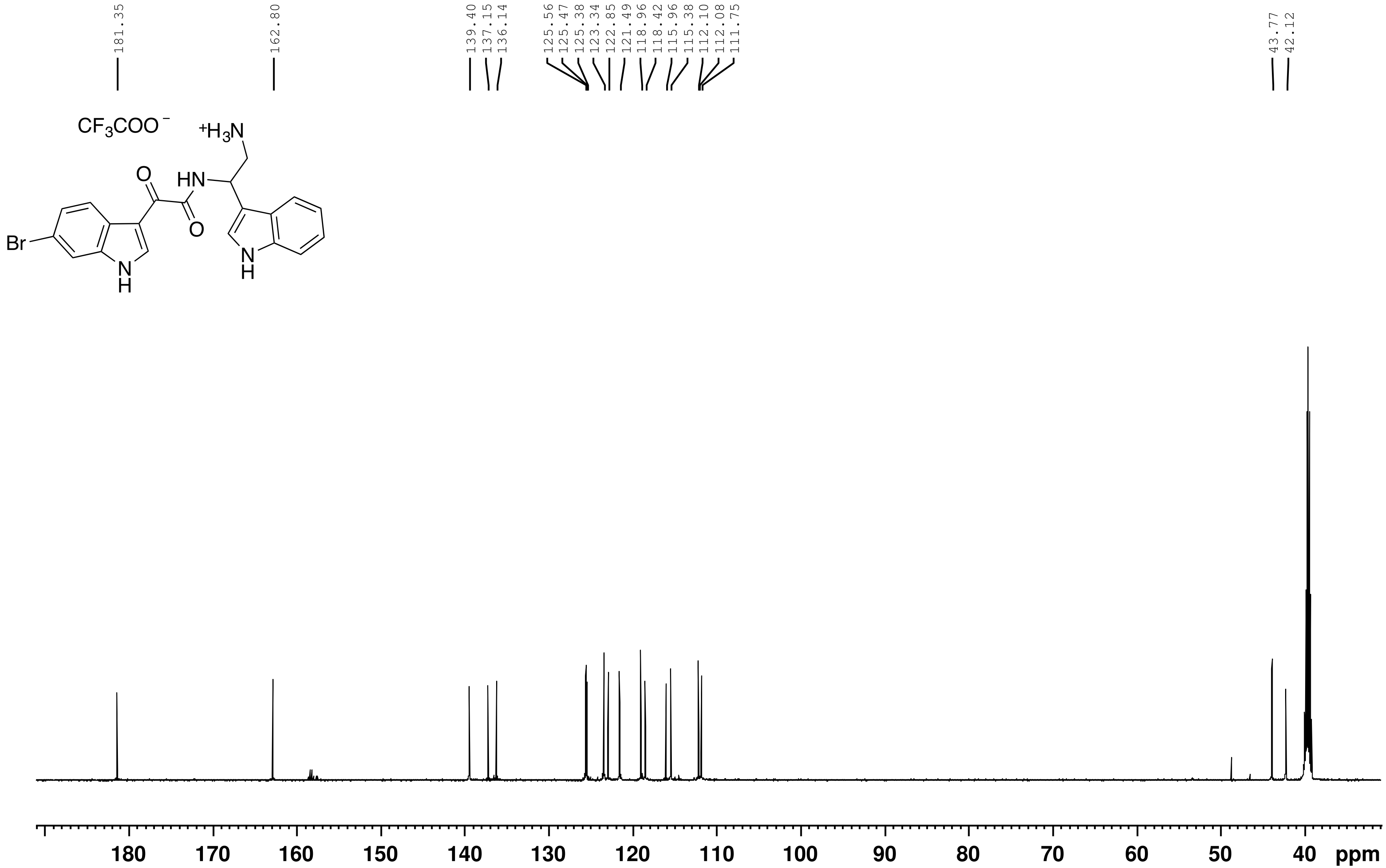

Figure S63. ^{13}C NMR spectrum (150 MHz) of 3,4-seco-6″-debromohamacanthin A (**15**) in DMSO-d_6

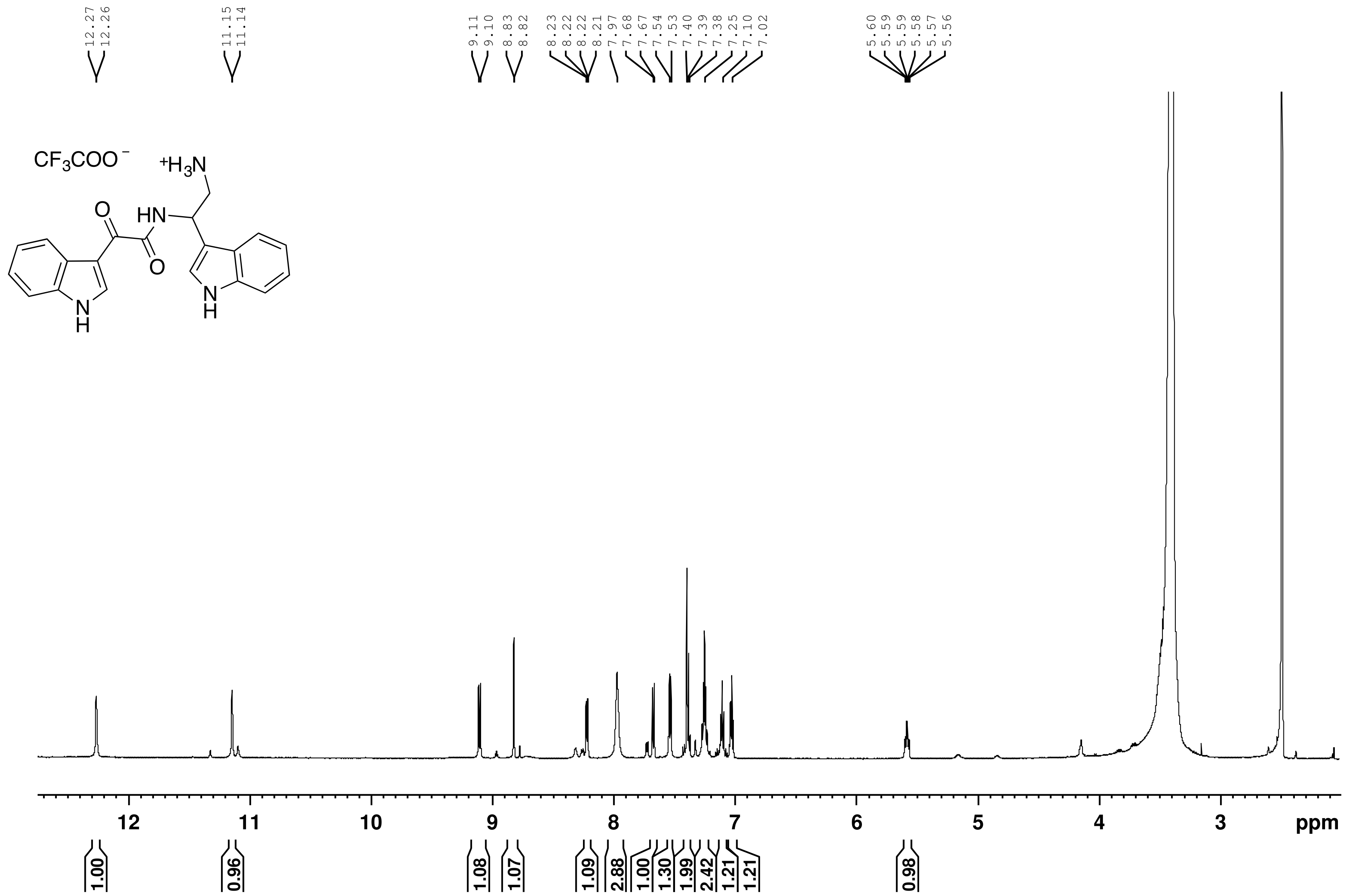

Figure S64. ^{1}H NMR spectrum (600 MHz) of 3,4-seco-6′,6″-didebromohamacanthin A (**16**) in DMSO-d_6

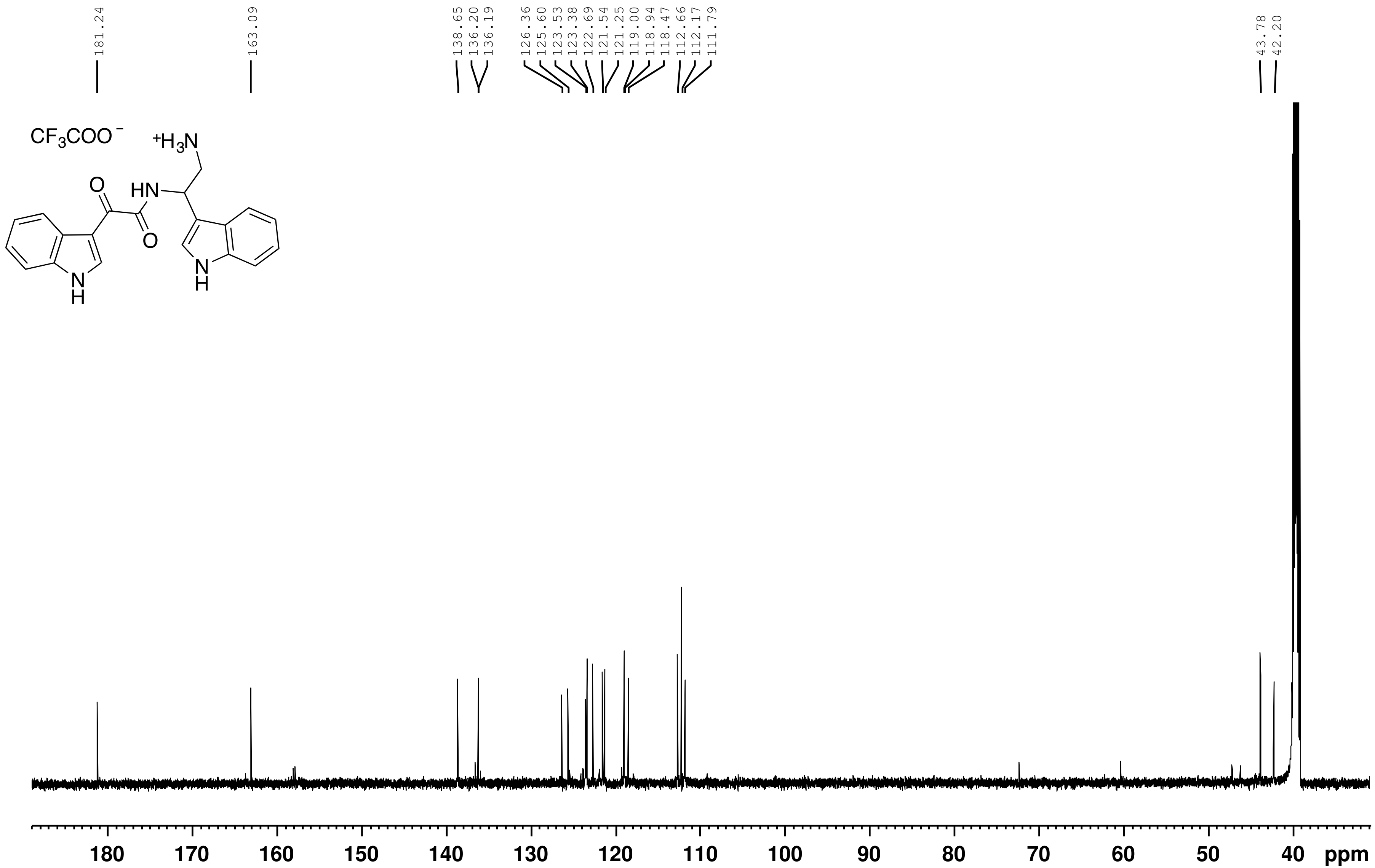

Figure S65. ^{13}C NMR spectrum (150 MHz) of 3,4-seco-6′,6″-didebromohamacanthin A (**16**) in DMSO-d_6

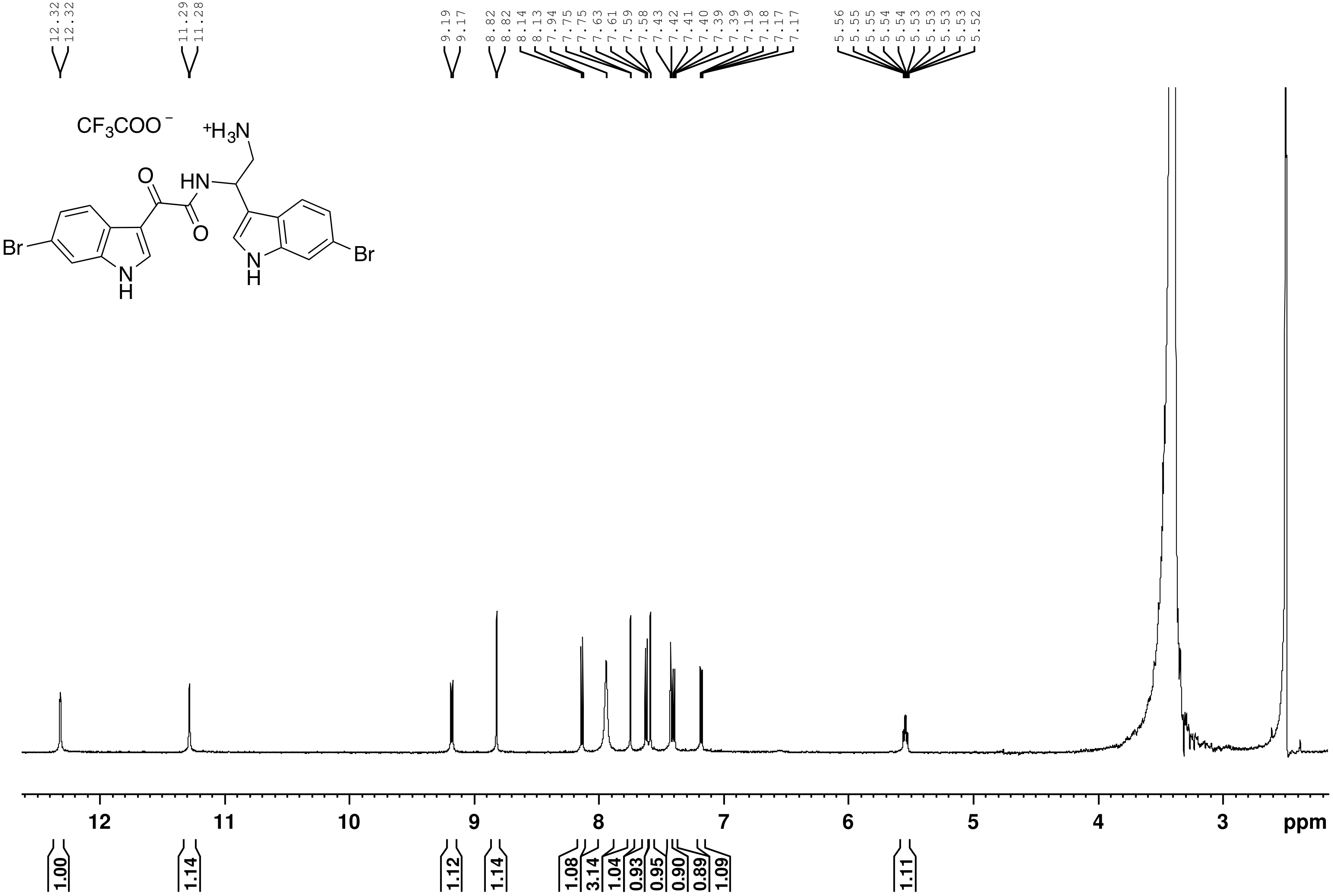

Figure S66. ^{1}H NMR spectrum (600 MHz) of 3,4-seco-hamacanthin A (**17**) in DMSO-d_6

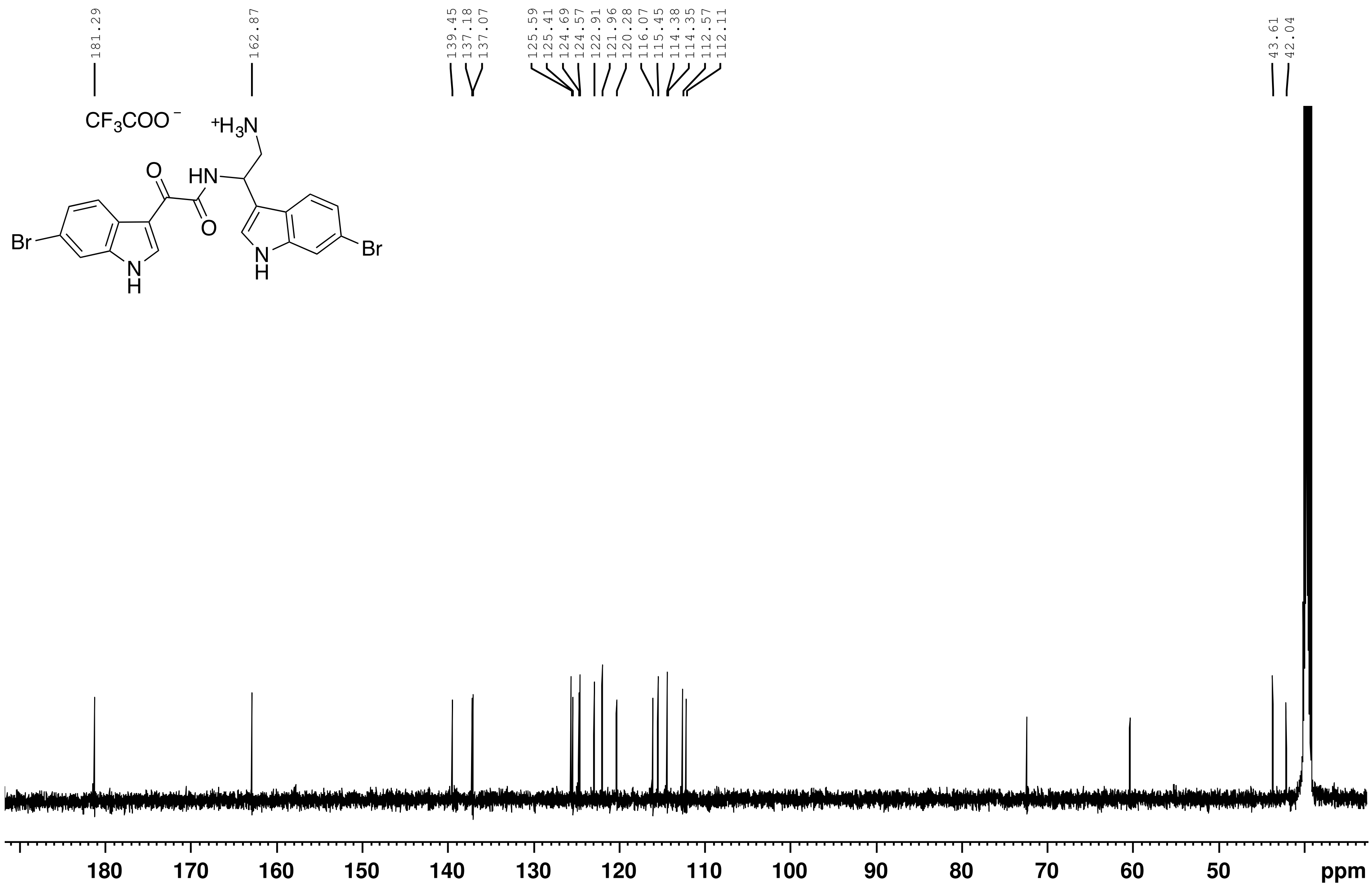

Figure S67. ^{13}C NMR spectrum (150 MHz) of 3,4-seco-hamacanthin A (**17**) in DMSO-d_6

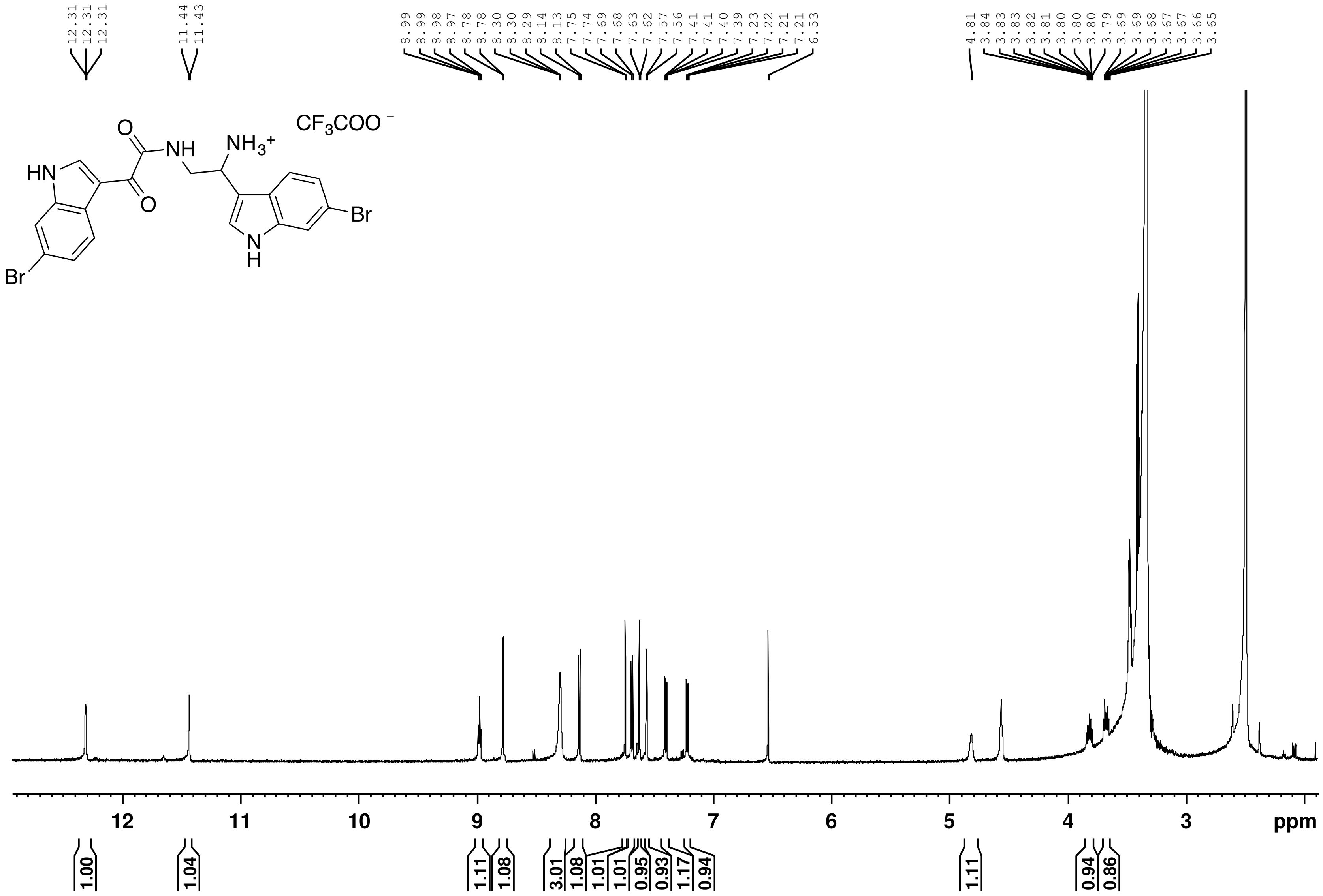

Figure S68. ^{1}H NMR spectrum (600 MHz) of 3,4-seco-hamacanthin B (**18**) in DMSO-d_6

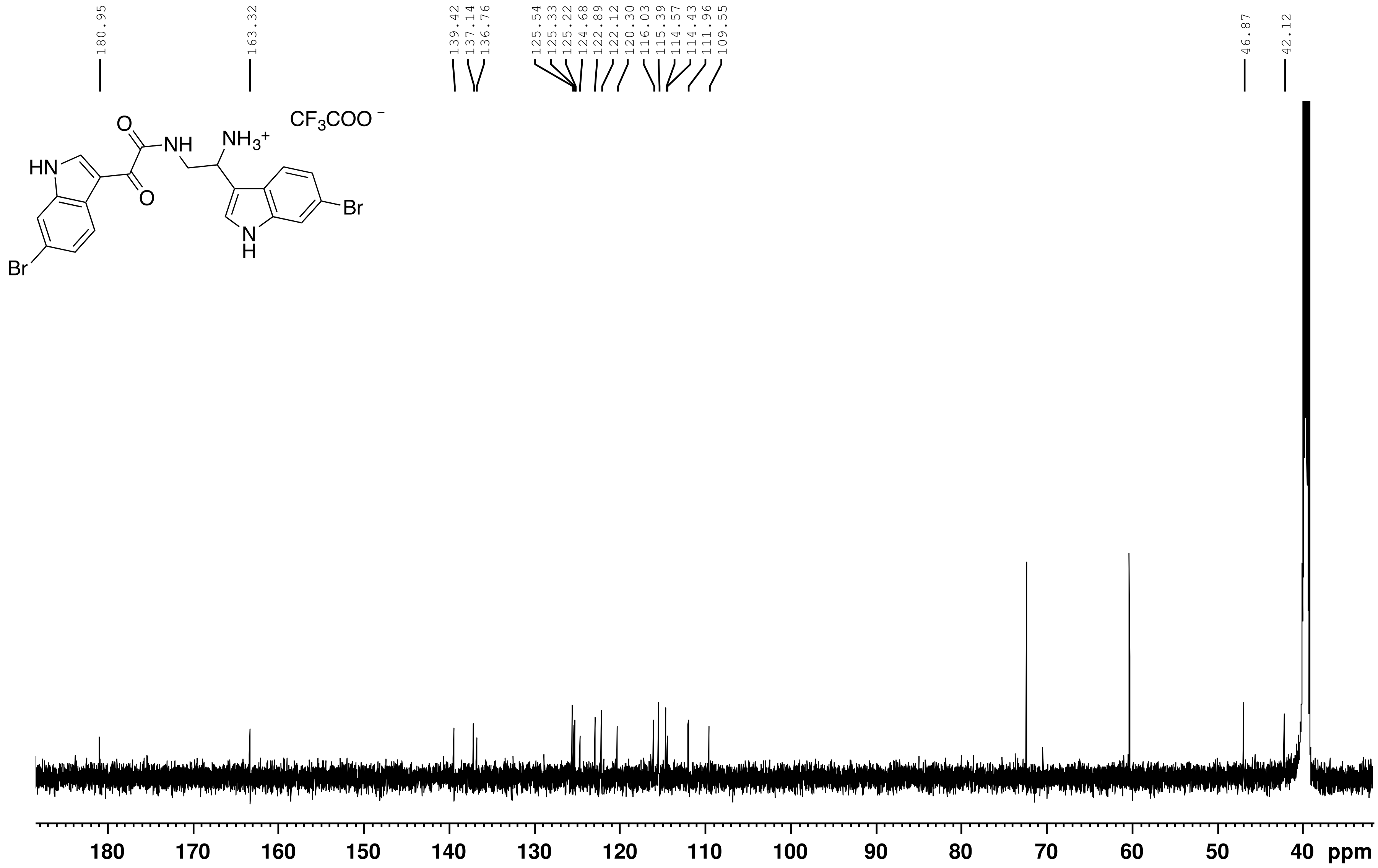

Figure S69. ^{13}C NMR spectrum (150 MHz) of 3,4-seco-hamacanthin B (**18**) in DMSO-d_6

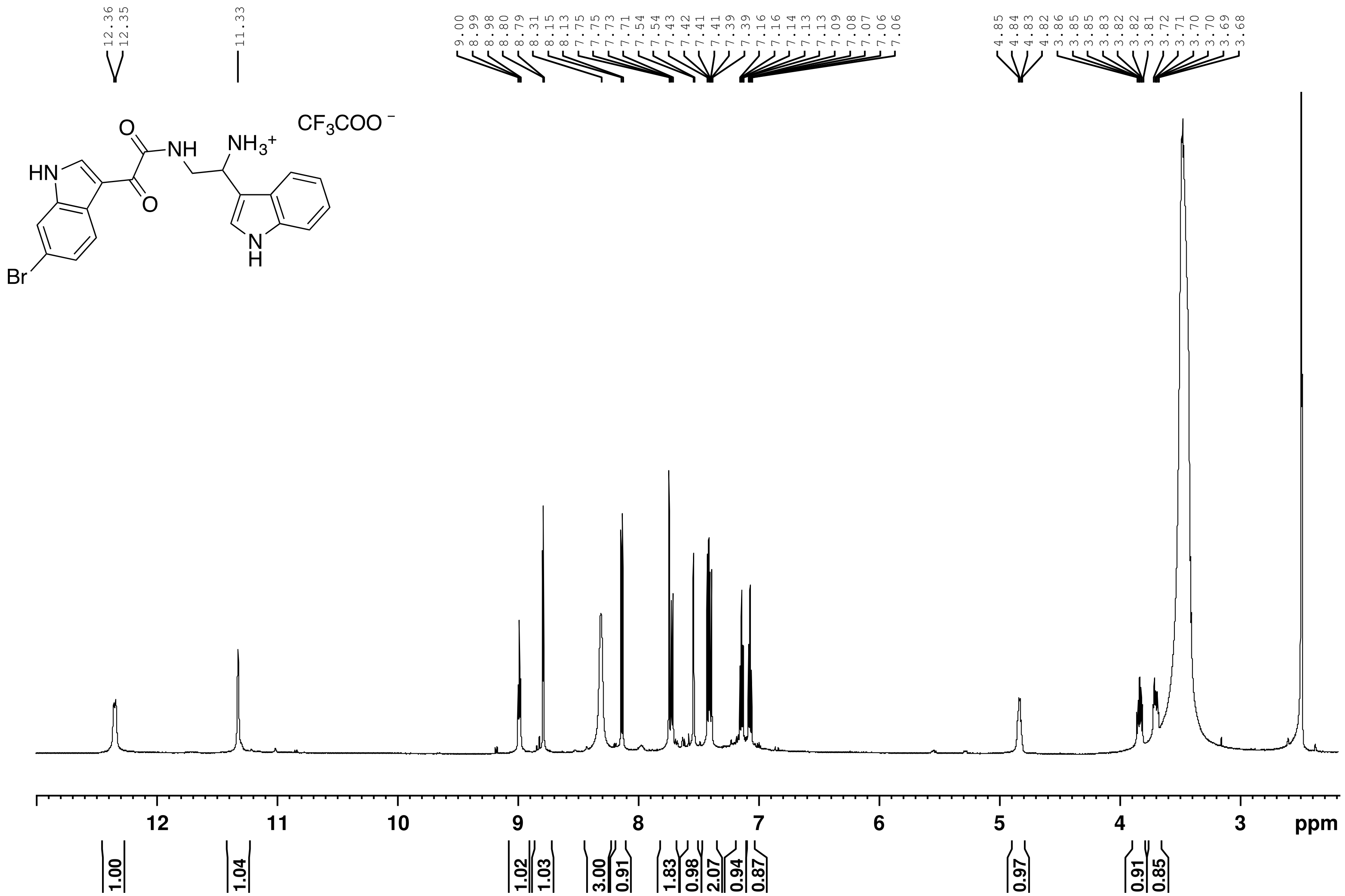

Figure S70. ^{1}H NMR spectrum (600 MHz) of 3,4-seco-6″-debromohamacanthin B (**19**) in DMSO-d_6

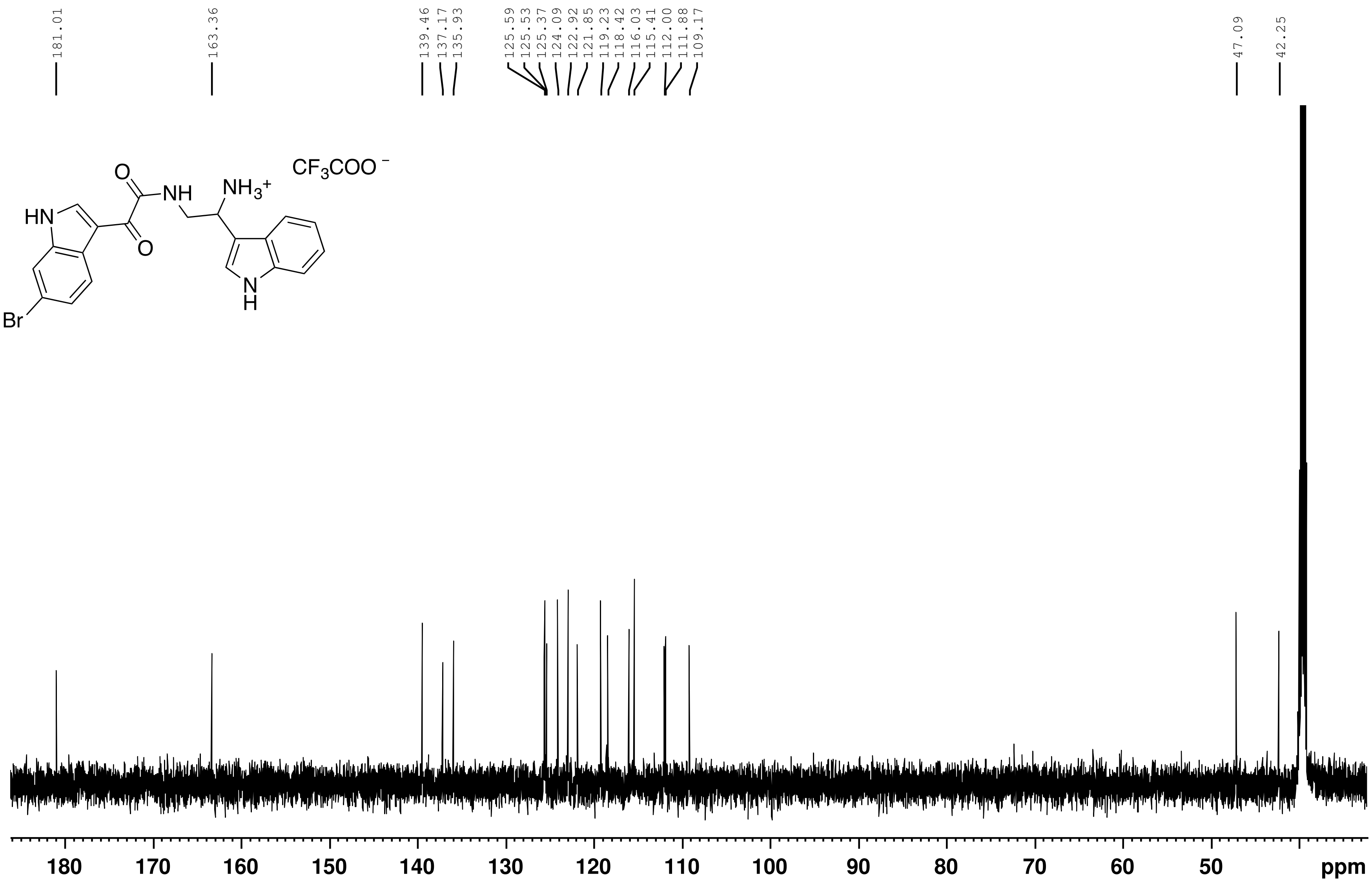

Figure S71. ^{13}C NMR spectrum (150 MHz) of 3,4-seco-6″-debromohamacanthin B (**19**) in DMSO-d_6

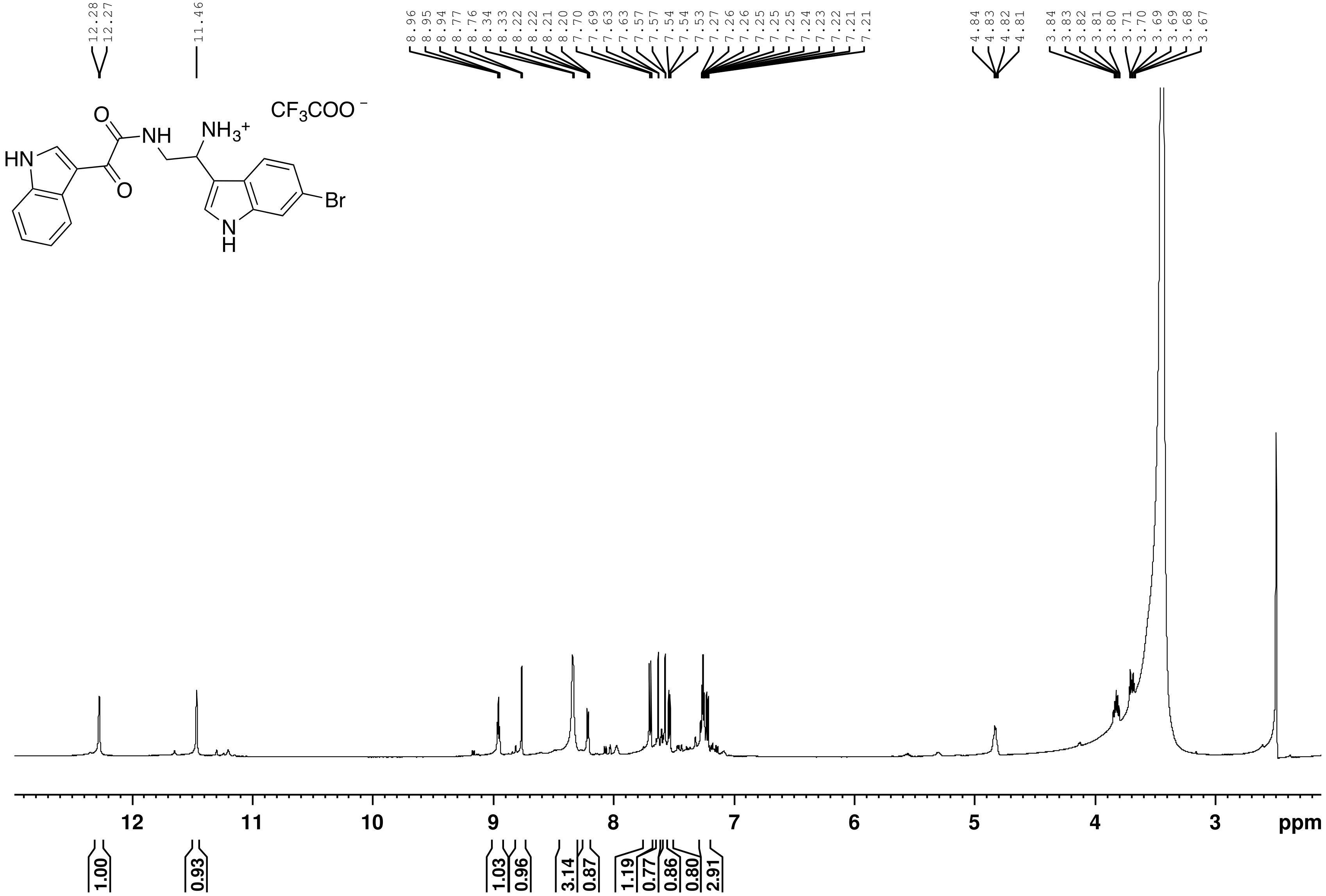

Figure S72. ^{1}H NMR spectrum (600 MHz) of 3,4-seco-6′-debromohamacanthin B (**20**) in DMSO-d_6

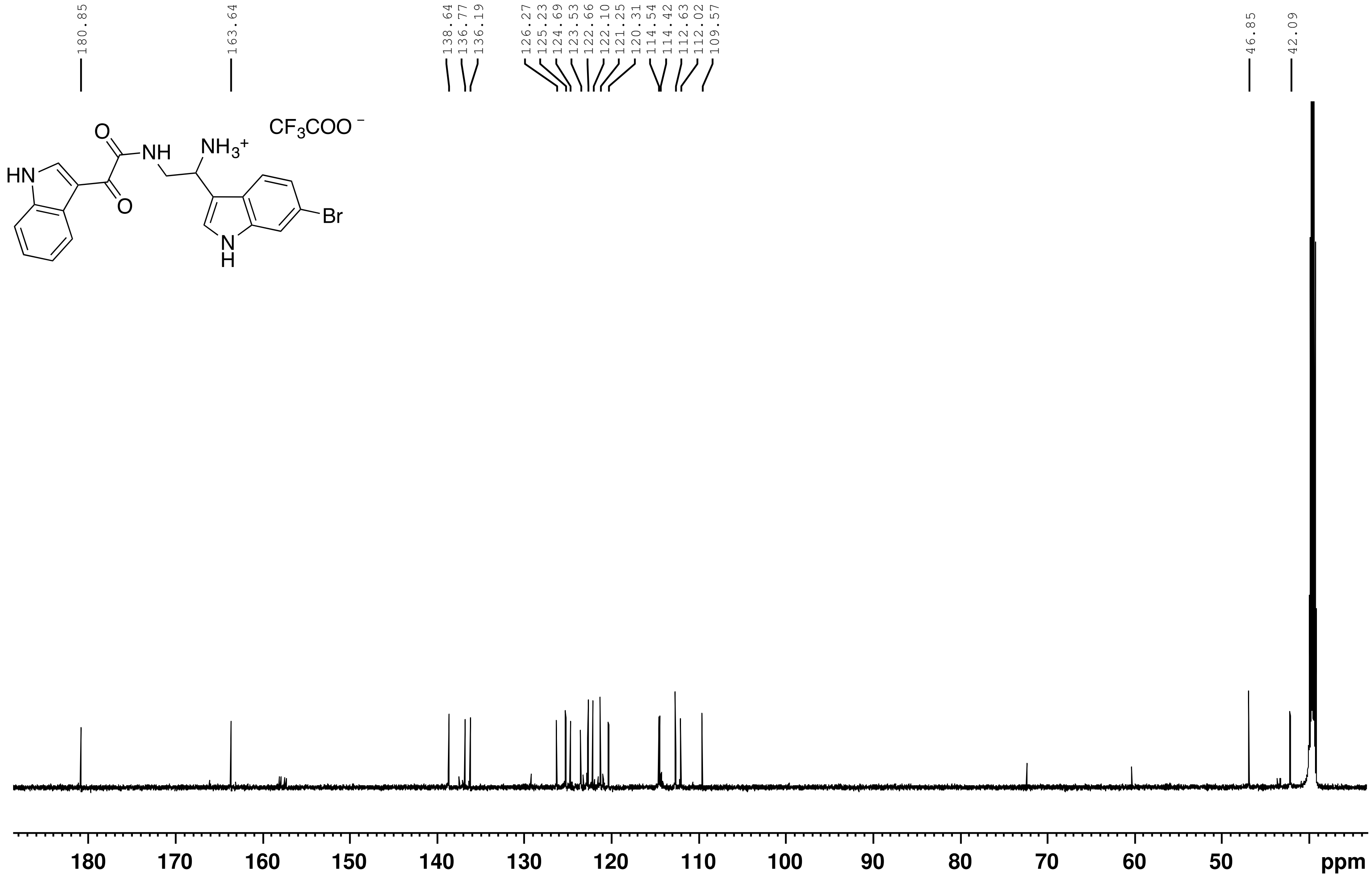

Figure S73. ^{13}C NMR spectrum (150 MHz) of 3,4-seco-6′-debromohamacanthin B (**20**) in DMSO-d_6

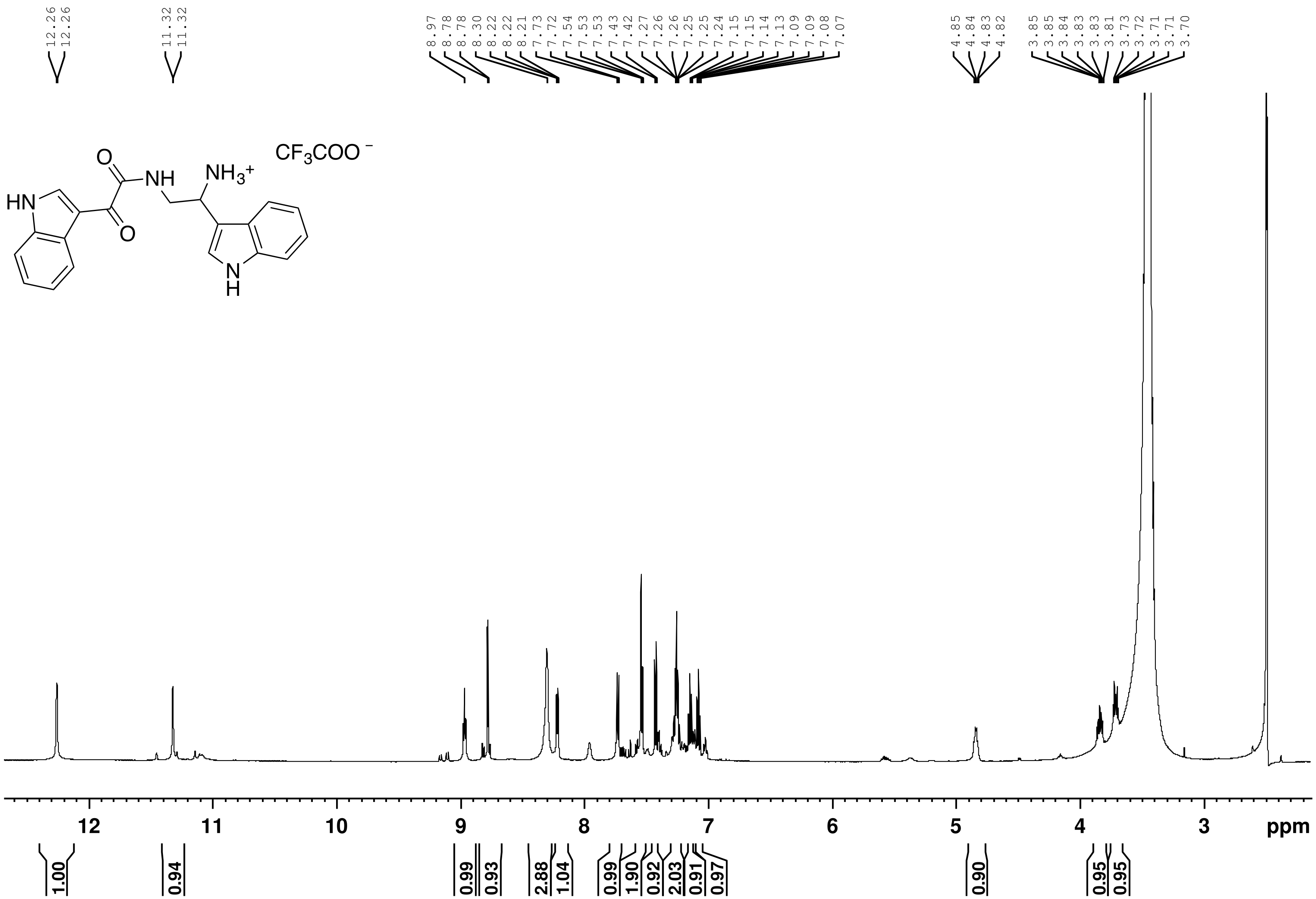

Figure S74. ^{1}H NMR spectrum (600 MHz) of 3,4-seco-6′,6″-didebromohamacanthin B (**21**) in DMSO-d_6

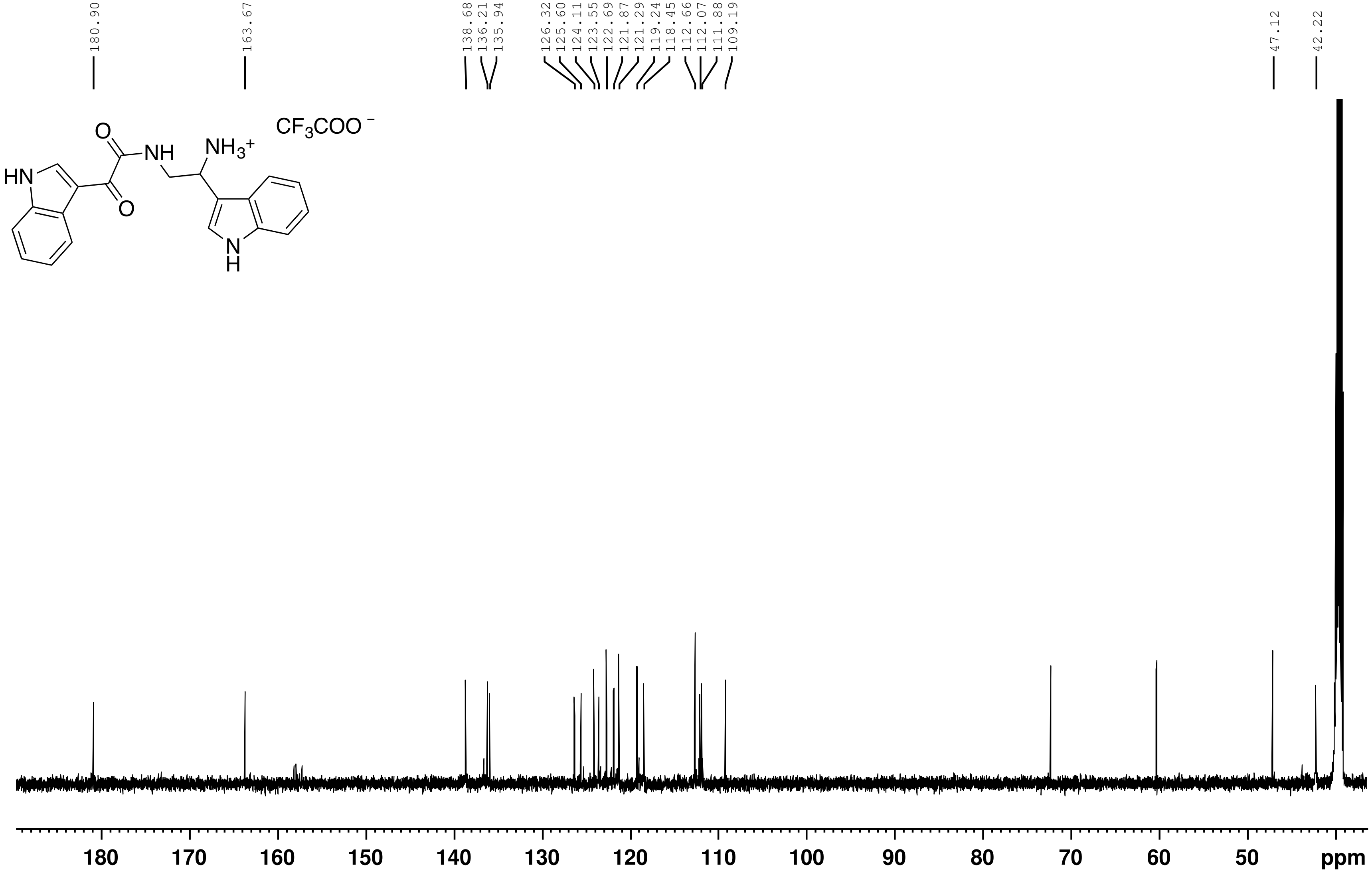

Figure S75. ^{13}C NMR spectrum (150 MHz) of 3,4-seco-6′,6″-didebromohamacanthin B (**21**) in DMSO-d_6

Cell path 10 mm (UV - red trace, TD-DFT calculation - orange trace)

References:

(1) Bokesch, H. R.; Pannell, L. K.; McKee, T. C.; Boyd, M. R., *Tetrahedron Lett.* **2000,** *41*, 6305-6308.

(2) Ma, Y.; Yakushijin, K.; Miyake, F.; Horne, D., *Tetrahedron Lett.* **2009,** *50*, 4343-4345.

(3) Tsujii, S.; Rinehart, K. L.; Gunasekera, S. P.; Kashman, Y.; Cross, S. S.; Lui, M. S.; Pomponi, S. A.; Diaz, M. C., *J. Org. Chem.* **1988,** *53*, 5446-5453.

(4) Shin, J.; Seo, Y.; Cho, K. W.; Rho, J.-R.; Sim, C. J., *J. Nat. Prod.* **1999,** *62*, 647-649.

(5) Rasmussen, T.; Jensen, J.; Anthoni, U.; Christophersen, C.; Nielsen, P. H., *J. Nat. Prod.* **1993,** *56*, 1553-1558.

(6) Bao, B.; Zhang, P.; Lee, Y.; Hong, J.; Lee, C.-O.; Jung, J., *Mar. Drugs* **2007,** *5*, 31-39.

(7) Bao, B.; Sun, Q.; Yao, X.; Hong, J.; Lee, C.-O.; Sim, C. J.; Im, K. S.; Jung, J. H., *J. Nat. Prod.* **2005,** *68*, 711-715.

(8) Bao, B.; Sun, Q.; Yao, X.; Hong, J.; Lee, C.-O.; Cho, H. Y.; Jung, J. H., *J. Nat. Prod.* **2007,** *70*, 2-8.

(9) Jiang, B.; Yang, C.-G.; Wang, J., *J. Org. Chem.* **2001,** *66*, 4865-4869.

(10) Gunasekera, S. P.; McCarthy, P. J.; Kelly-Borges, M., *J. Nat. Prod.* **1994,** *57*, 1437-1441.
